# Asymmetric Synthesis of Tetrahydroisoquinoline Derivatives through 1,3-Dipolar Cycloaddition of *C*,*N*-Cyclic Azomethine Imines with Allyl Alkyl Ketones

**DOI:** 10.3390/molecules26102969

**Published:** 2021-05-17

**Authors:** Guipeng Feng, Guoyang Ma, Wenyan Chen, Shaohong Xu, Kaikai Wang, Shaoyan Wang

**Affiliations:** 1 School of Chemical Engineering, University of Science and Technology Liaoning, Anshan 114051, China; fengguipengheda@163.com; 2 School of Pharmacy, Xinxiang University, Xinxiang 453000, China; maguoyang0903@gmail.com (G.M.); c15736992481@163.com (W.C.); xu_shaohong@126.com (S.X.)

**Keywords:** 1,3-dipolar cycloaddition, asymmetric, azomethine imines

## Abstract

A [3 + 2] 1,3-Dipolar cycloaddition of *C*,*N*-cyclic azomethine imines with allyl alkyl ketones has been achieved. The reaction proceeds under mild conditions and tolerates a wide range of functional groups. An array of tetrahydroisoquinoline derivatives is generally constructed with good diastereoselectivities and enantioselectivities (up to >25:1 dr, >95% ee). Moreover, the absolute configuration of the product was previously determined by using the quantum electronic circular dichroism calculation and ECD spectrum method.

## 1. Introduction

**Figure 1:**
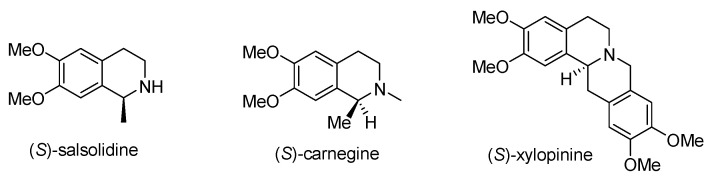
Examples of bioactive natural products containing chiral C1-substituted tetrahydroisoquinolines.

A variety of isoquinoline alkaloids [1,2,3] exist in many natural products and drugs, and have a broad range of clinical applications, exhibiting a broad range of biological activities such as antitumor, anti-HIV, antibiotic, antifungal, antivirus, anti-inflammatory, anticoagulation, and bronchodilation, and can also act on the central nervous system [4,5,6,7,8]. In particular, it is tremendously noteworthy that all the above-illustrated bioactive tetrahydroisoquinolines have a chiral stereocenter at the C1-position [9,10,11,12]. Such representative examples include (*S*)-salsolidine [13], (*S*)-carnegine [14], (*S*)-xylopinine [15] (in Figure 1), and so on. Novel *C*,*N*-cyclic azomethine imines as efficient 1,3-dipoles [16,17], are readily accessible, stable compounds that have been employed recently in various metal-catalyzed and organocatalytic 1,3-dipolar cycloadditions (1,3-DCs) [18,19,20,21]. These dipoles can be easily prepared and give access to pharmaceutically attractive chiral substituted tetrahydroisoquinoline skeletons.

**Scheme 1:**
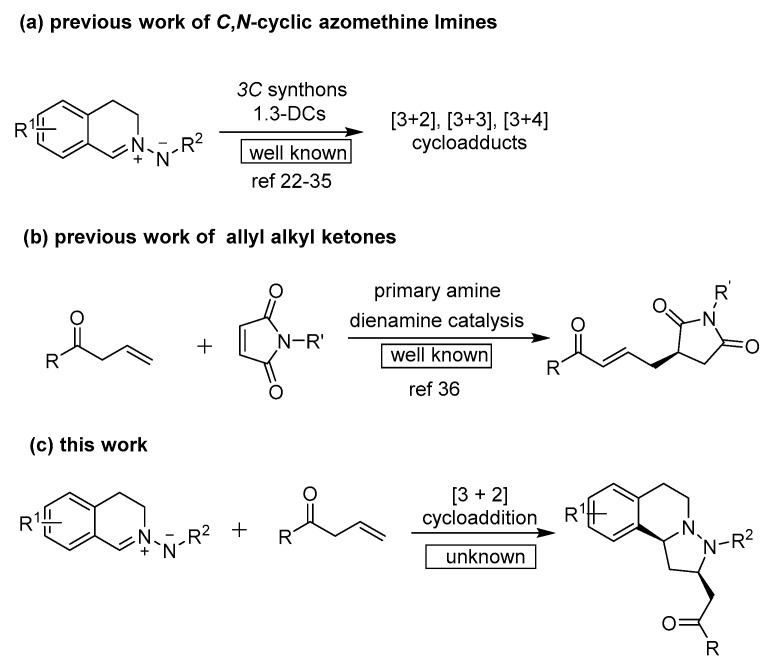
Previous reports and our protocol.

In 2010, the Maruoka group uncovered a promising example of using *C*,*N*-cyclic azomethine imines as prochiral electrophiles to react with α,β-unsaturated aldehydes to construct a tetrahydroisoquinoline scaffold catalyzed by a titanium–BINOLate complex [22]. Shortly after, the Maruoka group disclosed the first example of using of vinyl ethers and *C*,*N*-cyclic azomethine imines catalyzed by chiral Brønsted acids to synthesize tetrahydroisoquinolines through the enantioselective organocatalytic inverse-electron-demand 1,3-DCs [23]. In 2014, the Wang group developed *C*,*N*-cyclic azomethine imines with α,β-unsaturated aldehydes through dienamine-mediated enantioselective [3 + 2] 1,3-dipolar cycloaddition catalyzed by a chiral prolinol silyl ether catalyst [24,25]. In addition, the *C*,*N*-cyclic azomethine imine substrates were recently used in 1,3-DCs with *N*-arylmaleimides, allenoates, azlactones, bromo-substituted Morita−Baylis−Hillman adducts of isatins, α,β-unsaturated nitriles, 3-nitroindoles, ortho-quinone methides through [3 + 2] or [4 + 3] annulation reactions [26,27,28,29,30] and catalyst free [5 + 1] cycloaddition with isocyanides [31], and with Morita−Baylis−Hillman carbonates by phosphine catalysts or with *N*-benzyl azomethine ylide or with azaoxyallyl cations through [3 + 3] cycloaddition [32,33,34], and [3 + 1] cycloaddition with isocyanides (in Scheme 1a) [35]. However, to the best of our knowledge, no example of a catalytic asymmetric 1,3-dipolar cycloaddition reaction using allyl alkyl ketones with *C*,*N*-cyclic azomethine imines has been reported. Previous success by the Chen group, using chiral primary amine catalytic asymmetric γ-regioselective vinylogous Michael addition of allyl alkyl ketones with maleimides through dienamine catalysis, has been developed (in Scheme 1b) [36]. Herein, we report the first chiral primary amine-catalyzed enantioselective [3 + 2] 1,3-dipolar cycloaddition of allyl alkyl ketones with *C*,*N*-cyclic azomethine imines to give a novel class of dinitrogen-fused heterocycles combining the biologically important tetrahydroisoquinoline core and pyrazolidine core (in Scheme 1c).

## 2. Results

In our initial attempt, we first examined the reaction of *N*,*N*-cyclic azomethine imine **1** with deconjugated 3-enone **5a** in the presence of DPEN catalyst **C1** (Table 1) in CHCl_3_, but no desired product was observed even at a higher temperature. We then turned our attention to *C*,*N*-cyclic azomethine imines **2, 3** and **4a**. Unfortunately, no matter what the temperature of the reaction raised from rt to reflux, no desired cycloaddition adduct was observed when we employed the **2** and **3** dipoles. To our delight, the reaction between *C*,*N*-cyclic azomethine imine **4a** and deconjugated 3-enone **5a** proceeded smoothly to give the desired product in high yield (85% yield, 62% ee and dr 3:2, entry 1, Table 1) and the reaction could not afford the desired product if no acid or no catalyst were added to the reaction [18] (entries 2 and 3). The results indicated that catalysts with acids are critical for the cycloaddition reaction. In addition, the β,γ-C=C bond could act as an inducing group for the formation of more stable extended dienamine species from deconjugated 3-enone substrates **5a** and activating the γ-site and furnishing the following vinylogous 1,3-DCs process.

We then turned our attention towards the use of chiral primary amine catalyst to improve the reaction enantioselectivity and diastereoselectivity. The reaction was tested in the presence of a number of primary amine catalysts (entries 4–10). The corresponding product **6aa** was then isolated in high yield (89%), good enantioselectivity (80% ee), and with good diastereoselectivity (dr 12.5:1) through **C2** catalyst in CHCl_3_ at room temperature (entry 4). In contrast, the reaction afforded poor to moderate enantioselectivity (11–76%) when used with other catalysts (entries 5–11). The chiral prolinol silyl ethers as the catalyst could not produce the product (entry 12). A screening of the solvents showed that performing the reaction to improve the ee and the dr (entry 13–19) with **C2** as the catalyst. The reaction proceeded with higher yield (91%) and good enantioselectivity (80%) and excellent diastereoselectivity (dr 25:1) in DCE at room temperature (entry 14). However, only poor or moderate diastereoselectivities were observed in other solvents (entry 13, 15–19). Furthermore, the effect of acid additives was also studied. The presence of 20 mol% *o*-fluorobenzoic acid (OFBA) enhanced the ee value to 84% and the yield also increased to 92% (entry 20). A screening of the temperature showed that performing the reaction at 0 °C reduced the ee value and the yield (entry 20). The yield and stereoselectivity could not be further improved when the reaction used the other acid additives (entries 21–23). On the basis of the above-mentioned results, the reaction conditions were established to 1.0 equivalent of **4**, 2.0 equivalents of **5**, 10 mol% of **C2** and 20 mol% of OFBA in DCE at room temperature for 12 h.

## 3. Discussion

**Figure 2:**
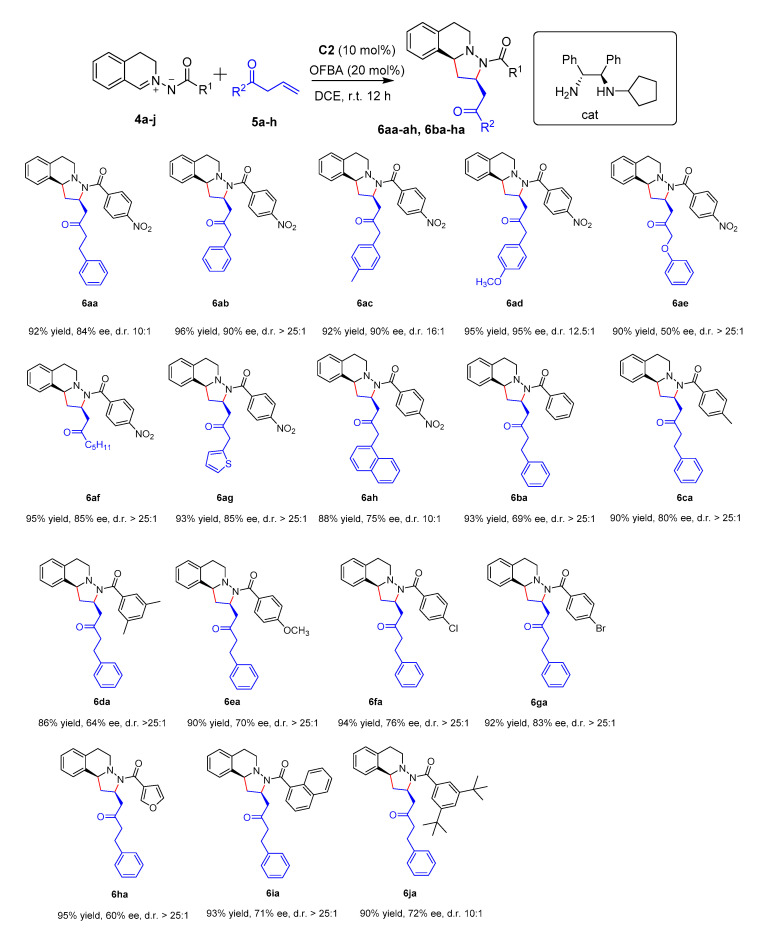
Substrate scope of the double 1,3-dipolar cycloaddition ^a^. ^a^ Unless noted otherwise, reactions were performed with **4** (0.1 mmol), **5** (0.2 mmol), amine **C2** (10 mol%), and OFBA (20 mol%) in DCE (1 mL) at rt.

With the optimized conditions in hand, the generality of the reaction was evaluated. A range of substrates was shown to be compatible with the developed protocol for the [3 + 2] cycloaddition of *C*,*N*-cyclic azomethine imines to allyl alkyl ketone by using **C2** as catalyst. As summarized in Figure 2 (Figures S2 and S3 Supplementary Material, respectively), not only aromatic alkyl allyl ketones but also long aliphatic chain allyl ketones could all be employed successfully to afford the products **6aa–ah** in high yields (88–96%), moderate to high enantioselectivities (50–96%), and high diastereoselectivities (dr 10:1 to >25:1) From these results, we found that the conditions were applicable to a wide variety of allyl alkyl ketones. With this promising result in hand, we then investigated the generality of the *C*,*N*-cyclic azomethine imines. The influence of the substituent of the benzoyl group on the nitrogen was examined. The benzoyl group bearing electron-withdrawing or -donating groups at the *para* position and *meta* position afforded the corresponding products in high yields and steroselectivities (**6ba** to **6ga**). We found that some electron-withdrawing groups (−NO_2_, −Cl, −Br) on the aromatic moiety were more effective than some donating groups (−CH_3_, −OCH_3_, 3,5-Me_2_) to afford the products in yields and steroselectivities, respectively (**6aa** to **6ga**). From these results, it was determined that the electron density of the *N’*-acyl moiety played an important role in trapping the β-position of the carbon−carbon double bond intermediate for the 1,3-DCs. This study prompted us to examine the influence of structurally different *N’*-acyl moiety azomethine imines. The furoyl and naphthoyl groups on the nitrogen were also tolerated, giving the desired products in high yields, moderate enantioselectivities, and high diastereoselectivities, respectively (**6ha** 95% yield, 60% ee, dr > 25:1 and **6ia** 93% yield, 71% ee, dr > 25:1). An increase in the steric bulk of the *N’*-acyl moiety was also tolerated to afford the product **6ja** in high yields (90%) with 72% ee value and dr 10:1.

**Figure 3:**
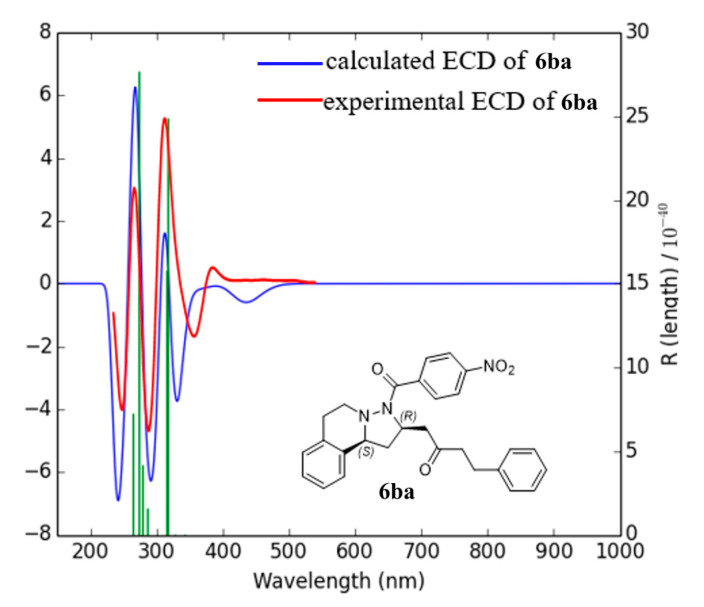
Comparison of the experimental ECD spectrum of chiral **6ba** (the red line) with the calculated one of (*R*,*S*)-**6ba** (the blue line). The green line is the position of the peak in horizontal axis.

As we failed to obtain single crystals suitable for X-ray crystallographic analysis to determine the absolute configuration of the products, the electronic circular dichroism (ECD) spectrum of chiral product **6ba** was recorded in methanol and compared with the theoretically calculated results [37,38,39]. As depicted in Figure 3, the experimental ECD spectrum matched quite well to the calculated one of (*R*,*S*)-**6ba**. Therefore, the stereogenic center of product **6ba** is probably in the (*R*,*S*) configuration.

**Scheme 2:**
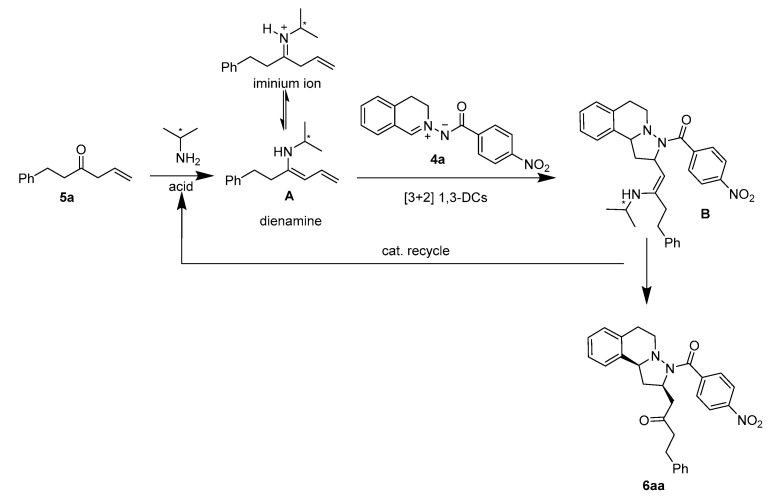
Plausible mechanism.

As shown in Scheme 2, we proposed a plausible catalytic cycle to explain the reaction mechanism. The condensation of a chiral primary amine catalyst with allyl phenylethyl ketone **5a** would lead to the formation of the iminium-ion, which could form the dienamine intermediate **A** [40,41,42]. The dienamine intermediate **A** reacts with the *C*,*N*-cyclic azomethine **4a** by a 1,3-dipolar cycloaddition to generate the polycyclic intermediate **B**. Subsequently, an acid-catalyzed elimination step converts intermediate **B** to the final product **6aa**.

## 4. Materials and Methods

NMR data were obtained for ^1^H at 400 MHz or 600 MHz, and for ^13^C at 100 MHz or 150 MHz. The data are presented as follows: chemical shift was reported in ppm from tetramethylsilane with the solvent resonance as the internal standard in CDCl3 solution, integration, multiplicity (br = broad, s = singlet, d = doublet, t = triplet, q = quartet, m = multiplet) and coupling constant in Hertz (Hz). ESI HRMS was recorded on a Waters SYNAPT G2. In each case, enantiomeric excesses (ee) were determined by chiral high-performance liquid chromatography (chiral HPLC) that were Daicel Chiralpak AD-H Column (250 × 4.6 mm), Chiralpak IA Column (250 × 4.6 mm), or Chiralpak IC Column (250 × 4.6 mm). UV detection was monitored at 220 nm or 254 nm. Optical rotation was measured in CHCl3 solution at 20 °C. Column chromatography was performed on silica gel (200–300 mesh) eluting with ethyl acetate and petroleum ether. TLC was performed on glass-backed silica plates. UV light, I_2_, and solution of potassium permanganate were used to visualize products. All chemicals were used without purification as commercially available unless otherwise noted. Petroleum ether (PE) and ethyl acetate (EtOAc) were distilled. THF was freshly distilled from sodium/benzophenone before use. Experiments involving moisture and/or air sensitive components were performed under a positive pressure of argon in oven-dried glassware equipped with a rubber septum inlet. Dried solvents and liquid reagents were transferred by oven-dried syringes.

The 1,3-dipoles of **1** [43], **2** [44], **3** [45] and **4** [46,47,48,49,50,51] were synthesized according to the literature methods. The substrates **5a−h** were prepared according to the literature procedures [36,52,53,54,55,56,57]. Catalyst **C1** was commercially available. Catalysts **C2**–**C8** were synthesized according to the literature procedures [58,59]. The *C*,*N*-cyclic azomethine imines **4** (0.1 mmol), catalyst **C2** (2.8 mg, 0.01 mmol), *o*-fluorobenzoic acid (2.8 mg, 0.02 mmol) were dissolved in DCE (1.0 mL) and allyl ketone **5** (0.2 mmol) was added. Then, the mixture was stirred at rt for 12 h. After completion, the mixture was evaporated and the resulting crude residue was purified by column chromatography on silica gel eluting with petroleum ether/ethyl acetate (10:1 to 5:1) to afford the chiral product **6**.

## 5. Conclusions

In conclusion, we have developed the first chiral primary amine-catalyzed enantioselective [3 + 2] 1,3-dipolar cycloaddition of allyl alkyl ketones with *C*,*N*-cyclic azomethine imines via induced dienamine catalysts to give a novel class of dinitrogen-fused heterocycles combining the biologically important tetrahydroisoquinoline core and pyrazolidine core. The reaction affords a tetrahydroisoquinoline derivative in high yield with moderate to high enantioselectivities (up to 96%) and high diastereoselectivities. In addition, this reaction provides an efficient method for constructing diverse and complex chiral tetrahydroisoquinolines compounds. Research into further applications of this enantioselective 1,3-dipolar cycloaddition with *C*,*N*-cyclic azomethine imines is in progress.

## Figures and Tables

**Table 1:** Optimization of reaction conditions ^a^.

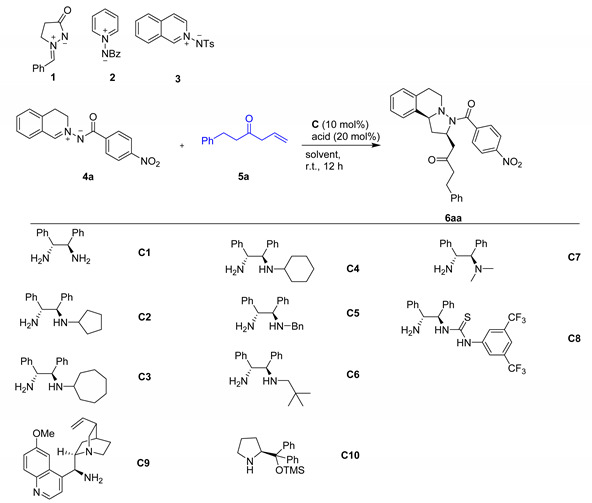						
Entry	Solvent	Catalyst	Acid	Yield (%) ^b^	ee (%) ^c^	dr ^d^
1	CHCl_3_	C1	benzoic acid	85	62	3:2
2	CHCl_3_	C1	none	n.r.	-	-
3	CHCl_3_	none	benzoic acid	n.r.	-	-
4	CHCl_3_	C2	benzoic acid	89	80	12.5:1
5	CHCl_3_	C3	benzoic acid	91	76	>25:1
6	CHCl_3_	C4	benzoic acid	90	72	>25:1
7	CHCl_3_	C5	benzoic acid	86	38	>25:1
8	CHCl_3_	C6	benzoic acid	85	59	>25:1
9	CHCl_3_	C7	benzoic acid	87	44	>25:1
10	CHCl_3_	C8	benzoic acid	88	11	5:1
11	CHCl_3_	C9	benzoic acid	56	20	>25:1
12	CHCl_3_	C10	benzoic acid	n.r.	-	-
13	Toluene	C2	benzoic acid	90	85	1:1
14	DCE	C2	benzoic acid	91	80	25:1
15	CH_3_CN	C2	benzoic acid	72	28	2:1
16	THF	C2	benzoic acid	65	50	6:1
17	Dioxane	C2	benzoic acid	87	77	1:1
18	Et_2_O	C2	benzoic acid	trace	-	-
19	EA	C2	benzoic acid	74	70	1:1
20	DCE	C2	*o*-fluorobenzoic acid	92 (85) ^e^	84 (64) ^e^	10:1
21	DCE	C2	salicylic acid	90	65	4:1
22	DCE	C2	*p*-nitrobenzoic acid	82	68	15.7:1
23	DCE	C2	*p*-methoxybenzoic acid	75	82	6:1

^a^ Unless noted otherwise, reactions were performed with **4a** (0.1 mmol), **5a** (0.2 mmol), amine **C** (10 mol%), and acid (20 mol%) in solvent (1 mL) at rt. ^b^ Isolated yield. ^c^ Determined by chiral HPLC analysis. ^d^ Determined by crude NMR analysis. ^e^ The reaction was conducted at 0 °C for 24 h.

## Data Availability

Data are contained within the article or Supplementary materials.

## Supplementary Materials

The following are available online. Figure S1: General procedure for the preparation of the *C*,*N*-cyclic azomethines imines and its analogs, Figure S2: General procedure for catalytic asymmetric 1,3-DCs, Figure S3: Copies of NMR spectra and HPLC spectra.

## Sample Availability

Samples of the compounds are available from the authors.
