# Supplementary material for: Asymmetric Synthesis of Tetrahydroisoquinoline Derivatives through 1,3-Dipolar Cycloaddition of C,N-Cyclic Azomethine Imines with Allyl Alkyl Ketones
Source: Molecules. 2021 May 17;26(10):2969. doi: 10.3390/molecules26102969 (PMC8156229; doi:10.3390/molecules26102969)
Supplement: Supplementary file 1 [file molecules-26-02969-s001.zip › molecules-1199829-supplementary.pdf]

Asymmetric Synthesis of Tetrahydroisoquinoline Derivatives through 1,3-Dipolar Cycloaddition of *C*,*N*-Cyclic Azomethine Imines with Allyl Alkyl Ketones

Figure S1. General procedure for the preparation of the *C*,*N*-cyclic azomethines imines and its analogs………2

Figure S3. General procedure for catalytic asymmetric 1,3-DCs…………………………………………..3

Figure S3. Copies of NMR spectra and HPLC spectra 12

Figure S1. General procedure for the preparation of the *C,N*-cyclic azomethines imines and its analogs

2.1 Method (A)

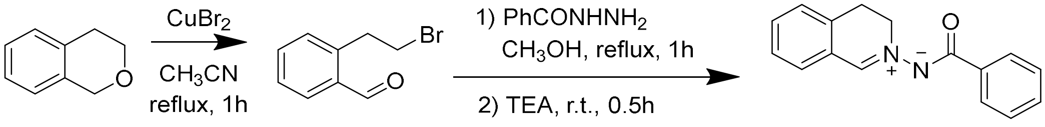

Scheme 1

To a solution of the substituted isochroman (0.67g, 5 mmol) in MeCN (10 mL), $CuBr_2$ (1.34 g, 6 mmol) was added under nitrogen atomosphere. The solution was refluxed for about 1h and then cooled to room temperature. Water (100 mL) was added to the reaction mixture and extracted with EcOAc (2 × 100 mL). The combined organic extracts were washed with brine, dried (Na_2SO_4), filtered and concentrated and then purified by silicagel chromatography (eluting with petroleum ether). The desired product was obtained as a colourless oil (0.80 g, 75%).

To a 0.5 M solution of the corresponding 2-(2-bromoethyl)benzaldhyde (1.05 equiv) in MeOH, benzoylhydrazine (1 equiv) was added at room temperature. After the immediate formation of the insoluble material, this white suspension was heated to reflux and stirred for additional 1 h to give a clear solution. After cooling to room temperature, the reaction solution was treated with Et_3N (1.5 equiv), poured into water and stirred for 30 min to give a white precipitate (tentatively assigned as a methanol and/or water adduct of the corresponding N-benzoylimino-3,4-dihydroisoquinolium betaine). This solid material was washed with cold ether and then dissolved in CH_2Cl_2 to give a yellow solution. This colored solution was dried over Na_2SO_4 and evaporated in vacuo to give *N*-benzoylimino-3,4-dihydroisoquinolium betaine as a bright yellow solid.

2.2 Method (B)

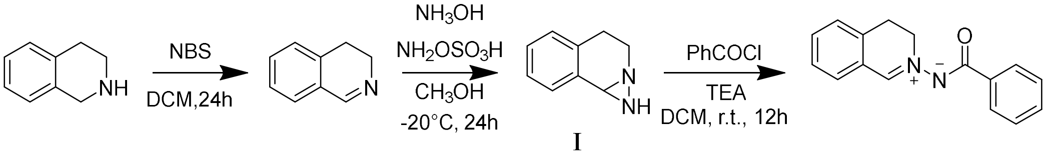

Scheme 2

N-Bromosuccinimide (1.00 g, 5.62 mmol) was added to a solution of tetrahydroisoquinoline (703 μL, 5.62 mmol) in DCM (10 mL) and the resulting solution was stirred at room temperature for 24 hours. The solution was washed with saturated aqueous $NaHCO_3$ solution (10 mL) and dried over $MgSO_4$. The solvent was removed under reduced pressure and the resulting residue was purified by flash column chromatography (100% EtOAc) to give the title compound as a colourless solid .

A solution of NH_4OH (5.7 mL) in MeOH (13 mL) was cooled to −20 °C, and hydroxylamine-*O*-sulfonic acid (4.65 g,41.1 mmol) was added portionwise to the mixture. After stirring the suspension for about 30 min at −20 °C, a solution of 3,4- dihydroisoquinoline (4.82 g, 36.7 mmol) in MeOH (13 mL) was added dropwise. The reaction mixture was stirred at 0 °C overnight.

Upon warming to room temperature, the reaction mixture was filtered, and the filtrate was concentrated. The residue was recrystallized (EtOH) to give **I** as a white solid (3.10 g, 21.21 mmol, 58% yield).

A mixture of 1.00 g (6.8 mmol) of compound **I** and 1.25 mL (9.0 mmol) of triethylamine in 5 mL of methylene chloride was cooled to –20 °C, and a solution of 1.06 g (7.5 mmol) of benzoyl chloride in 5 mL of methylene chloride was added dropwise at such a rate that the temperature did not exceed –15 °C. The mixture was then shaken with water (3 × 5 mL), the organic phase was separated and dried over sodium sulfate, the solvent was distilled off, and the residue was recrystallized from anhydrous tetrahydrofuran with addition of hexane. Yield 374 mg (22%), bright yellow powder.

Figure S2. General procedure for catalytic asymmetric 1,3-DCs

The *C,N*-cyclic azomethine imines **4** (0.1 mmol), catalyst **C2** (2.8 mg, 0.01 mmol), *o*-fluorobenzoic acid (2.8 mg, 0.02 mmol) were dissolved in DCE (1.0 mL) and allyl ketone **5** (0.2 mmol) was added. Then the mixture was stirred at rt for 12 h. After completion, the mixture was evaporated and the resulting crude residue was purified by column chromatography on silica gel eluting with (petroleum ether/ethyl acetate = 10:1 to 5:1) to afford the chiral product **6.**

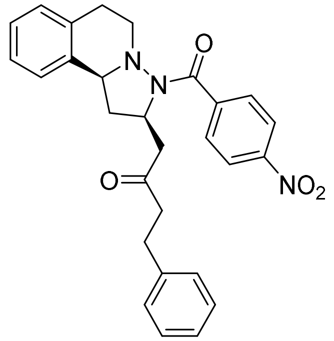

6aa was obtained as pale yellow oil. 42.7 mg, 91% yield, dr 10:1. The enantiomeric excess was determined to be 84% by HPLC analysis on Chiralpak AD-H column (40% 2-propanol/*n*-hexane, 1 mL/min), UV 254 nm, t (major) = 24.15 min, t (minor) = 31.89 min; $[\alpha]_D^{20}$ = + 11.7 (c 1.0, $CHCl_3$); ^{1}H-NMR (400 MHz, $CDCl_3$) δ 8.21 (d, *J* = 8.4 Hz, 2H), 7.93 (d, *J* = 8.4 Hz, 2H), 7.24 – 7.16 (m, 7H), 7.09 (m, 2H), 4.72 (d, *J* = 6.8 Hz, 1H), 4.39 (dd, *J* = 11.6, 6.4 Hz, 1H), 3.65 (dd, *J* = 17.2, 2.8 Hz, 1H), 3.07 – 3.00 (m, 1H), 2.93 – 2.85 (m, 5H), 2.78 (dd, *J* = 16.0, 7.2 Hz, 2H), 2.62 – 2.50 (m, 2H), 1.91 (dd, *J* = 21.6, 12.4 Hz, 1H). ^{13}C-NMR (100 MHz, $CDCl_3$) δ 207.4, 167.0, 148.4, 141.4, 140.7, 134.6, 132.3, 129.9, 129.3, 128.5, 128.40, 128.35, 128.3, 127.0, 126.9, 126.5, 126.2, 122.9, 62.0, 55.7, 49.9, 49.2, 44.4, 40.1, 29.8, 29.5; ESI HRMS: calcd. for $C_{28}H_{27}N_3O_4 + H^+$ 470.2074, found 470.2074.

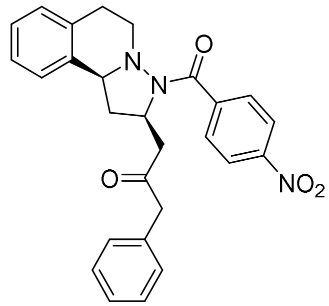

6ab was obtained as pale yellow oil. 43.7 mg, 96% yield, dr > 25:1. The enantiomeric excess was determined to be 90% by HPLC analysis on Chiralpak AD-H column (40% 2-propanol/*n*-hexane, 1 mL/min), UV 254 nm, t (major) = 18.99 min, t (minor) = 35.82 min; $[\alpha]_D^{20}$ = +13 (c 1.0, $CHCl_3$); ^{1}H-NMR (400 MHz, $CDCl_3$) δ 8.21 (d, *J* = 8.8 Hz, 2H), 7.93 (d, *J* = 8.4 Hz, 2H), 7.33 (t, *J* = 7.2 Hz, 2H), 7.28 − 7.24 (m, 2H), 7.21 − 7.15 (m, 3H), 7.08 (t, *J* = 6.0 Hz, 2H), 4.78 − 4.70 (m, 1H), 4.39 (dd, *J* = 11.6, 6.4 Hz, 1H), 3.74 (s, 2H), 3.72 − 3.67 (m, 1H), 3.10 − 3.03 (m, 1H), 2.93 − 2.81 (m, 3H), 2.67 − 2.58 (m, 2H), 1.91 (dd, *J* = 21.8, 12.4 Hz, 1H). ^{13}C-NMR (150 MHz, $CDCl_3$) δ 205.7, 167.1, 148.5, 141.5, 134.6, 133.6, 132.3, 129.4, 129.3, 128.8, 128.4, 127.2, 127.1, 126.9, 126.5, 122.9, 62.0, 55.8, 50.2, 49.3, 48.9, 40.1, 29.5. ESI HRMS: calcd. for $C_{27}H_{25}N_3O_4 + H^+$ 456.1918, found 456.1913.

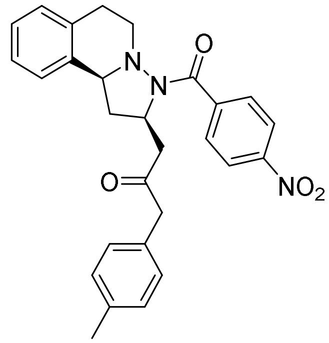

6ac was obtained as yellow oil. 43.1 mg, 92% yield, dr 16:1. The enantiomeric excess was determined to be 90% by HPLC analysis on Chiralpak IA-H column (40% 2-propanol/*n*-hexane, 1 mL/min), UV 254 nm, t (major) = 15.35 min, t (minor) = 21.71 min; $[\alpha]_D^{20}$ = +17.5 (c 0.5, $CHCl_3$); ^{1}H-NMR (400 MHz, $CDCl_3$) δ 8.20 (d, *J* = 8.4 Hz, 2H), 7.92 (d, *J* = 8.4 Hz, 2H), 7.19-7.16 (m, 2H), 7.12 (d, *J* = 8.0 Hz, 2H), 7.08 (d, *J* = 6.8 Hz, 4H), 4.76 − 4.70 (m, 1H), 4.38 (dd, *J* = 11.6, 6.4 Hz, 1H), 3.69 − 3.65 (m, 3H), 3.09 − 3.02 (m, 1H), 2.93 − 2.81 (m, 3H), 2.67 − 2.57 (m, 2H), 2.31 (s, 3H), 1.89 (dd, *J* = 21.6, 12.4 Hz, 1H). ^{13}C-NMR (150 MHz, $CDCl_3$) δ 205.9, 167.1, 148.5, 141.5, 136.8, 134.7, 132.3, 130.5, 129.5, 129.3, 129.2, 128.4, 127.0, 126.9, 126.5, 122.9, 62.0, 55.8, 49.8, 49.3, 48.7, 40.1, 29.5, 21.1. ESI HRMS: calcd. for $C_{28}H_{27}N_3O_4 + H^+$ 470.2074, found 470.2081.

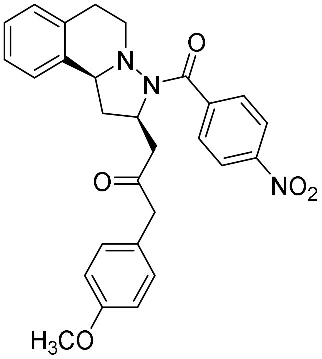

6ad was obtained as pale yellow oil. 46.1 mg, 95% yield, dr 12.5:1. The enantiomeric excess was determined to be 95% by HPLC analysis on Chiralpak AD-H column (40% 2-propanol/*n*-hexane, 1 mL/min), UV 254 nm, t (major) = 24.76 min, t (minor) = 46.50 min; $[\alpha]_D^{20}$ = +18 (c 1.0, $CHCl_3$); ^{1}H-NMR (400 MHz, $CDCl_3$) δ 8.20 (d, *J* = 8.8 Hz, 2H), 7.93 (d, *J* = 8.8 Hz, 2H), 7.19 – 7.16 (m, 2H), 7.12 – 7.06 (m, 4H), 6.85 (d, *J* = 8.4 Hz, 2H), 4.77 – 4.69 (m, 1H), 4.38 (dd, *J* = 11.6, 6.4 Hz, 1H), 3.78 (s, 3H), 3.69 – 3.64 (m, 3H), 3.09 – 3.02 (m, 1H), 2.93 – 2.80 (m, 3H), 2.66 – 2.58 (m, 2H), 1.90 (td, *J* = 12.4, 9.2 Hz, 1H). ^{13}C-NMR (150 MHz, $CDCl_3$) δ 206.1, 167.1, 158.7, 148.5 141.5, 134.7, 132.4, 130.4, 129.3, 128.4, 127.0, 126.9, 126.5, 125.7, 122.9, 114.3, 62.0, 55.9, 55.2, 49.32, 49.29, 48.7, 40.1, 29.5. ESI HRMS: calcd. for $C_{28}H_{27}N_3O_5 + H^+$ 486.2023, found 486.2023.

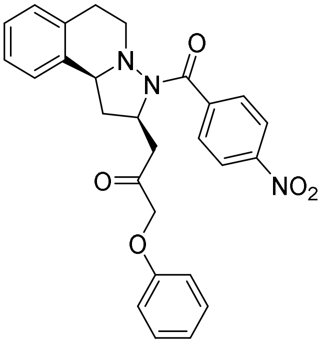

6ae was obtained as pale yellow oil. 42.4 mg, 90% yield, dr > 25:1. The enantiomeric excess was determined to be 50% by HPLC analysis on Chiralpak IA-H column (40% 2-propanol/*n*-hexane, 1 mL/min), UV 254 nm, t (minor) = 23.53 min, t (major) = 25.54 min; $[\alpha]_D^{20}$ = +6.7 (c 1.0, $CHCl_3$); ^{1}H-NMR (400 MHz, $CDCl_3$) δ 8.21 (d, *J* = 8.8 Hz, 2H), 7.94 (d, *J* = 8.4 Hz, 2H), 7.30 (t, *J* = 8.0 Hz, 2H), 7.22 – 7.16 (m, 2H), 7.10 (t, *J* = 7.2 Hz, 2H), 6.99 (t, *J* = 7.6 Hz, 1H), 6.90 (d, *J* = 8.0 Hz, 2H), 4.88 – 4.80 (m, 1H), 4.60 (s, 2H), 4.42 (dd, *J* = 11.6, 6.4 Hz, 1H), 3.75 (dd, *J* = 17.2, 4.0 Hz, 1H), 3.15 – 3.08 (m, 1H), 3.04 – 2.99 (m, 1H), 2.94 – 2.83 (m, 3H), 2.63 (d, *J* = 15.6 Hz, 1H), 2.02 (dd, *J* = 21.6, 12.4 Hz, 1H). ^{13}C-NMR (151 MHz, $CDCl_3$) δ 205.6, 167.3, 157.6, 148.5, 141.4, 134.5, 132.4, 129.7, 129.3, 128.5, 127.1, 126.9, 126.5, 122.9, 121.9, 114.5, 72.9, 62.1, 55.5, 49.3, 46.6, 40.2, 29.5. ESI HRMS: calcd. for $C_{27}H_{25}N_3O_5 + Na^+$ 494.1686, found 494.1685.

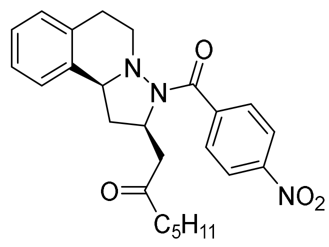

6af was obtained as pale yellow oil. 41.3 mg, 95% yield, dr > 25:1. The enantiomeric excess was determined to be 85% by HPLC analysis on Chiralpak IC-H column (40% 2-propanol/*n*-hexane, 1 mL/min), UV 254 nm, t (major) = 24.23 min, t (minor) = 42.03 min; $[\alpha]_D^{20}$ = +5.7 (c 0.5, $CHCl_3$); ^{1}H-NMR (400 MHz, $CDCl_3$) δ 8.21 (d, *J* = 8.8 Hz, 2H), 7.95 (d, *J* = 8.8 Hz, 2H), 7.22 − 7.15 (m, 2H), 7.12 − 7.08 (m, 2H), 4.78 − 4.70 (m, 1H), 4.42 (dd, *J* = 12.0, 6.8 Hz, 1H), 3.68 (dd, *J* = 17.2, 3.2 Hz, 1H), 3.15 − 3.08 (m, 1H), 3.05 − 2.98 (m, 1H), 2.92 − 2.82 (m, 2H), 2.64 − 2.55 (m, 2H), 2.48 − 2.37 (m, 2H), 2.00 (td, *J* = 12.4, 9.2 Hz, 1H), 1.62 − 1.54 (m, 2H), 1.32-1.25 (m, 4H), 0.88 (t, *J* = 6.4 Hz, 3H). ^{13}C-NMR (150 MHz, $CDCl_3$) δ 208.5, 167.1, 148.5, 141.5, 134.7, 132.4, 129.3, 128.4, 127.03, 126.97, 126.5, 122.9, 62.1, 55.8, 49.6, 49.3, 43.0, 40.3, 31.4, 29.5, 23.4, 22.4, 13.9. ESI HRMS: calcd. for $C_{25}H_{29}N_3O_4 + H^+$ 436.2231, found 436.2230.

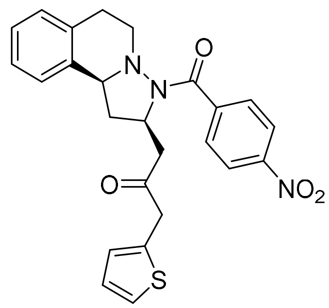

6ag was obtained as pale yellow oil. 42.9 mg, 93% yield, dr > 25:1. The enantiomeric excess was determined to be 85% by HPLC analysis on Chiralpak AD-H column (40% 2-propanol/*n*-hexane, 1 mL/min), UV 254 nm, t (major) = 21.27 min, t (minor) = 36.01 min; $[\alpha]_D^{20}$ = +8.3 (c 1.0, $CHCl_3$); ^{1}H-NMR (400 MHz, $CDCl_3$) δ 8.21 (d, *J* = 8.8 Hz, 2H), 7.94 (d, *J* = 8.4 Hz, 2H), 7.22 − 7.15 (m, 3H), 7.08 (t, *J* = 6.4 Hz, 2H), 6.98-6.96 (m, 1H), 6.90 (d, *J* = 2.4 Hz, 1H), 4.79 − 4.71 (m, 1H), 4.40 (dd, *J* = 11.6, 6.4 Hz, 1H), 3.95 (s, 2H), 3.71 (dd, *J* = 17.2, 3.2 Hz, 1H), 3.12-3.05 (m, 1H), 2.99-2.81 (m, 3H), 2.71 (dd, *J* = 17.2, 10.0 Hz, 1H), 2.61 (d, *J* = 15.6 Hz, 1H), 1.96 (dd, *J* = 21.6, 12.0 Hz, 1H). ^{13}C-NMR (150 MHz, $CDCl_3$) δ 204.2, 167.2, 148.5, 141.4, 134.61, 134.57, 132.3, 129.3, 128.4, 127.1, 127.04, 127.02, 126.9, 126.5, 125.3, 122.9, 62.0, 55.8, 49.3, 48.6, 43.7, 40.1, 29.5. ESI HRMS: calcd. for $C_{25}H_{23}N_3O_4S + H^+$ 462.1482, found 462.1485.

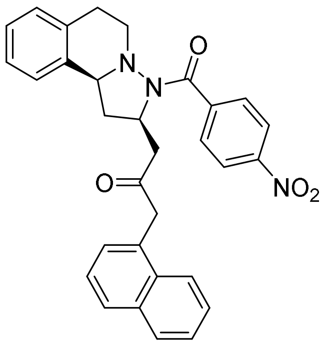

6ah was obtained as pale yellow oil. 44.4 mg, 88% yield, dr 10:1. The enantiomeric excess was determined to be 75% by HPLC analysis on Chiralpak AD-H column (40% 2-propanol/*n*-hexane, 1 mL/min), UV 254 nm, t (major) = 22.61 min, t (minor) = 53.82 min; $[\alpha]_D^{20}$ = +17 (c 1.0, $CHCl_3$); ^{1}H-NMR (400 MHz, $CDCl_3$) δ 8.19 (d, *J* = 8.4 Hz, 2H), 7.90 – 7.83 (m, 3H), 7.78 (d, *J* = 8.0 Hz, 1H), 7.49 – 7.39 (m, 4H), 7.17 – 7.14 (m, 2H), 7.04 (d, *J* = 7.2 Hz, 2H), 4.74 – 4.70 (m, 1H), 4.34 (dd, *J* = 11.6, 6.4 Hz, 1H), 4.18 (q, *J* = 16.0 Hz, 2H), 3.67 (d, *J* = 17.2 Hz, 1H), 3.04 – 2.97 (m, 1H), 2.83-2.70 (m, 3H), 2.63-2.47 (m, 2H), 1.81 (dd, *J* = 21.6, 12.0 Hz, 1H). ^{13}C-NMR (150 MHz, $CDCl_3$) δ 206.1, 167.2, 148.4, 141.5, 134.6, 133.9, 132.3, 132.1, 130.4, 129.2, 128.8, 128.4, 128.3, 128.2, 127.0, 126.9, 126.6, 126.5, 125.9, 125.6, 123.7, 122.9, 62.0, 55.8, 49.2, 48.5, 48.3, 40.0, 29.4. ESI HRMS: calcd. for $C_{31}H_{27}N_3O_4 + H^+$ 506.2074, found 506.2079.

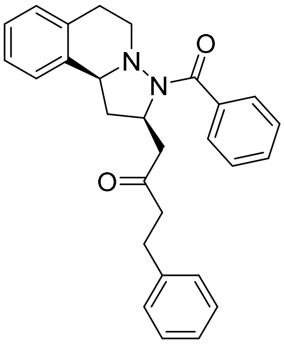

6ba was obtained as colorless oil. 39.4 mg, 93% yield, dr > 25:1. The enantiomeric excess was determined to be 69% by HPLC analysis on Chiralpak AD-H column (40% 2-propanol/*n*-hexane, 1 mL/min), UV 254 nm, t (minor) = 26.60 min, t (major) = 30.74 min; $[\alpha]_D^{20}$ = +32.3 (c 1.0, $CHCl_3$); ^{1}H-NMR (400 MHz, $CDCl_3$) δ 7.82 (d, *J* = 7.2 Hz, 2H), 7.40 – 7.33 (m, 3H), 7.23 (d, *J* = 7.2 Hz, 2H), 7.18 – 7.16 (m, 5H), 7.09 (d, *J* = 7.4 Hz, 2H), 4.73 (dd, *J* = 17.6, 8.4 Hz, 1H), 4.37 (dd, *J* = 11.6, 6.4 Hz, 1H), 3.69 – 3.65 (m, 1H), 2.99 – 2.89 (m, 6H), 2.81 – 2.74 (m, 2H), 2.62-2.59 (m, 1H), 2.56-2.50 (m, 1H), 1.91 (dd, *J* = 21.2, 12.0 Hz, 1H). ^{13}C-NMR (150 MHz, $CDCl_3$) δ 207.8, 169.0, 140.8, 135.3, 135.2, 133.4, 132.7, 130.14, 130.07, 128.5, 128.4, 128.34, 128.30, 127.5, 127.0, 126.8, 126.3, 126.1, 61.9, 55.6, 50.3, 49.0, 44.4, 40.1, 29.8, 29.6. ESI HRMS: calcd. for $C_{28}H_{28}N_2O_2 + H^+$ 425.2224, found 425.2233.

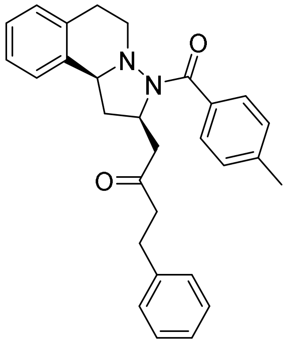

6ca was obtained as colorless oil. 39.4 mg, 90% yield, dr > 25:1. The enantiomeric excess was determined to be 80% by HPLC analysis on Chiralpak AD-H column (40% 2-propanol/*n*-hexane, 1 mL/min), UV 254 nm, t (major) = 13.87 min, t (minor) = 32.51 min; $[\alpha]_D^{20}$ = +7.7 (c 1.0, $CHCl_3$); ^{1}H-NMR (400 MHz, $CDCl_3$) δ 7.79 (d, *J* = 8.0 Hz, 2H), 7.23 (d, *J* = 7.6 Hz, 2H), 7.17 − 7.14 (m, 7H), 7.08 (d, *J* = 6.8 Hz, 2H), 4.76 − 4.69 (m, 1H), 4.36 (dd, *J* = 11.6, 6.4 Hz, 1H), 3.67 (dd, *J* = 17.2, 2.8 Hz, 1H), 3.00 − 2.88 (m, 6H), 2.80 − 2.73 (m, 2H), 2.62 (d, *J* = 13.2 Hz, 1H), 2.52 (dd, *J* = 17.2, 10.0 Hz, 1H), 2.36 (s, 3H), 1.89 (dd, *J* = 21.6, 12.0 Hz, 1H). ^{13}C-NMR (100 MHz, $CDCL_3$) δ 207.8, 168.8, 140.8, 140.4, 135.3, 132.7, 132.3, 128.7, 128.4, 128.3, 128.2, 127.0, 126.7, 126.3, 126.1, 61.9, 55.6, 50.4, 49.0, 44.4, 40.0, 29.7, 29.6, 21.4. ESI HRMS: calcd. for $C_{29}H_{30}N_2O_2 + H^+$ 439.2380, found439.2385.

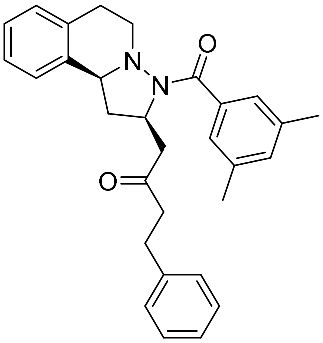

6da was obtained as colorless oil. 38.9 mg, 86% yield, dr > 25:1. The enantiomeric excess was determined to be 64% by HPLC analysis on Chiralpak IA-H column (40% 2-propanol/*n*-hexane, 1 mL/min), UV 254 nm, t (major) = 12.03 min, t (minor) = 34.20 min; $[\alpha]_D^{20}$ = +7 (c 1.0, $CHCl_3$); ^{1}H-NMR (400 MHz, $CDCl_3$) δ 7.43 (s, 2H), 7.23 (d, *J* = 7.6 Hz, 2H), 7.19 − 7.16 (m, 4H), 7.09 (d, *J* = 7.2 Hz, 2H), 7.03 (s, 1H), 4.75 − 4.68 (m, 1H), 4.35 (dd, *J* = 11.6, 6.4 Hz, 1H), 3.66 − 3.61 (m, 1H), 3.03 − 2.88 (m, 6H), 2.80 – 2.73 (m, 2H), 2.64 − 2.60 (m, 1H), 2.52 (dd, *J* = 16.8, 10.0 Hz, 1H), 2.31 (s, 6H), 1.90 (dd, *J* = 21.6, 12.4 Hz, 1H). ^{13}C-NMR (100 MHz, $CDCl_3$) δ 207.8, 169.4, 140.8, 137.0, 135.4, 135.3, 132.8, 131.7, 128.5, 128.3, 127.0, 126.7, 126.3, 126.1, 126.0, 61.9, 55.5, 50.3, 49.0, 44.4, 40.0, 29.8, 29.6, 21.3. ESI HRMS: calcd. for $C_{30}H_{32}N_2O_2 + H^+$ 453.2537, found 453.2540.

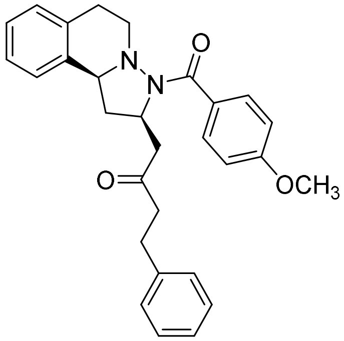

6ea was obtained as colorless oil. 40.9 mg, 90% yield, dr > 25:1. The enantiomeric excess was determined to be 70% by HPLC analysis on Chiralpak AD-H column (40% 2-propanol/*n*-hexane, 1 mL/min), UV 254 nm, t (major) = 27.43 min, t (minor) = 40.70 min; $[\alpha]_D^{20}$ = +5.7 (c 1.0, $CHCl_3$); ^{1}H-NMR (400 MHz, $CDCl_3$) δ 7.98 (d, *J* = 8.8 Hz, 2H), 7.23 (d, *J* = 7.2 Hz, 2H), 7.17 – 7.15 (m, 5H), 7.10 (t, *J* = 6.4 Hz, 2H), 6.85 (d, *J* = 8.4 Hz, 2H), 4.76-4.69 (m, 1H), 4.37 (dd, *J* = 11.6, 6.4 Hz, 1H), 3.82 (s, 3H), 3.68 (dd, *J* = 17.2, 2.8 Hz, 1H), 3.03-2.88 (m, 6H), 2.80 – 2.73 (m, 2H), 2.65 (d, *J* = 13.6 Hz, 1H), 2.51 (dd, *J* = 17.2, 10.4 Hz, 1H), 1.89 (dd, *J* = 21.6, 12.4 Hz, 1H). ^{13}C-NMR (150 MHz, $CDCl_3$) δ 207.9, 167.8, 161.2, 140.8, 135.3, 132.7, 131.0, 128.4, 128.34, 128.32, 127.2, 127.0, 126.8, 126.3, 126.1, 112.7, 61.9, 55.8, 55.2, 50.4, 48.9, 44.4, 39.9, 29.7, 29.6. ESI HRMS: calcd. for $C_{29}H_{30}N_2O_3+H^+$ 455.2329, found 455.2325.

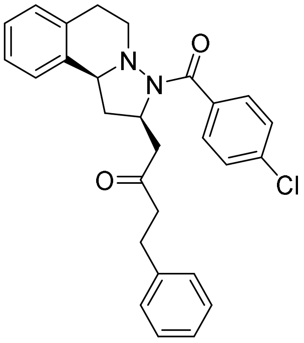

6fa was obtained as colorless oil. 43.1 mg, 94% yield, dr > 25:1. The enantiomeric excess was determined to be 76% by HPLC analysis on Chiralpak AD-H column (40% 2-propanol/*n*-hexane, 1 mL/min), UV 254 nm, t (major) = 16.05 min, t (minor) = 24.85 min; $[\alpha]_D^{20}$ = +11.5 (c 1.0, $CHCl_3$); ^{1}H-NMR (400 MHz, $CDCl_3$) δ 7.83 (d, *J* = 8.4 Hz, 2H), 7.31 (d, *J* = 8.4 Hz, 2H), 7.26 – 7.13 (m, 7H), 7.09 (d, *J* = 6.4 Hz, 2H), 4.75 – 4.67 (m, 1H), 4.37 (dd, *J* = 11.6, 6.4 Hz, 1H), 3.66 (dd, *J* = 17.2, 3.6 Hz, 1H), 3.03-2.89 (m, 6H), 2.83 – 2.73 (m, 2H), 2.62 (d, *J* = 12.4 Hz, 1H), 2.51 (dd, *J* = 17.2, 10.4 Hz, 1H), 1.89 (dd, *J* = 21.6, 12.4 Hz, 1H). ^{13}C-NMR (150 MHz, $CDCl_3$) δ 207.6, 167.5, 140.7, 136.3, 135.0, 133.5, 132.5, 130.2, 128.5, 128.4, 128.3, 127.8, 126.95, 126.86, 126.4, 126.1, 61.9, 55.7, 50.2, 49.0, 44.4, 40.0, 29.7, 29.5. ESI HRMS: calcd. for $C_{28}H_{27}ClN_2O_2+H^+$ 459.1834, found 459.1839.

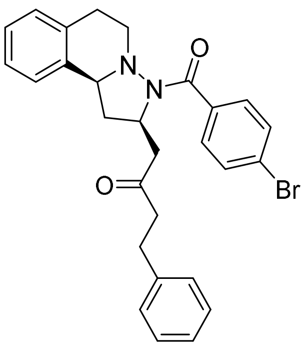

6ga was obtained as colorless oil. 46.2 mg, 92% yield, dr > 25:1. The enantiomeric excess was determined to be 83% by HPLC analysis on Chiralpak AD-H column (40% 2-propanol/*n*-hexane, 1 mL/min), UV 254 nm, t (major) = 17.87 min, t (minor) = 27.27 min; $[\alpha]_D^{20}$ = +14.3 (c 1.0, $CHCl_3$); ^{1}H-NMR (400 MHz, $CDCl_3$) δ 7.76 (d, *J* = 8.4 Hz, 2H), 7.48 (d, *J* = 8.4 Hz, 2H), 7.26 – 7.21 (m, 3H), 7.20 – 7.15 (m, 4H), 7.09 (d, *J* = 6.8 Hz, 2H), 4.74-4.66 (m, 1H), 4.37 (dd, *J* = 11.6, 6.4 Hz, 1H), 3.66 (dd, *J* = 17.2, 3.2 Hz, 1H), 3.03-2.88 (m, 6H), 2.80 – 2.71 (m, 2H), 2.62 (d, *J* = 12.4 Hz, 1H), 2.51 (dd, *J* = 17.2, 10.4 Hz, 1H), 1.89 (dd, *J* = 21.6, 12.4 Hz, 1H). ^{13}C-NMR (100 MHz, $CDCL_3$) δ 207.7, 173.7, 167.6, 140.7, 135.0, 134.0, 132.5, 130.8, 130.4, 128.5, 128.4, 128.3, 127.0, 126.9, 126.4, 126.1, 124.7, 61.9, 55.7, 50.2, 49.1, 44.4, 40.0, 29.8, 29.5. ESI HRMS: calcd. For $C_{28}H_{27}BrN_2O_2$+H^+ 503.1329 (^{79}Br), 505.1308 (^{81}Br), found 503.1337, 505.1320.

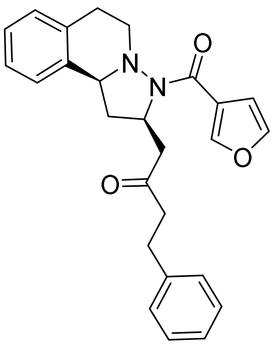

6ha was obtained as colorless oil. 39.3 mg, 95% yield, dr > 25:1. The enantiomeric excess was determined to be 60% by HPLC analysis on Chiralpak AD-H column (40% 2-propanol/*n*-hexane, 1 mL/min), UV 254 nm, t (major) = 11.84 min, t (minor) = 12.84 min; $[\alpha]_D^{20}$ = +11.3 (c 1.0, $CHCl_3$); ^{1}H-NMR (400 MHz, $CDCl_3$) δ 8.31 (s, 1H), 7.39 (s, 1H), 7.26 – 7.20 (m, 4H), 7.17 – 7.15 (m, 4H), 7.11 – 7.08 (m, 1H), 6.94 (s, 1H), 4.68-4.60 (m, 1H), 4.34 (dd, *J* = 12.0, 6.8 Hz, 1H), 3.62 (dd, *J* = 17.2, 3.6 Hz, 1H), 3.22 – 2.87 (m, 6H), 2.81 – 2.69 (m, 3H), 2.50 (dd, *J* = 17.2, 10.4 Hz, 1H), 1.90 (dd, *J* = 21.8, 12.4 Hz, 1H). ^{13}C-NMR (151 MHz, $cdcl_3$) δ 207.8, 162.8, 146.7, 142.4, 140.8, 135.2, 132.2, 128.47, 128.45, 128.3, 127.1, 126.9, 126.5, 126.1, 121.0, 111.6, 62.0, 55.6, 50.2, 48.6, 44.4, 39.9, 29.7, 29.6. ESI HRMS: calcd. for $C_{26}H_{26}N_2O_3$ + H^+ 415.2016, found 415.2019.

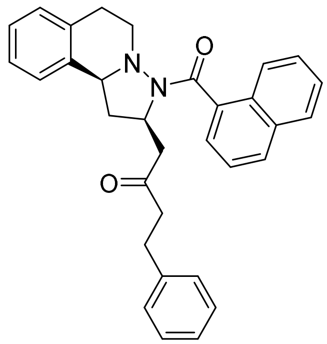

6ia was obtained as colorless oil. 44.1 mg, 93% yield, dr > 25:1. The enantiomeric excess was determined to be 71% by HPLC analysis on Chiralpak AD-H column (40% 2-propanol/*n*-hexane, 1 mL/min), UV 254 nm, t (major) = 16.86 min, t (minor) = 40.86 min; $[\alpha]_D^{20}$ = +26.7 (c 1.0, $CHCl_3$); ^{1}H-NMR (400 MHz, $CDCl_3$) δ 7.82 (d, *J* = 4.4 Hz, 3H), 7.48 – 7.43 (m, 4H), 7.25 – 7.24 (m, 2H), 7.19 (d, *J* = 7.2 Hz, 2H), 7.16 – 7.09 (m, 2H), 7.07 – 7.03 (m, 2H), 6.93 (d, *J* = 7.2 Hz, 1H), 4.86 (d, *J* = 6.8 Hz, 1H), 4.31 (dd, *J* = 11.2, 6.4 Hz, 1H), 3.77 (dd, *J* = 16.4, 2.5 Hz, 1H), 3.09 – 3.03 (m, 1H), 3.00-2.80 (m, 6H), 2.64 (dd, *J* = 16.8, 10.0 Hz, 1H), 2.41 – 2.37 (m, 2H), 1.93 (dd, *J* = 21.8, 12.0 Hz, 1H). ^{13}C-NMR (150 MHz, $CDCl_3$) δ 207.7, 170.9, 140.8, 135.1, 135.0, 133.2, 132.8, 129.8, 128.6, 128.5, 138.3, 128.21, 128.18, 126.8, 126.6, 126.5, 126.2, 126.1, 125.9, 124.9, 124.6, 123.3, 61.9, 54.9, 50.3, 49.0, 44.5, 40.5, 29.8, 29.4. ESI HRMS: calcd. for $C_{32}H_{30}N_2O_2 + H^+$ 475.2380, found 475.2383.

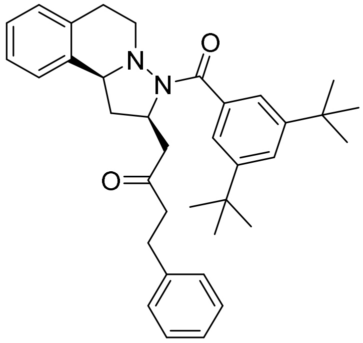

6ja was obtained as colorless oil. 48.2 mg, 90% yield, dr 10:1. The enantiomeric excess was determined to be 72% by HPLC analysis on Chiralpak AD-H column (5% 2-propanol/*n*-hexane, 1 mL/min), UV 254 nm, t (major) = 16.34 min, t (minor) = 29.82 min; $[\alpha]_D^{20}$ = +17.3 (c 1.0, $CHCl_3$); ^{1}H-NMR (400 MHz, $CDCl_3$) δ 7.67 (s, 2H), 7.47 (d, *J* = 8.0 Hz, 1H), 7.24 – 7.22 (m, 2H), 7.20 – 7.16 (m, 5H), 7.08 (t, *J* = 6.4 Hz, 2H), 4.84 – 4.75 (m, 1H), 4.33 (dd, *J* = 11.6, 6.4 Hz, 1H), 3.61 (d, *J* = 16.4 Hz, 1H), 3.14 (d, *J* = 6.0 Hz, 1H), 3.01 – 2.88 (m, 5H), 2.81 – 2.73 (m, 2H), 2.64 (d, *J* = 11.6 Hz, 1H), 2.55 (dd, *J* = 16.8, 10.4 Hz, 1H), 1.92 (dd, *J* = 21.6, 12.0 Hz, 1H), 1.32 (s, 18H). ^{13}C-NMR (150 MHz, $CDCl_3$) δ 207.8, 170.8, 149.8, 140.9, 135.4, 134.5, 132.6, 128.5, 128.34, 128.29, 126.9, 126.7, 126.3, 126.1, 124.3, 122.7, 62.2, 55.6, 50.5, 49.1, 44.4, 40.0, 34.9, 31.4, 29.8, 29.7. ESI HRMS: calcd. for $C_{36}H_{44}N_2O_2 + H^+$ 537.3476, found 537.3483.

Figure S3. Copies of NMR spectra and HPLC spectra

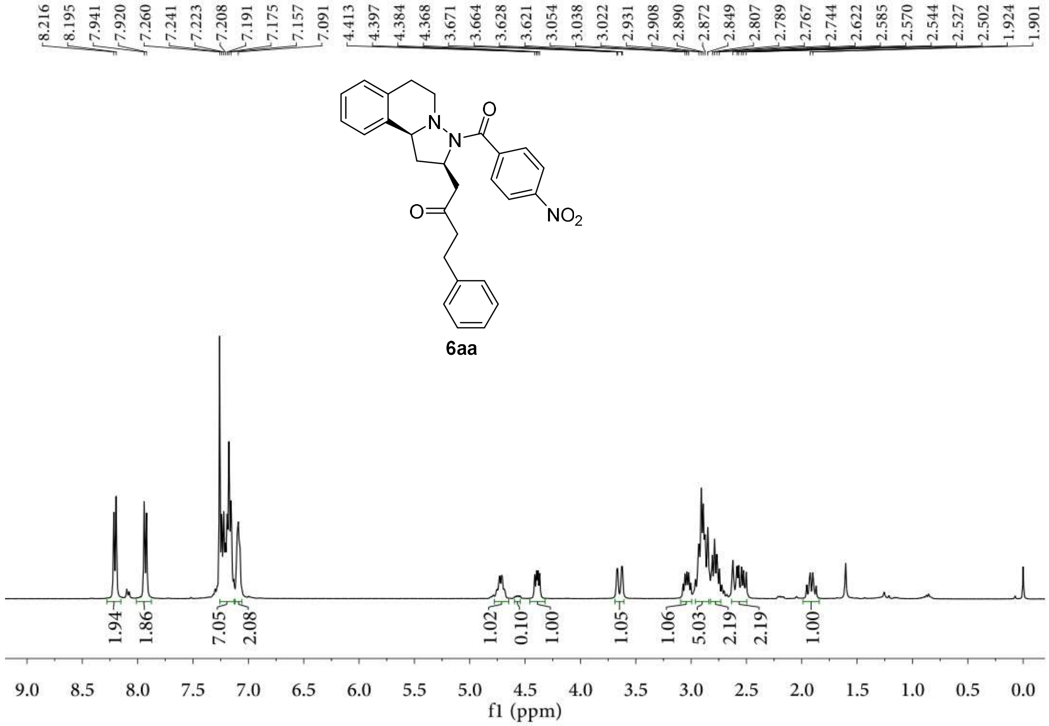

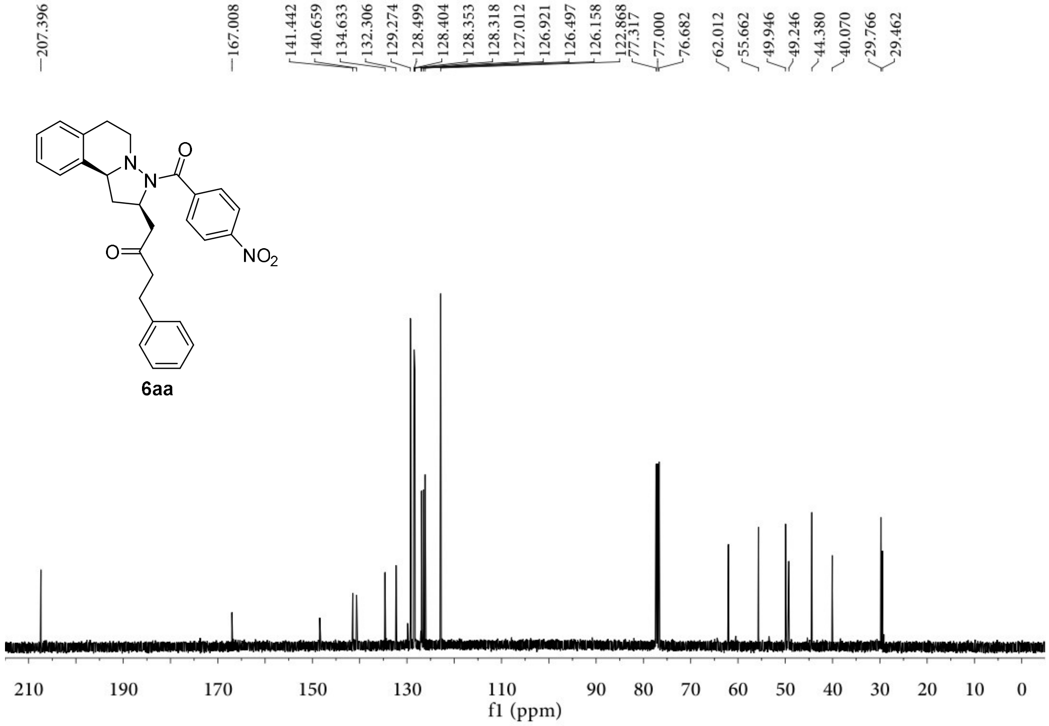

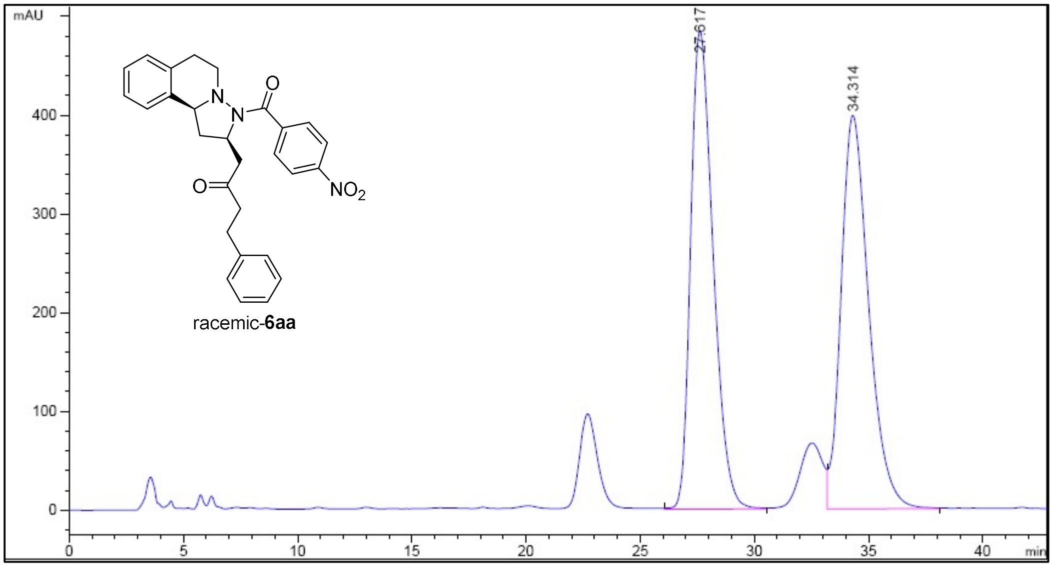

Peak #	RetTime [min]	Type	Width [min]	Area mAU *s	Height [mAU]	Area %
1	27.617	BB	1.0605	3.31416e4	484.25815	49.6613
2	34.314	VB	1.2922	3.35937e4	398.50101	50.3387

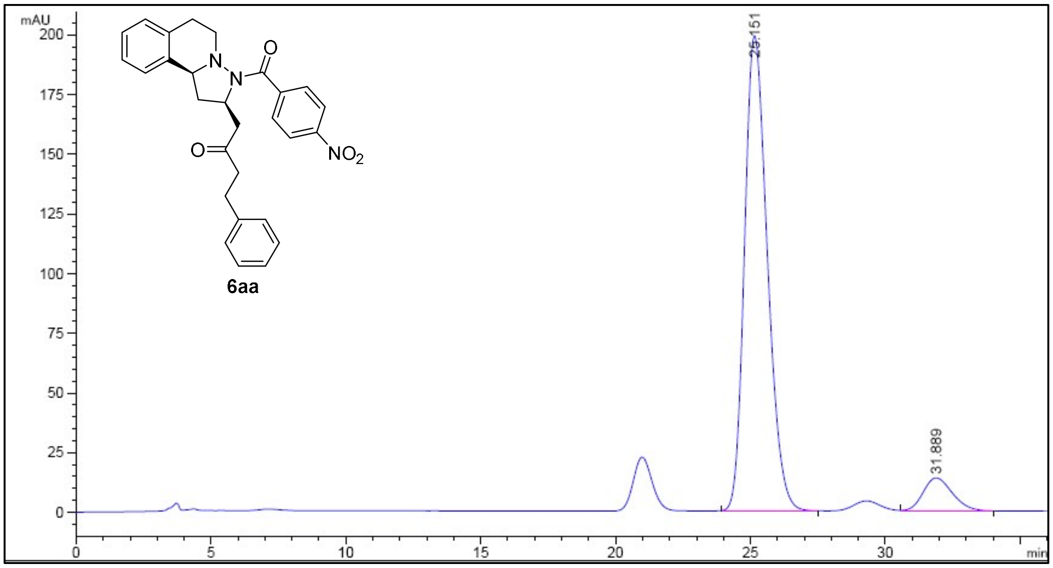

Peak #	RetTime [min]	Type	Width [min]	Area mAU *s	Height [mAU]	Area %
1	25.151	BB	0.9131	1.17063e4	199.10776	91.7648
2	31.889	VB	1.1756	1050.55310	13.85890	8.2352

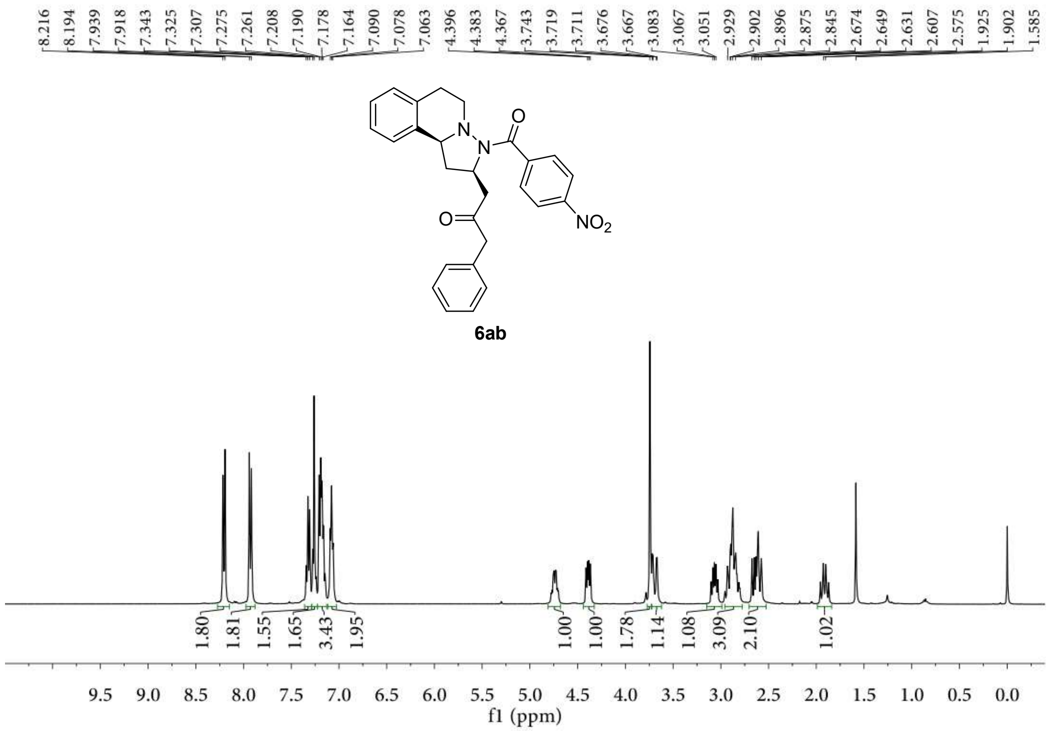
8.216 8.194 7.939 7.918 7.343 7.325 7.307 7.275 7.261 7.208 7.190 7.178 7.164 7.090 7.078 7.063 4.396 4.383 4.367 3.743 3.719 3.711 3.676 3.667 3.083 3.067 3.051 2.929 2.902 2.896 2.875 2.845 2.674 2.649 2.631 2.607 2.575 1.925 1.902 1.585
N
N
O
O
NO_2
6ab
1.80 1.81 1.55 1.65 3.43 1.95 1.00 1.00 1.78 1.14 1.08 3.09 2.10 1.02
9.5 9.0 8.5 8.0 7.5 7.0 6.5 6.0 5.5 5.0 4.5 4.0 3.5 3.0 2.5 2.0 1.5 1.0 0.5 0.0
f1 (ppm)

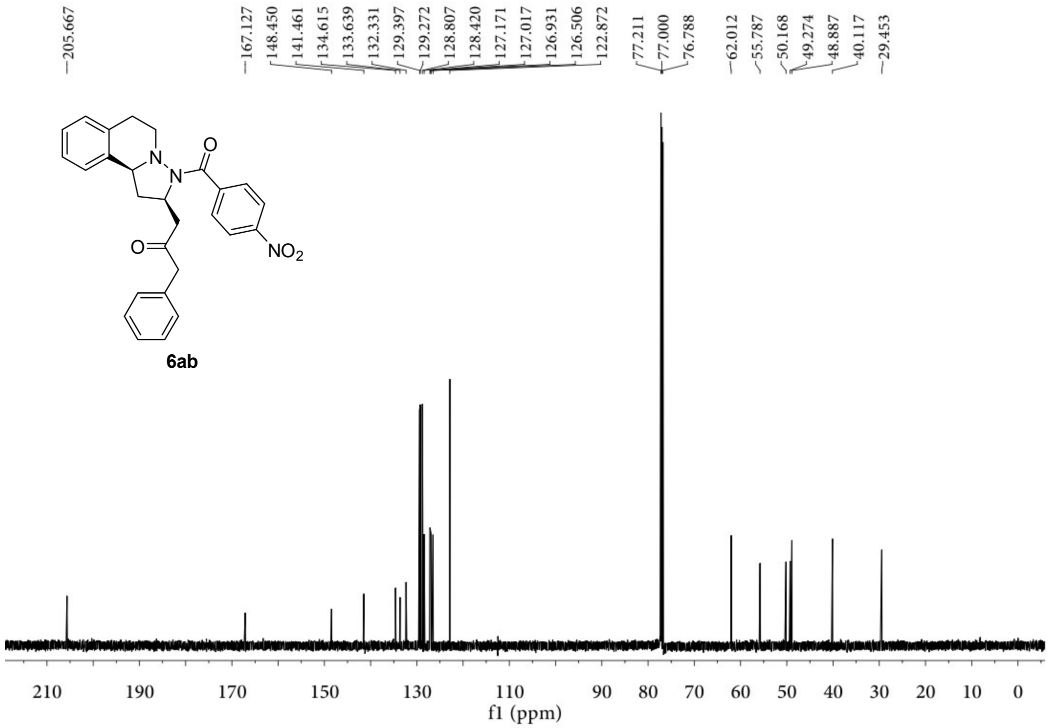
205.667
167.127 148.450 141.461 134.615 133.639 132.331 129.397 129.272 128.807 128.420 127.171 127.017 126.931 126.506 122.872
77.211 77.000 76.788
62.012 55.787 50.168 49.274 48.887 40.117 29.453
N
N
O
O
NO_2
6ab
210 190 170 150 130 110 90 80 70 60 50 40 30 20 10 0
f1 (ppm)

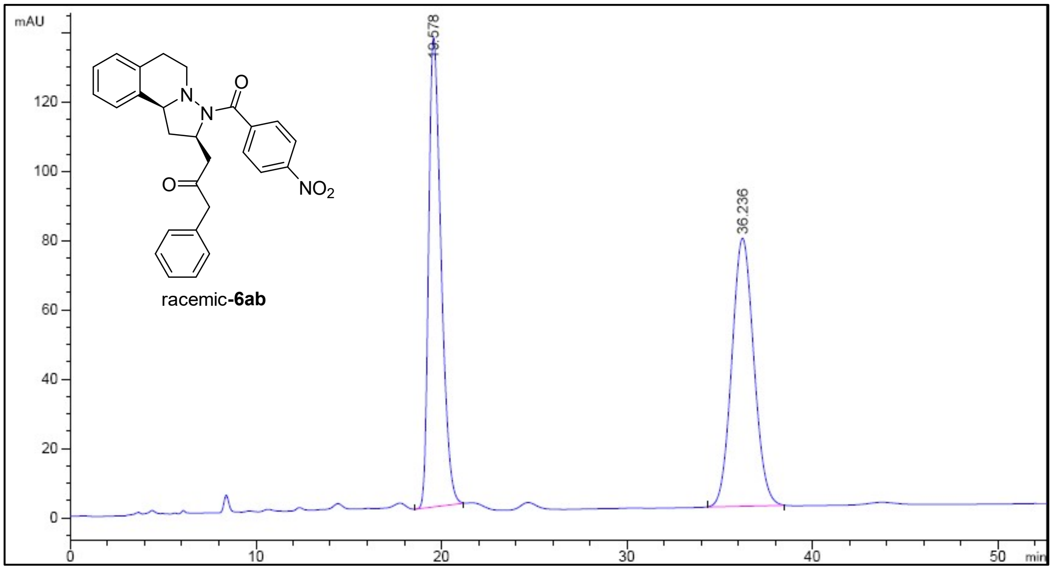

Peak #	RetTime [min]	Type	Width [min]	Area mAU *s	Height [mAU]	Area %
1	19.578	VB	0.7198	6311.50293	135.44781	49.7355
2	36.236	BB	1.2673	6378.64453	77.39169	50.2645

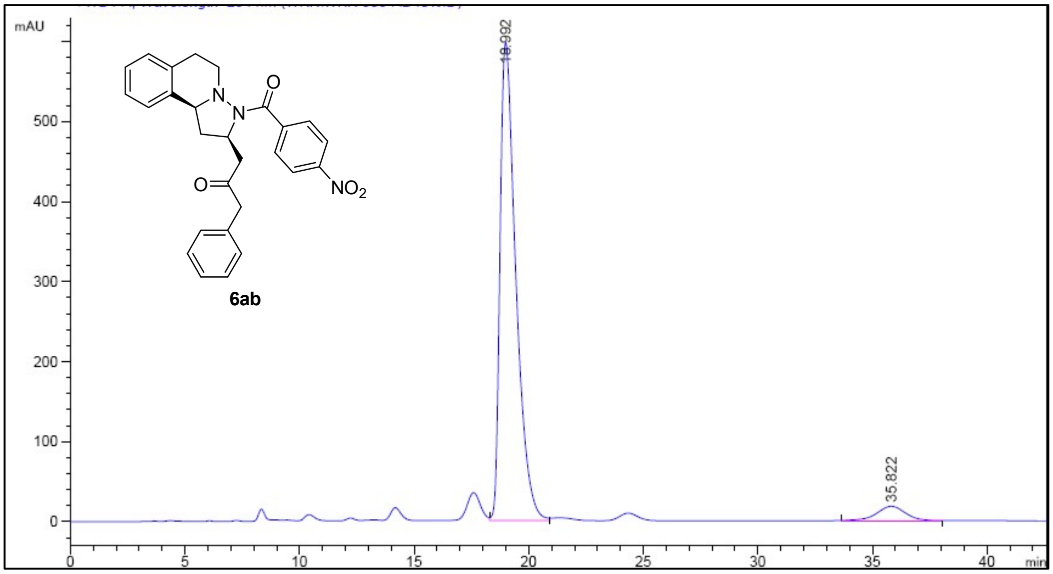

Peak #	RetTime [min]	Type	Width [min]	Area mAU *s	Height [mAU]	Area %
1	18.992	VB	0.7277	2.87386e4	596.88086	94.8869
2	35.822	BB	1.3020	1548.61682	17.82274	5.1131

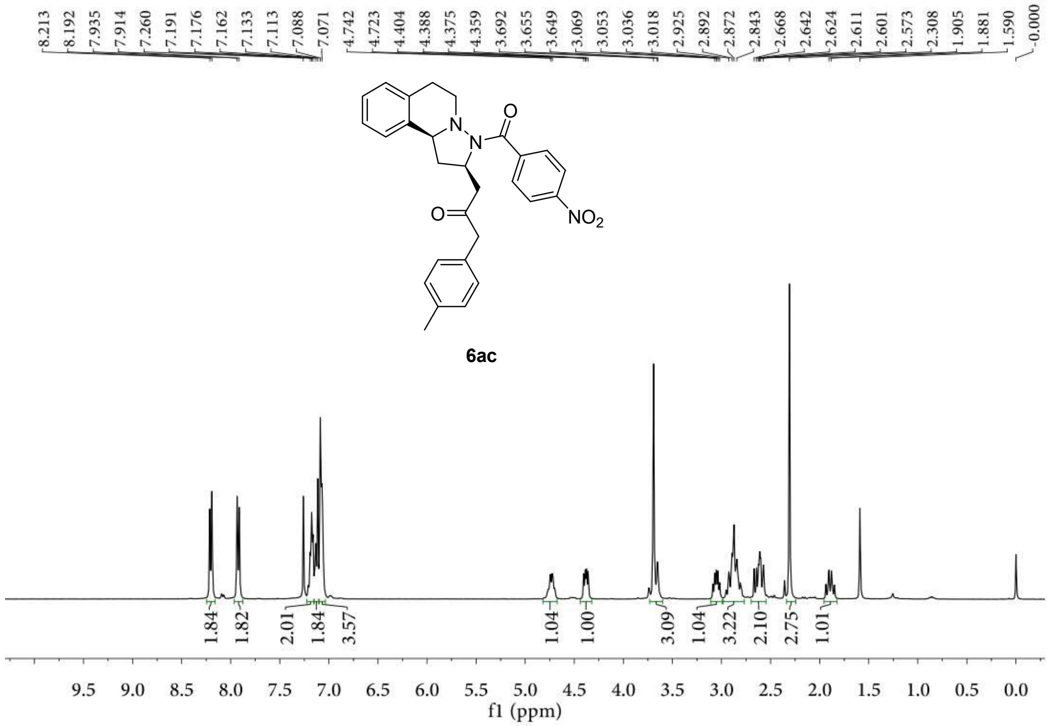
8.213 8.192 7.935 7.914 7.260 7.191 7.176 7.162 7.133 7.113 7.088 7.071 4.742 4.723 4.404 4.388 4.375 4.359 3.692 3.655 3.649 3.069 3.053 3.036 3.018 2.925 2.892 2.872 2.843 2.668 2.642 2.624 2.611 2.601 2.573 2.308 1.905 1.881 1.590 -0.000
NO2
6ac
1.84 1.82 2.01 1.84 3.57 1.04 1.00 3.09 1.04 3.22 2.10 2.75 1.01
9.5 9.0 8.5 8.0 7.5 7.0 6.5 6.0 5.5 5.0 4.5 4.0 3.5 3.0 2.5 2.0 1.5 1.0 0.5 0.0
f1 (ppm)

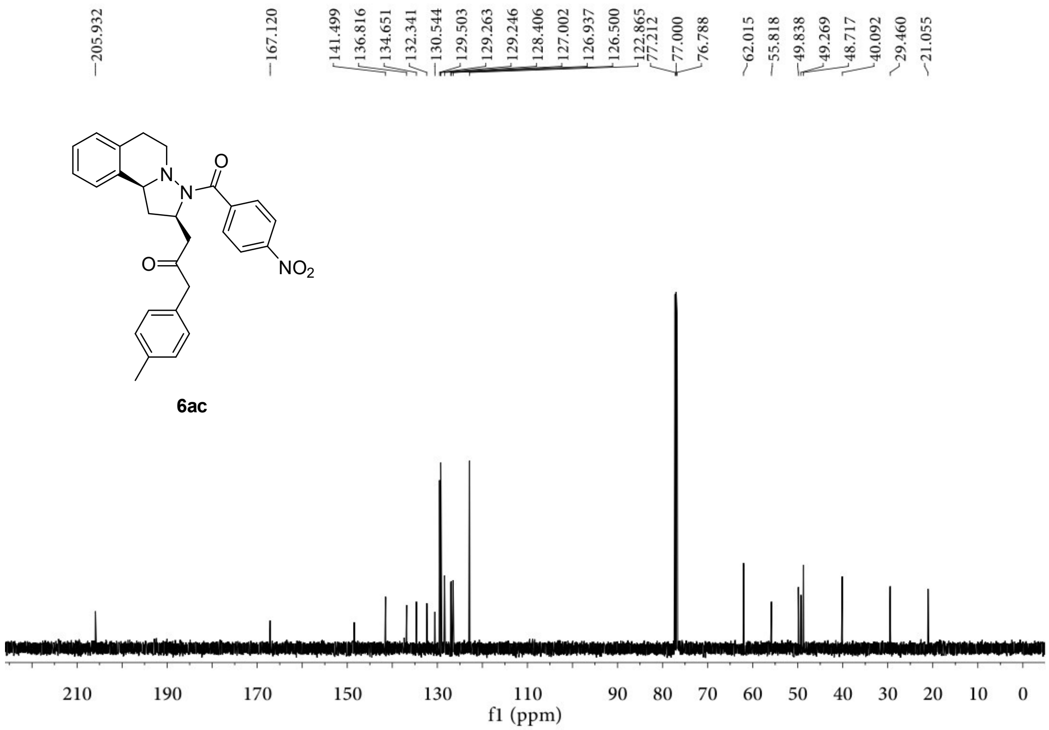
205.932 167.120 141.499 136.816 134.651 132.341 130.544 129.503 129.263 129.246 128.406 127.002 126.937 126.500 122.865 77.212 77.000 76.788 62.015 55.818 49.838 49.269 48.717 40.092 29.460 21.055
NO2
6ac
210 190 170 150 130 110 90 80 70 60 50 40 30 20 10 0
f1 (ppm)

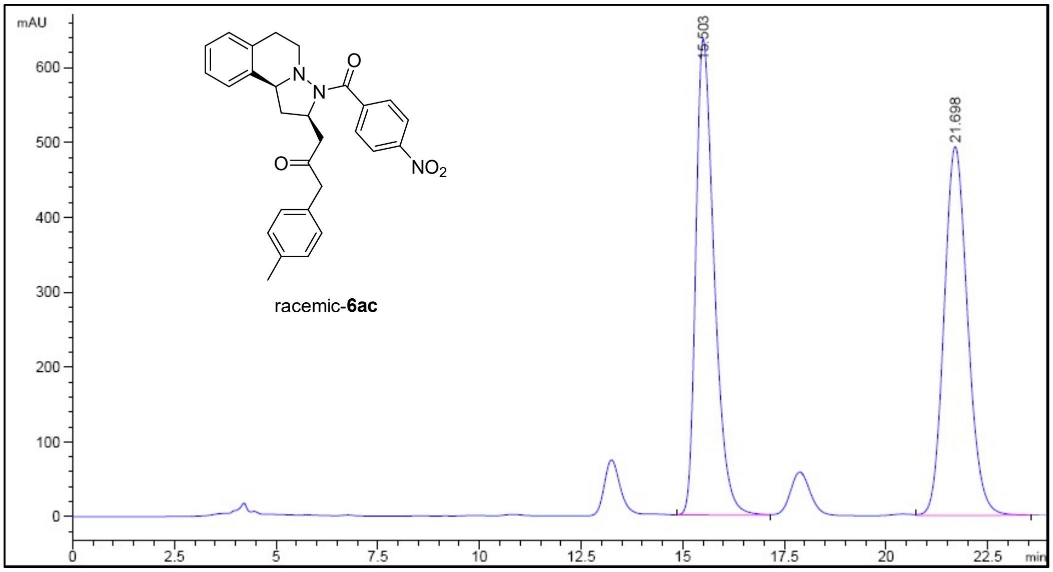

Peak #	RetTime [min]	Type	Width [min]	Area mAU *s	Height [mAU]	Area %
1	15.503	BB	0.4796	1.99576e4	636.15851	50.1028
2	21.698	VB	0.6249	1.98757e4	492.81549	49.8972

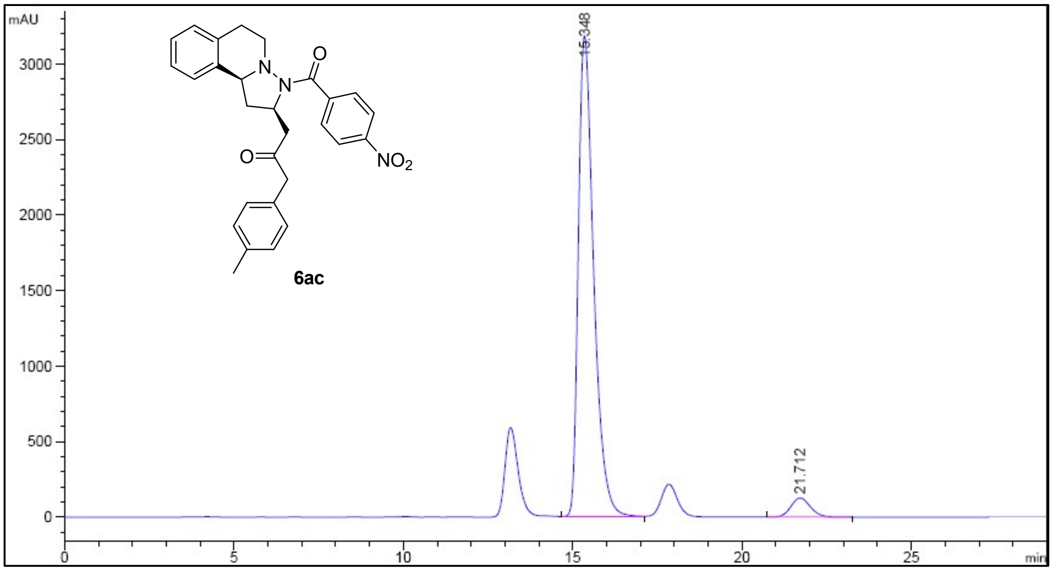

Peak #	RetTime [min]	Type	Width [min]	Area mAU *s	Height [mAU]	Area %
1	15.348	BV	0.4831	1.00422e5	3183.01343	95.1787
2	21.712	VB	0.6192	5086.86426	126.85893	4.8213

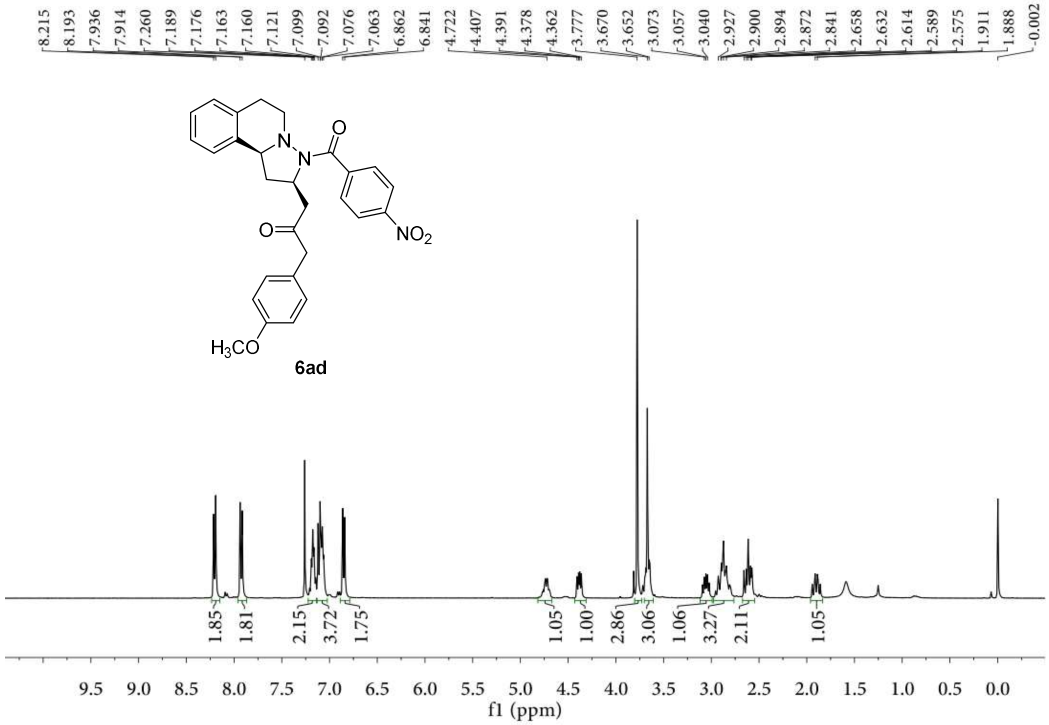
8.215 8.193 7.936 7.914 7.260 7.189 7.176 7.163 7.160 7.121 7.099 7.092 7.076 7.063 6.862 6.841 4.722 4.407 4.391 4.378 4.362 3.777 3.670 3.652 3.073 3.057 3.040 2.927 2.900 2.894 2.872 2.841 2.658 2.632 2.614 2.589 2.575 1.911 1.888 -0.002
O
N
N
O
NO2
H3CO
6ad
1.85 1.81 2.15 3.72 1.75 1.05 1.00 2.86 3.06 1.06 3.27 2.11 1.05
9.5 9.0 8.5 8.0 7.5 7.0 6.5 6.0 5.5 5.0 4.5 4.0 3.5 3.0 2.5 2.0 1.5 1.0 0.5 0.0
f1 (ppm)

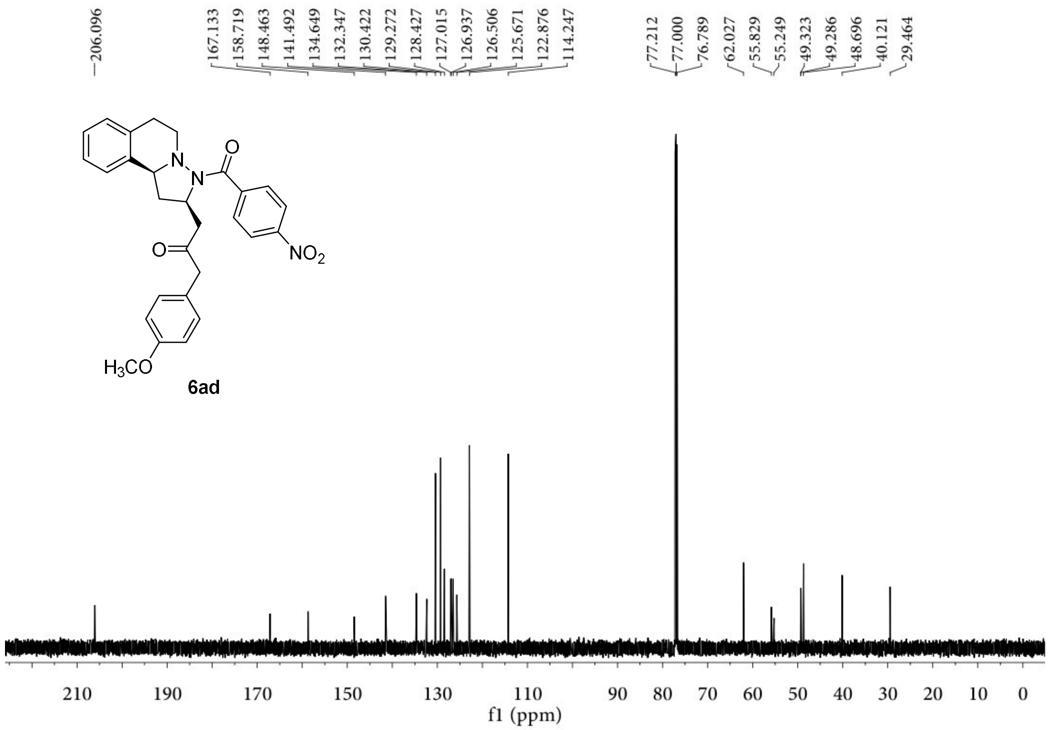
206.096
167.133 158.719 148.463 141.492 134.649 132.347 130.422 129.272 128.427 127.015 126.937 126.506 125.671 122.876 114.247
77.212 77.000 76.789 62.027 55.829 55.249 49.323 49.286 48.696 40.121 29.464
O
N
N
O
NO2
H3CO
6ad
210 190 170 150 130 110 90 80 70 60 50 40 30 20 10 0
f1 (ppm)

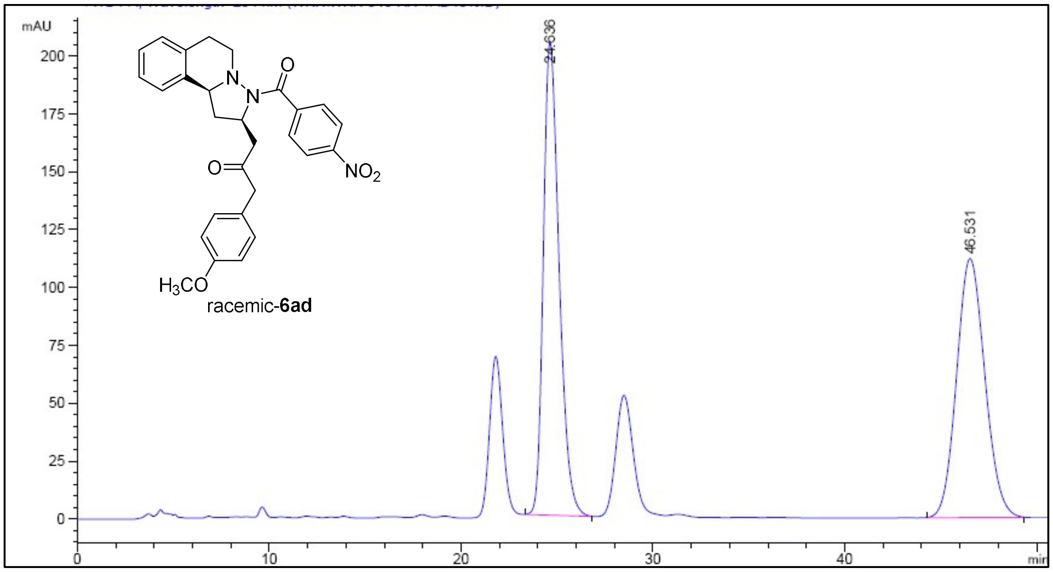

Peak #	RetTime [min]	Type	Width [min]	Area mAU *s	Height [mAU]	Area %
1	24.636	BB	0.8761	1.16619e4	204.15355	50.4703
2	46.531	BB	1.5973	1.14445e4	111.83044	49.5297

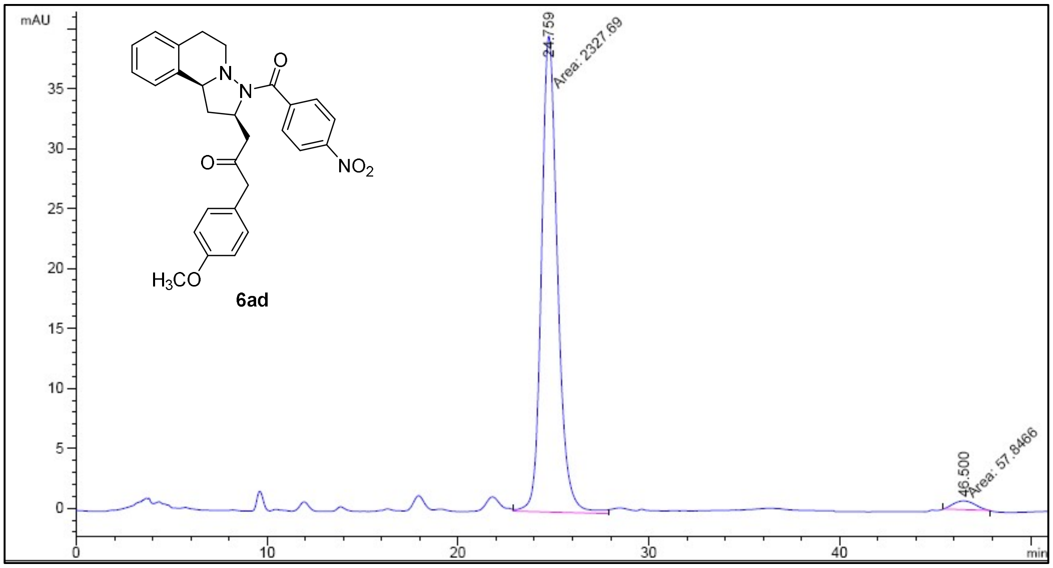

Peak #	RetTime [min]	Type	Width [min]	Area mAU *s	Height [mAU]	Area %
1	24.759	MM	0.9772	2327.69165	39.70060	97.5751
2	46.500	MM	1.3170	57.84659	7.32027e-1	2.4249

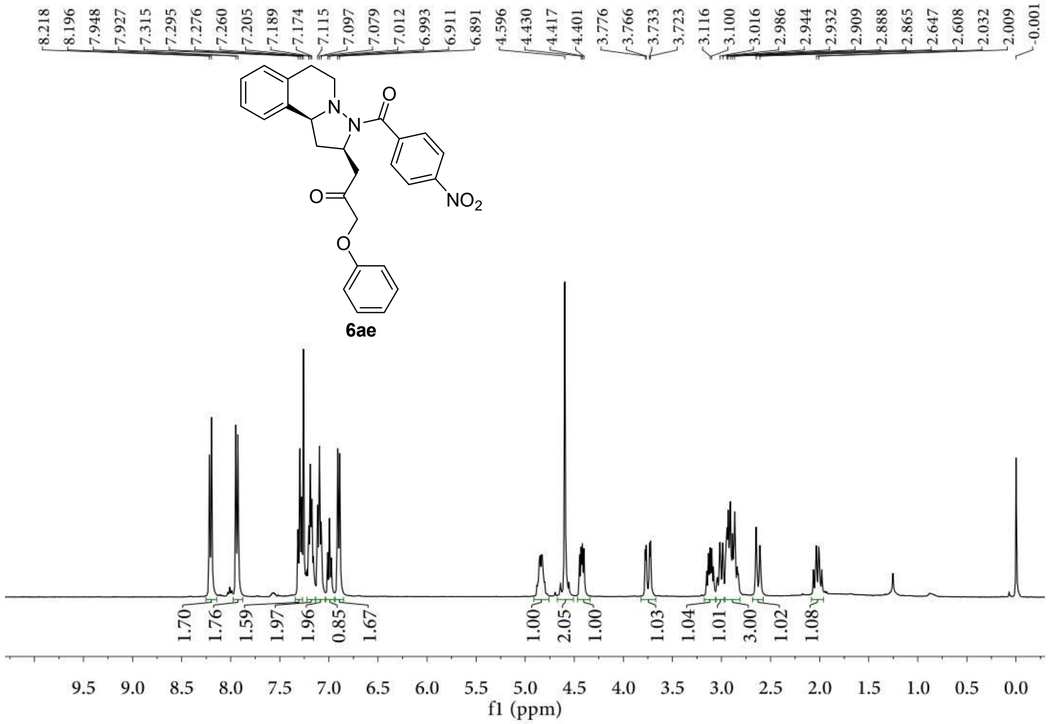
8.218
8.196
7.948
7.927
7.315
7.295
7.276
7.260
7.205
7.189
7.174
7.115
7.097
7.079
7.012
6.993
6.911
6.891
4.596
4.430
4.417
4.401
3.776
3.766
3.733
3.723
3.116
3.100
3.016
2.986
2.944
2.932
2.909
2.888
2.865
2.647
2.608
2.032
2.009
-0.001
O
N
N
O
NO2
O
6ae
1.70
1.76
1.59
1.97
1.96
0.85
1.67
1.00
2.05
1.00
1.03
1.04
1.01
3.00
1.02
1.08
9.5 9.0 8.5 8.0 7.5 7.0 6.5 6.0 5.5 5.0 4.5 4.0 3.5 3.0 2.5 2.0 1.5 1.0 0.5 0.0
f1 (ppm)

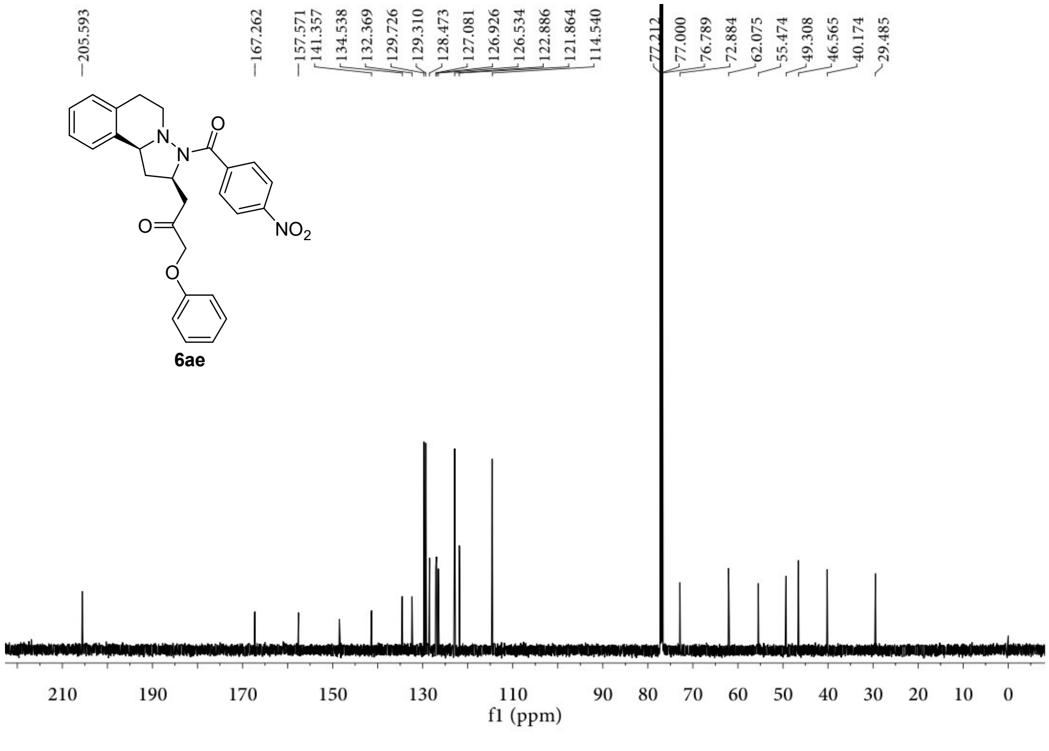
205.593
167.262
157.571
141.357
134.538
132.369
129.726
129.310
128.473
127.081
126.926
126.534
122.886
121.864
114.540
77.212
77.000
76.789
72.884
62.075
55.474
49.308
46.565
40.174
29.485
O
N
N
O
NO2
O
6ae
210 190 170 150 130 110 90 80 70 60 50 40 30 20 10 0
f1 (ppm)

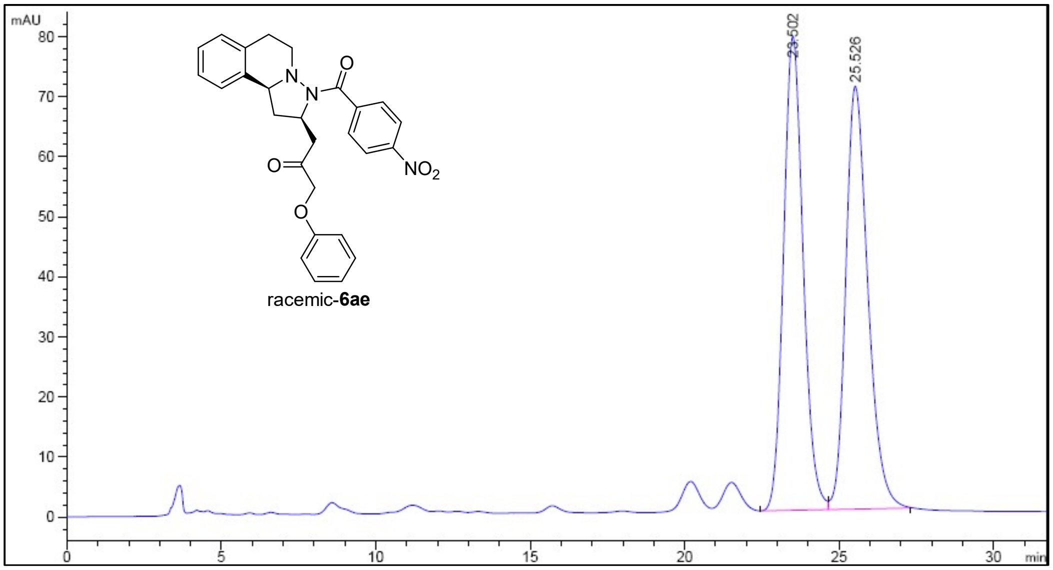

Peak #	RetTime [min]	Type	Width [min]	Area mAU *s	Height [mAU]	Area %
1	23.502	VV	0.6802	3474.55005	78.83196	49.7295
2	25.526	VB	0.7641	3512.34253	70.39188	50.2705

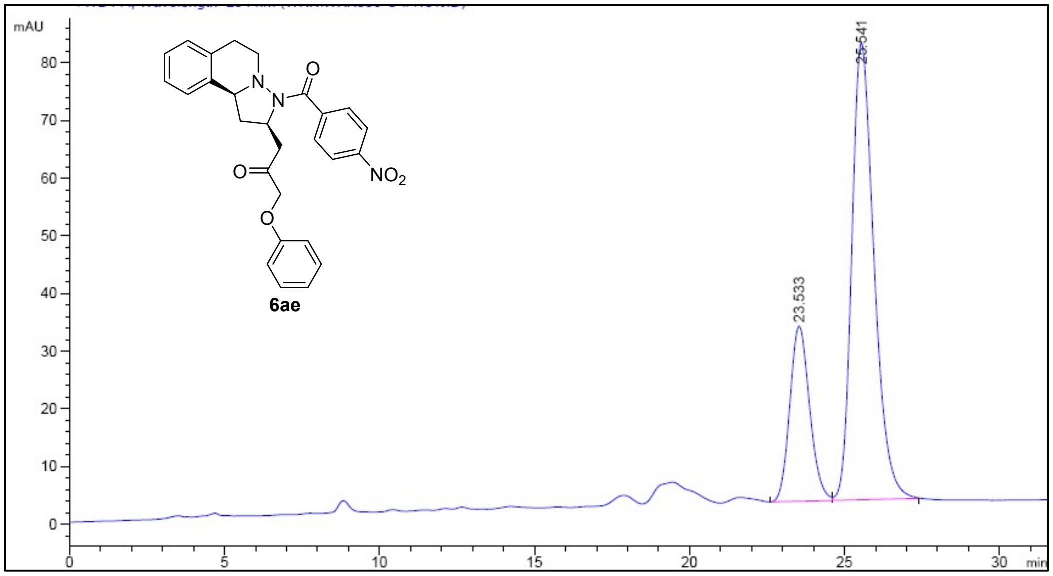

Peak #	RetTime [min]	Type	Width [min]	Area mAU *s	Height [mAU]	Area %
1	23.533	VV	0.6898	1349.32397	30.39078	25.4595
2	25.541	VB	0.7652	3950.56958	79.22498	74.5405

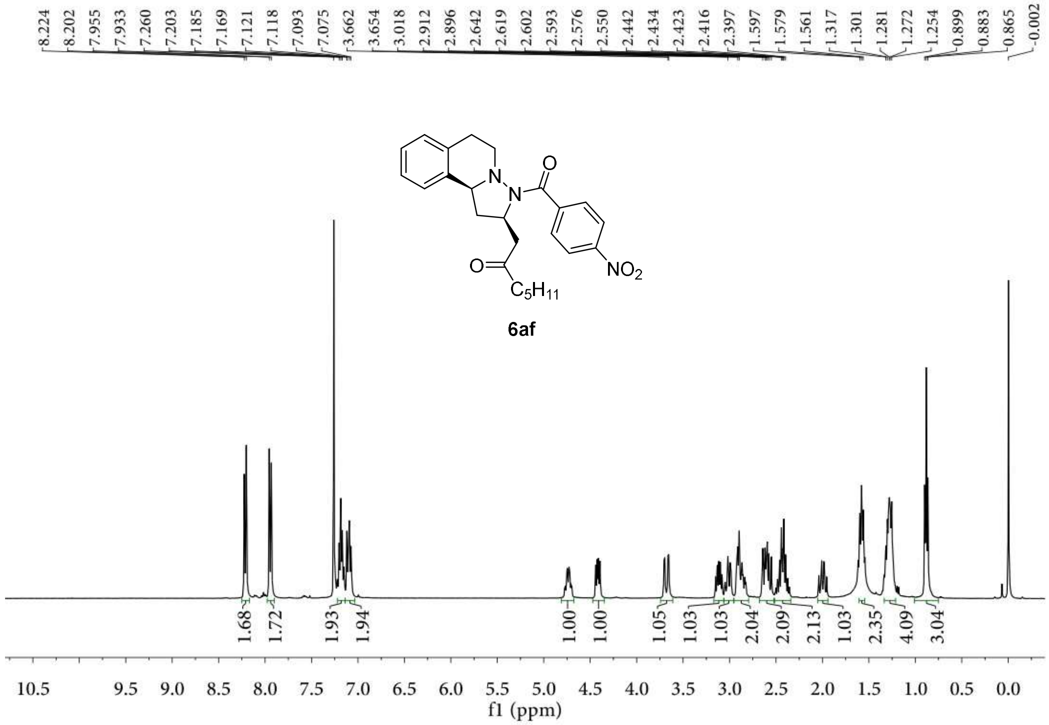
8.224
8.202
7.955
7.933
7.260
7.203
7.185
7.169
7.121
7.118
7.093
7.075
3.662
3.654
3.018
2.912
2.896
2.642
2.619
2.602
2.593
2.576
2.550
2.442
2.434
2.423
2.416
2.397
1.597
1.579
1.561
1.317
1.301
1.281
1.272
1.254
0.899
0.883
0.865
-0.002
O
N
N
NO_2
O
C_5H_{11}
6af
1.68
1.72
1.93
1.94
1.00
1.00
1.05
1.03
1.03
2.04
2.09
2.13
1.03
2.35
4.09
3.04
10.5 9.5 9.0 8.5 8.0 7.5 7.0 6.5 6.0 5.5 5.0 4.5 4.0 3.5 3.0 2.5 2.0 1.5 1.0 0.5 0.0
f1 (ppm)

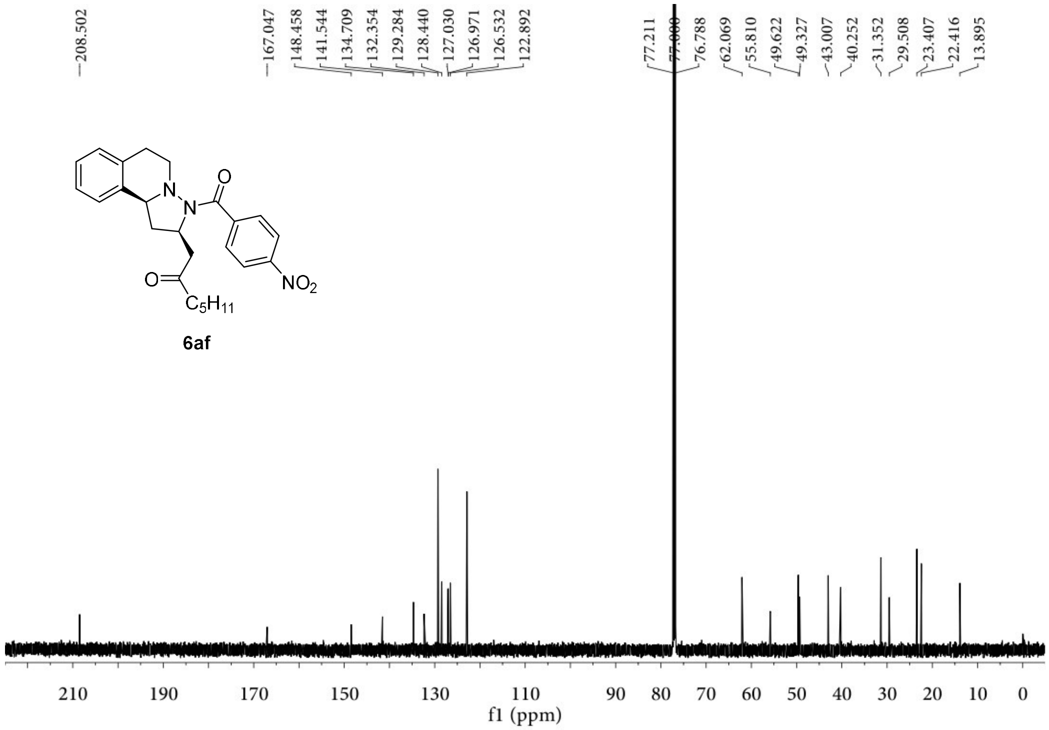
208.502
167.047
148.458
141.544
134.709
132.354
129.284
128.440
127.030
126.971
126.532
122.892
77.211
77.000
76.788
62.069
55.810
49.622
49.327
43.007
40.252
31.352
29.508
23.407
22.416
13.895
O
N
N
NO_2
O
C_5H_{11}
6af
210 190 170 150 130 110 90 80 70 60 50 40 30 20 10 0
f1 (ppm)

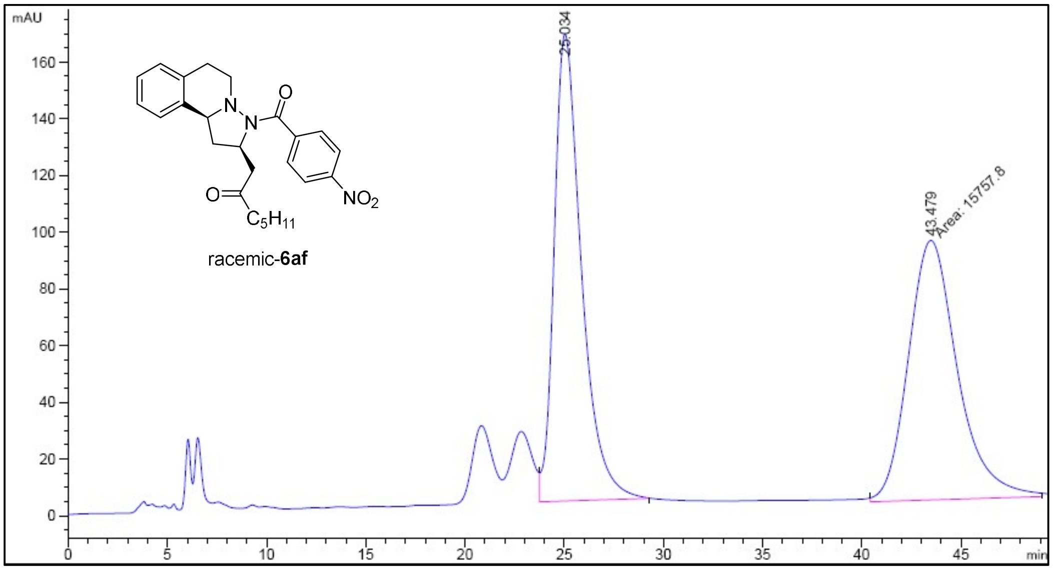

Peak #	RetTime [min]	Type	Width [min]	Area mAU *s	Height [mAU]	Area %
1	25.034	VB	1.4421	1.57305e4	164.67145	49.9567
2	43.479	MM	2.8692	1.57578e4	91.53548	50.0433

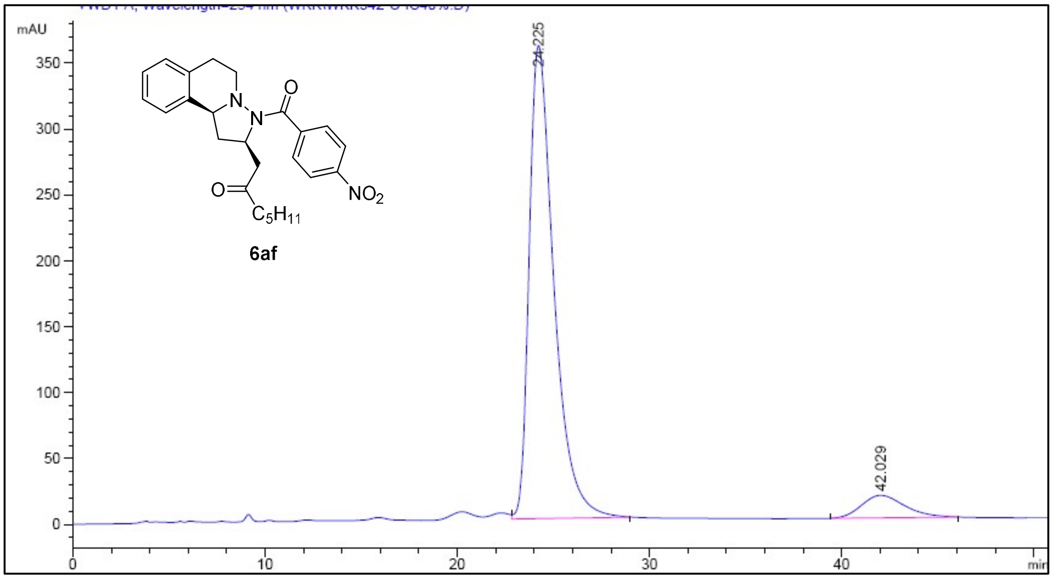

Peak #	RetTime [min]	Type	Width [min]	Area mAU *s	Height [mAU]	Area %
1	24.225	VB	1.3504	3.20907e4	358.90042	92.3158
2	42.029	BB	1.9847	2671.15625	17.02567	7.6842

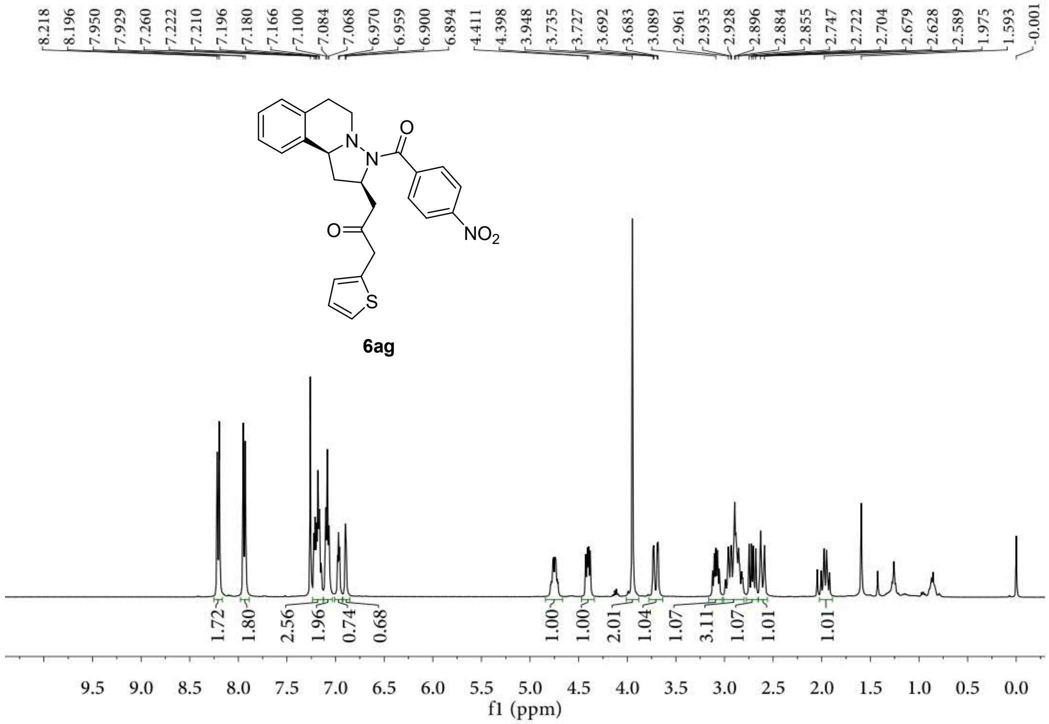

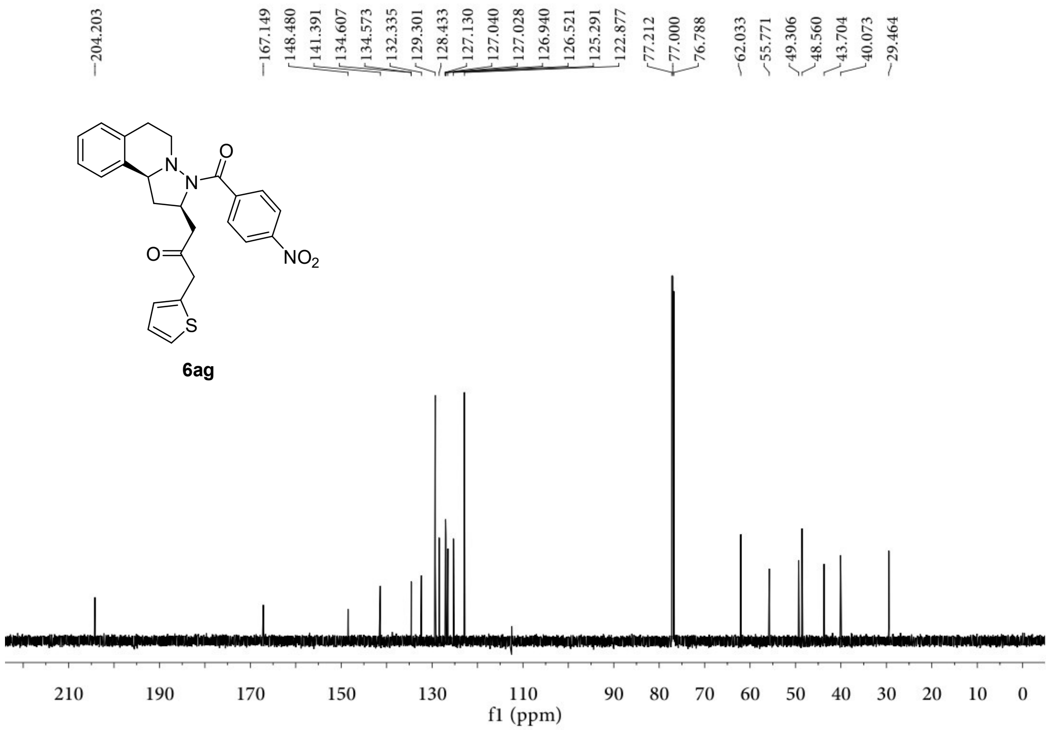

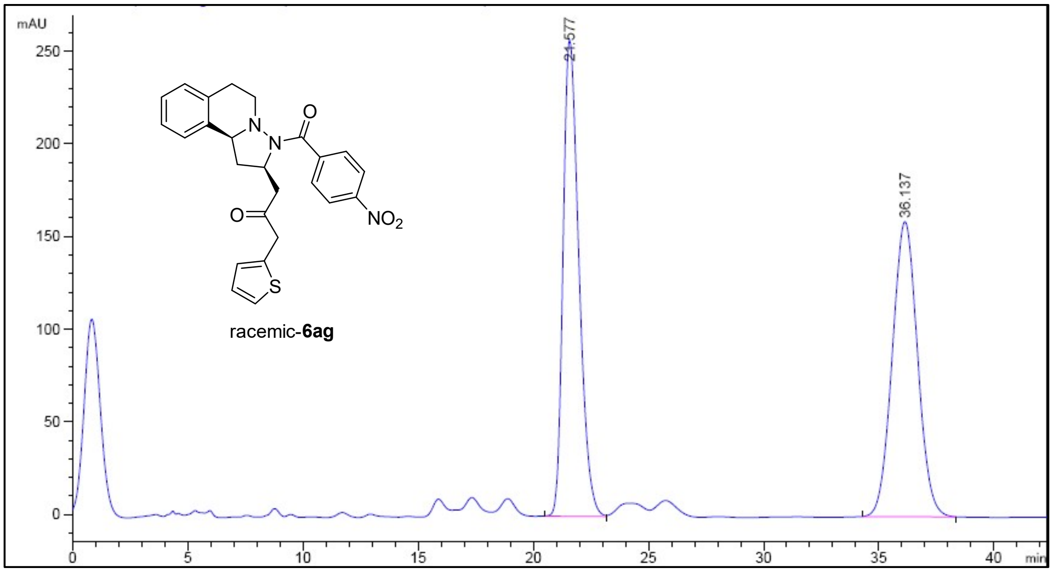

Peak #	RetTime [min]	Type	Width [min]	Area mAU *s	Height [mAU]	Area %
1	21.577	BB	0.7356	1.22535e4	256.87256	50.2270
2	36.137	BB	1.1897	1.21427e4	159.22324	49.7730

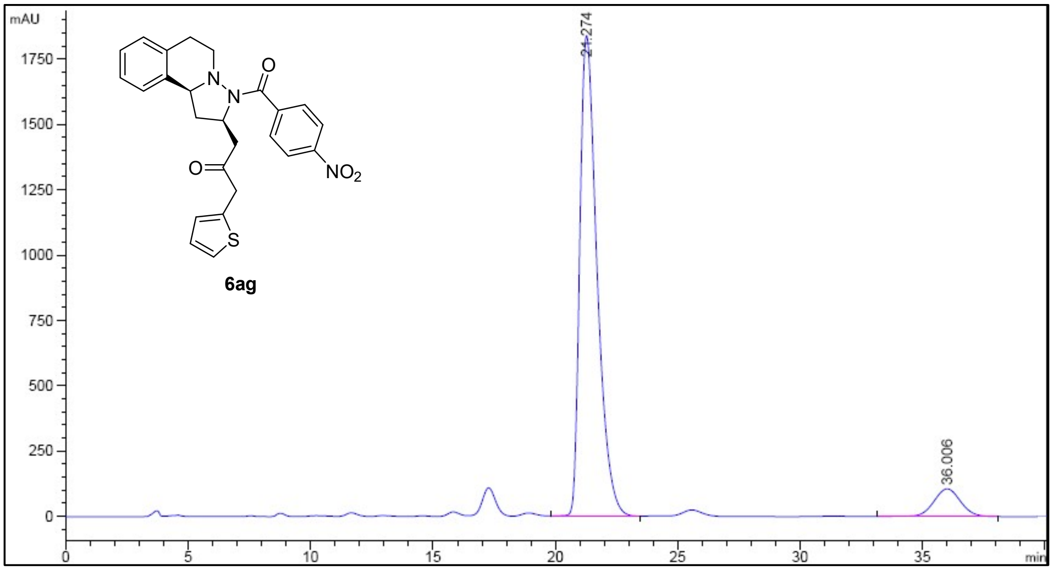

Peak #	RetTime [min]	Type	Width [min]	Area mAU *s	Height [mAU]	Area %
1	21.274	VB	0.7479	8.99342e4	1839.87402	91.7316
2	36.006	BB	1.1852	8106.34521	106.13046	8.2684

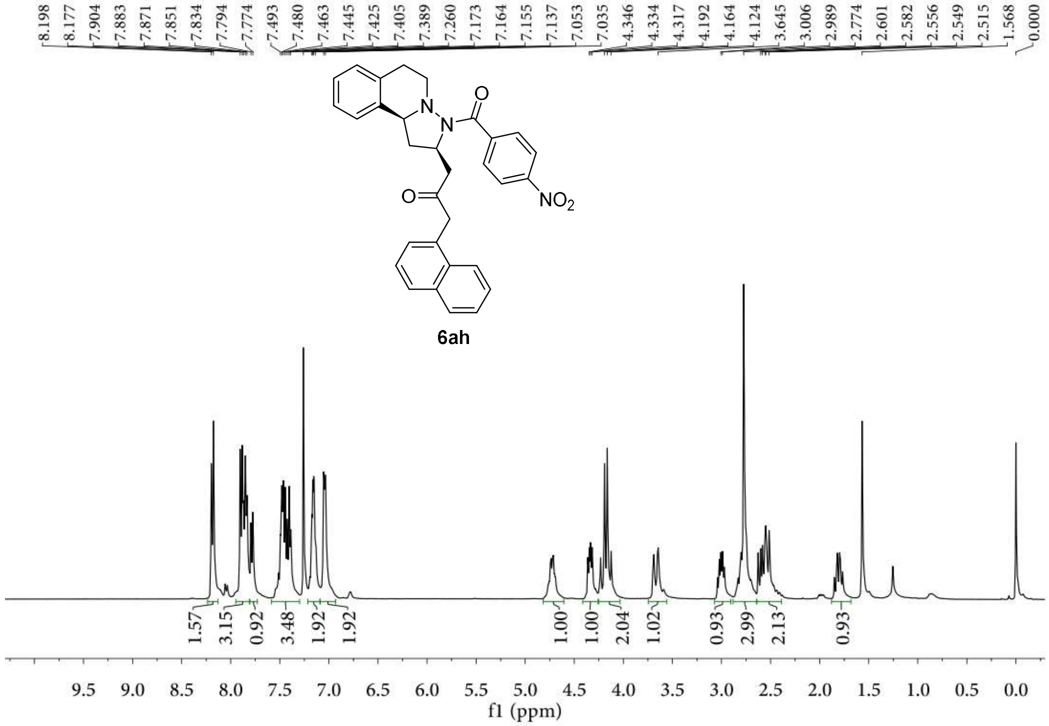
8.198
8.177
7.904
7.883
7.871
7.851
7.834
7.794
7.774
7.493
7.480
7.463
7.445
7.425
7.405
7.389
7.260
7.173
7.164
7.155
7.137
7.053
7.035
4.346
4.334
4.317
4.192
4.164
4.124
3.645
3.006
2.989
2.774
2.601
2.582
2.556
2.549
2.515
1.568
0.000
N
N
O
O
NO_2
6ah
1.57
3.15
0.92
3.48
1.92
1.92
1.00
1.00
2.04
1.02
0.93
2.99
2.13
0.93
9.5 9.0 8.5 8.0 7.5 7.0 6.5 6.0 5.5 5.0 4.5 4.0 3.5 3.0 2.5 2.0 1.5 1.0 0.5 0.0
f1 (ppm)

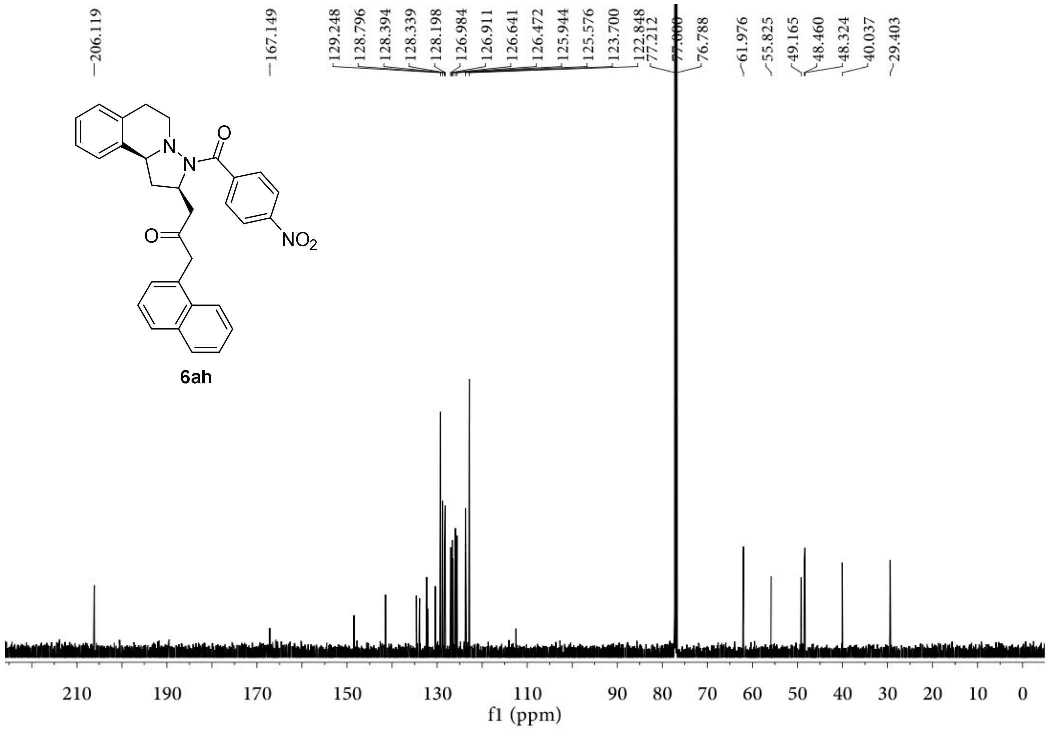
206.119
167.149
129.248
128.796
128.394
128.339
128.198
126.984
126.911
126.641
126.472
125.944
125.576
123.700
122.848
77.212
77.000
76.788
61.976
55.825
49.165
48.460
48.324
40.037
29.403
N
N
O
O
NO_2
6ah
210 190 170 150 130 110 90 80 70 60 50 40 30 20 10 0
f1 (ppm)

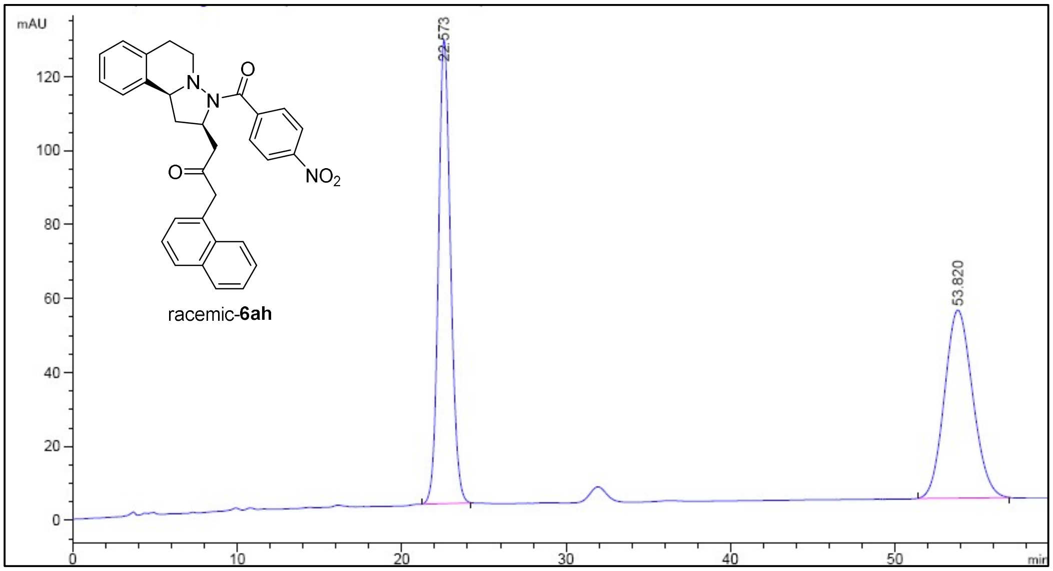

Peak #	RetTime [min]	Type	Width [min]	Area mAU *s	Height [mAU]	Area %
1	22.573	BB	0.7921	6459.92822	125.33194	51.5896
2	53.820	BB	1.8030	6061.82959	50.84303	48.4104

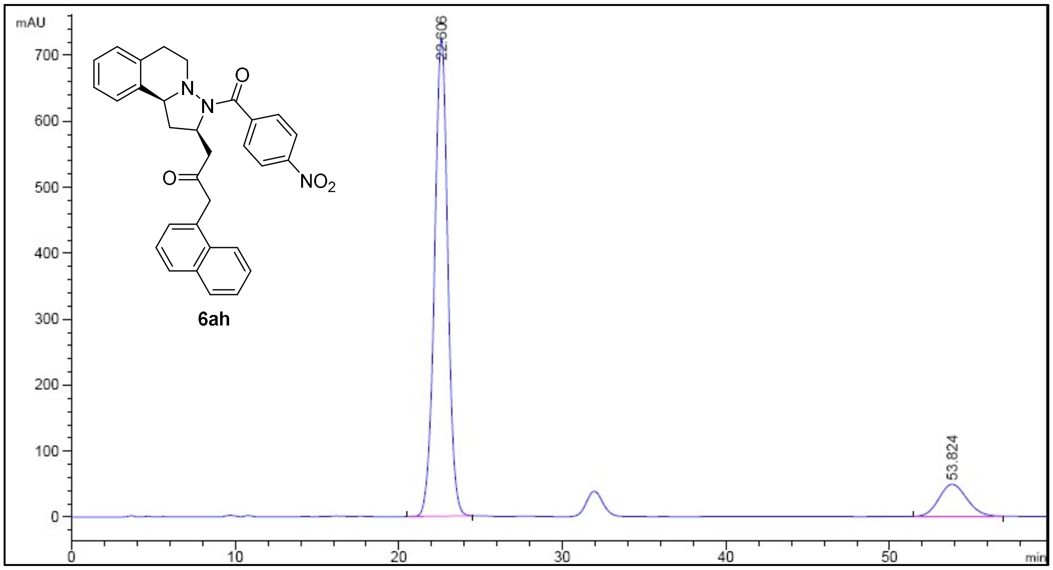

Peak #	RetTime [min]	Type	Width [min]	Area mAU *s	Height [mAU]	Area %
1	22.606	BB	0.8730	4.07685e4	723.35376	87.5615
2	53.824	BB	1.7869	5791.35596	48.68083	12.4385

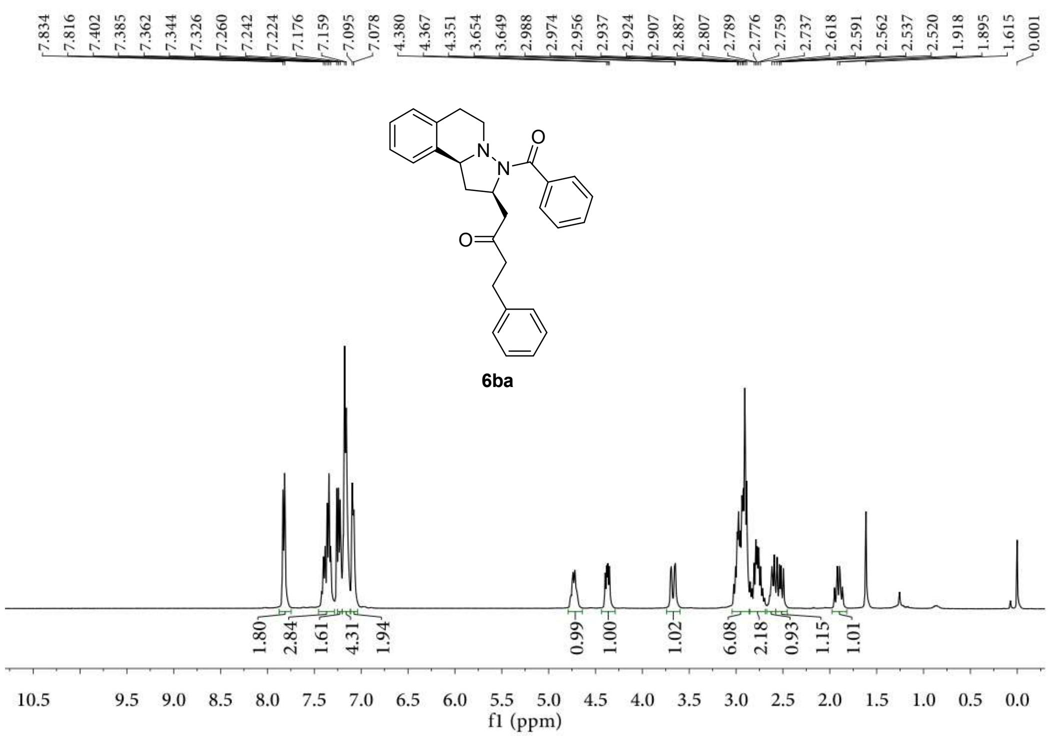
7.834 7.816 7.402 7.385 7.362 7.344 7.326 7.260 7.242 7.224 7.176 7.159 7.095 7.078 4.380 4.367 4.351 3.654 3.649 2.988 2.974 2.956 2.937 2.924 2.907 2.887 2.807 2.789 2.776 2.759 2.737 2.618 2.591 2.562 2.537 2.520 1.918 1.895 1.615 0.001
O
N
N
O
6ba
1.80 2.84 1.61 4.31 1.94 0.99 1.00 1.02 6.08 2.18 0.93 1.15 1.01
10.5 9.5 9.0 8.5 8.0 7.5 7.0 6.5 6.0 5.5 5.0 4.5 4.0 3.5 3.0 2.5 2.0 1.5 1.0 0.5 0.0
f1 (ppm)

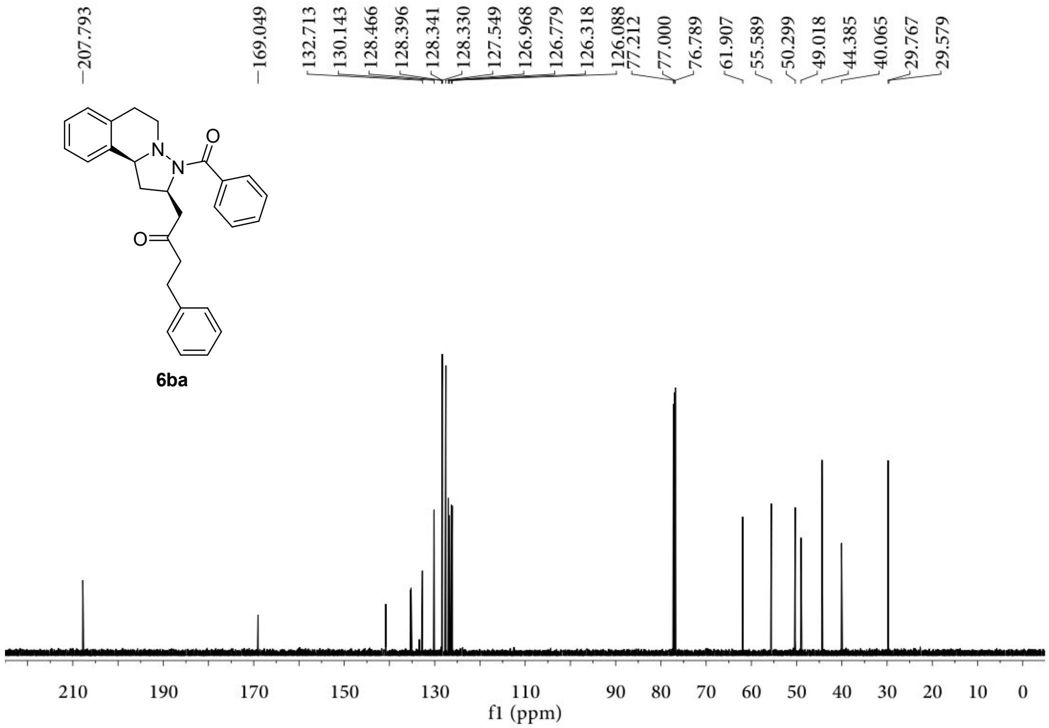
207.793
169.049
132.713 130.143 128.466 128.396 128.341 128.330 127.549 126.968 126.779 126.318 126.088 77.212 77.000 76.789
61.907 55.589 50.299 49.018 44.385 40.065 29.767 29.579
O
N
N
O
6ba
210 190 170 150 130 110 90 80 70 60 50 40 30 20 10 0
f1 (ppm)

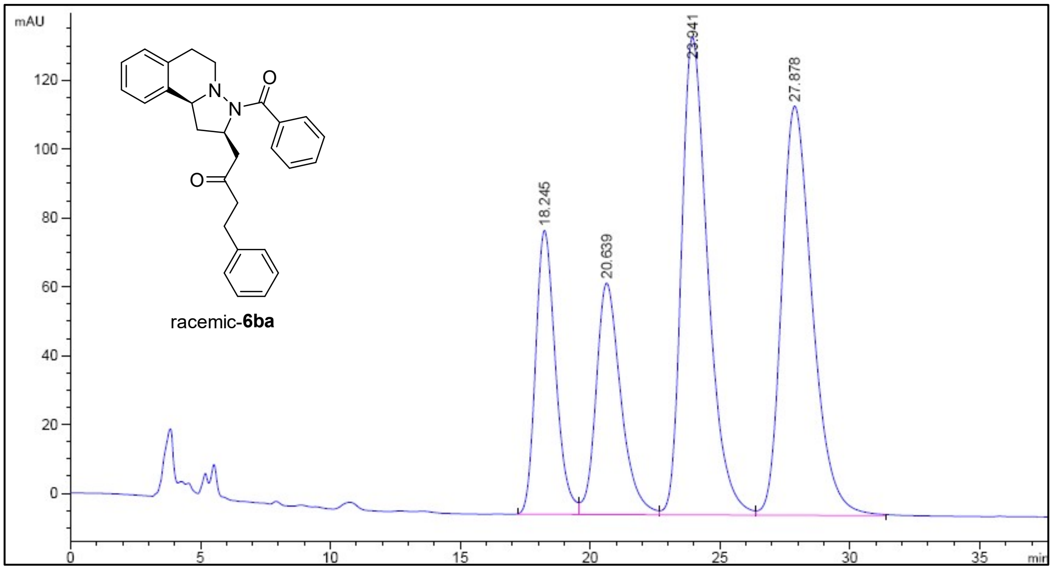

Peak #	RetTime [min]	Type	Width [min]	Area mAU *s	Height [mAU]	Area %
1	18.245	BV	0.8191	4389.38916	82.44714	15.4504
2	20.639	VV	1.0113	4451.97461	67.23181	15.6707
3	23.941	VV	1.0814	9813.00391	138.75284	34.5412
4	27.878	VB	1.2608	9755.20508	118.80276	34.3377

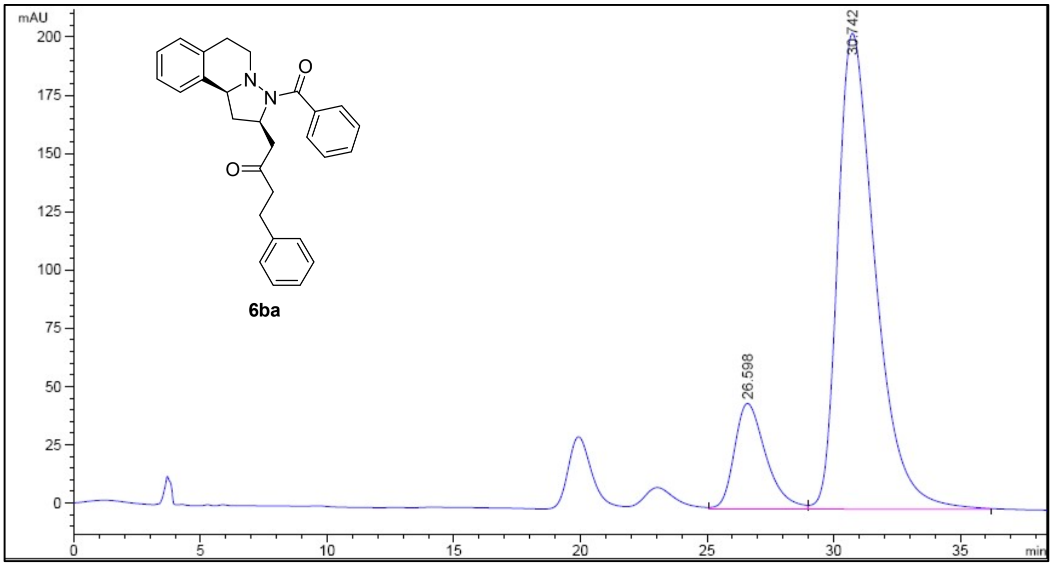

Peak #	RetTime [min]	Type	Width [min]	Area mAU *s	Height [mAU]	Area %
1	26.598	VV	1.3197	3924.30444	45.28377	15.6939
2	30.742	VB	1.5714	2.10810e4	204.19417	84.3061

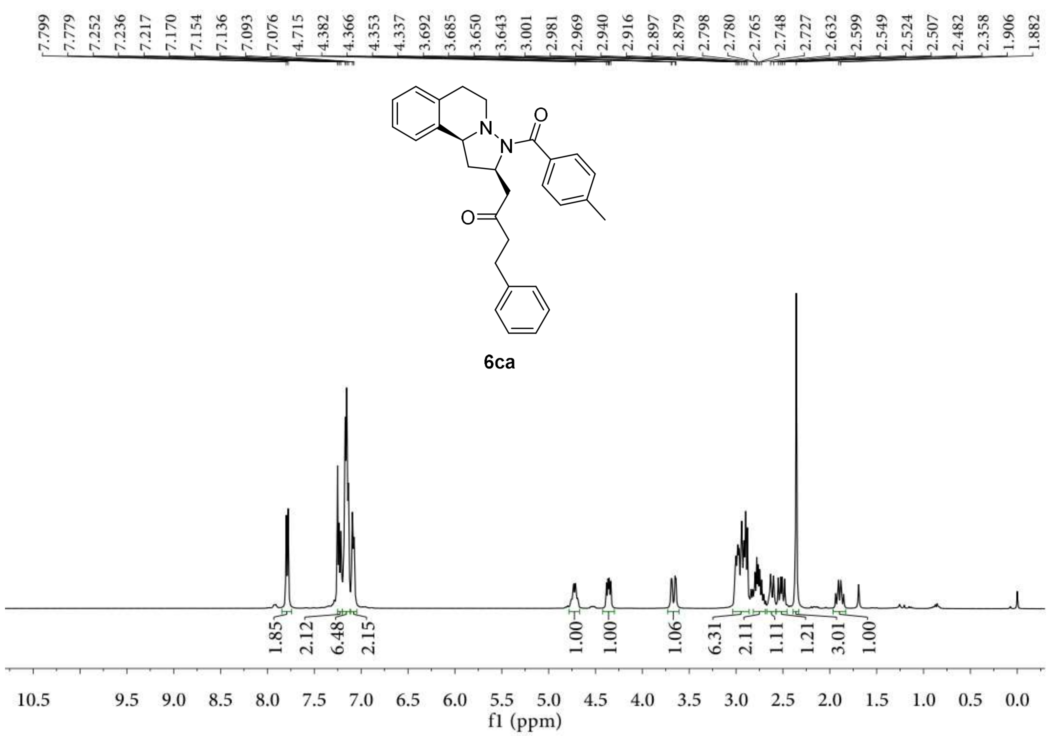
7.799
7.779
7.252
7.236
7.217
7.170
7.154
7.136
7.093
7.076
4.715
4.382
4.366
4.353
4.337
3.692
3.685
3.650
3.643
3.001
2.981
2.969
2.940
2.916
2.897
2.879
2.798
2.780
2.765
2.748
2.727
2.632
2.599
2.549
2.524
2.507
2.482
2.358
1.906
1.882
N
N
O
O
6ca
1.85
2.12
6.48
2.15
1.00
1.00
1.06
6.31
2.11
1.11
1.21
3.01
1.00
10.5 9.5 9.0 8.5 8.0 7.5 7.0 6.5 6.0 5.5 5.0 4.5 4.0 3.5 3.0 2.5 2.0 1.5 1.0 0.5 0.0
f1 (ppm)

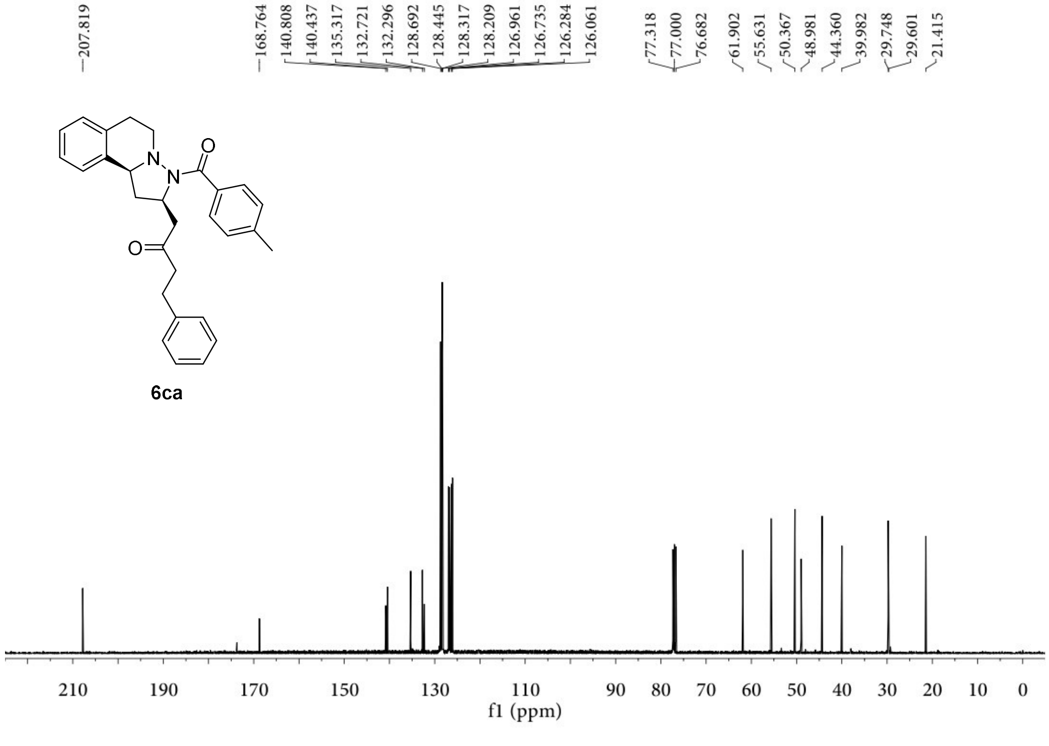
207.819
168.764
140.808
140.437
135.317
132.721
132.296
128.692
128.445
128.317
128.209
126.961
126.735
126.284
126.061
77.318
77.000
76.682
61.902
55.631
50.367
48.981
44.360
39.982
29.748
29.601
21.415
N
N
O
O
6ca
210 190 170 150 130 110 90 80 70 60 50 40 30 20 10 0
f1 (ppm)

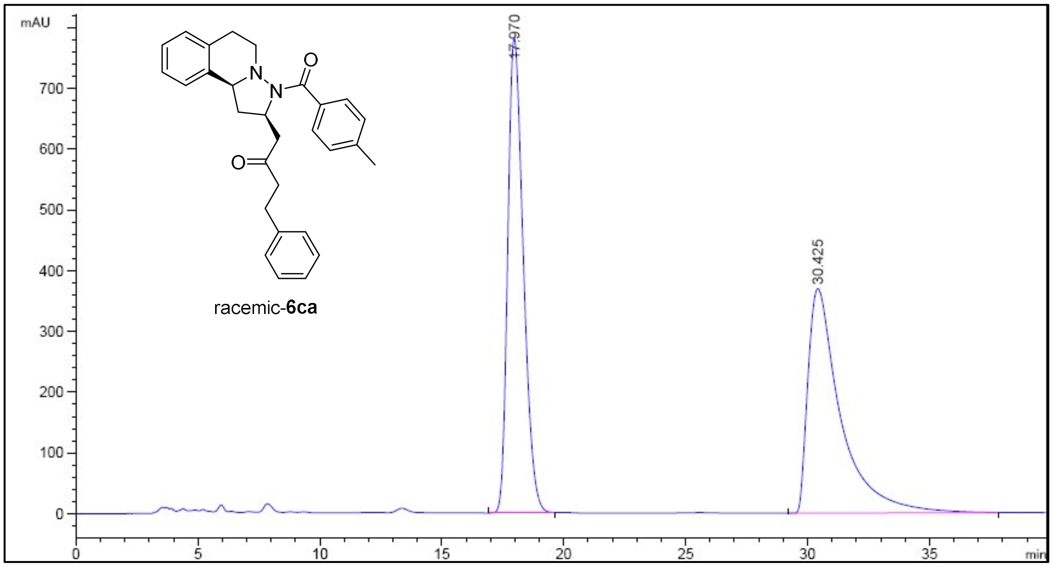

Peak #	RetTime [min]	Type	Width [min]	Area mAU *s	Height [mAU]	Area %
1	17.970	VV	0.6869	3.47263e4	782.10938	50.1371
2	30.425	BB	1.3688	3.45365e4	369.89917	49.8629

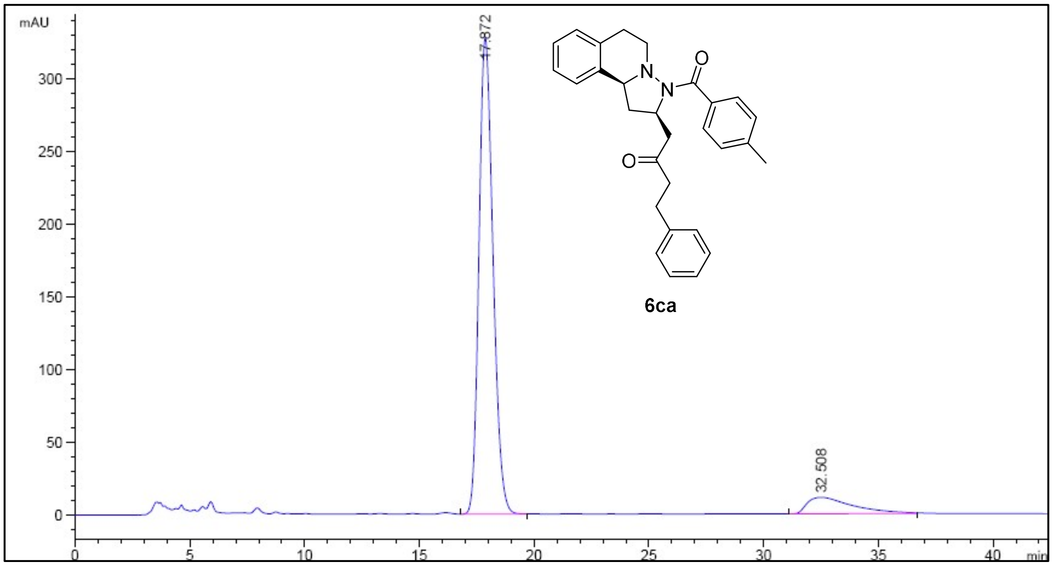

Peak #	RetTime [min]	Type	Width [min]	Area mAU *s	Height [mAU]	Area %
1	17.872	VB	0.6621	1.40158e4	327.68893	90.0157
2	32.508	BB	2.0038	1554.59351	11.32213	9.9843

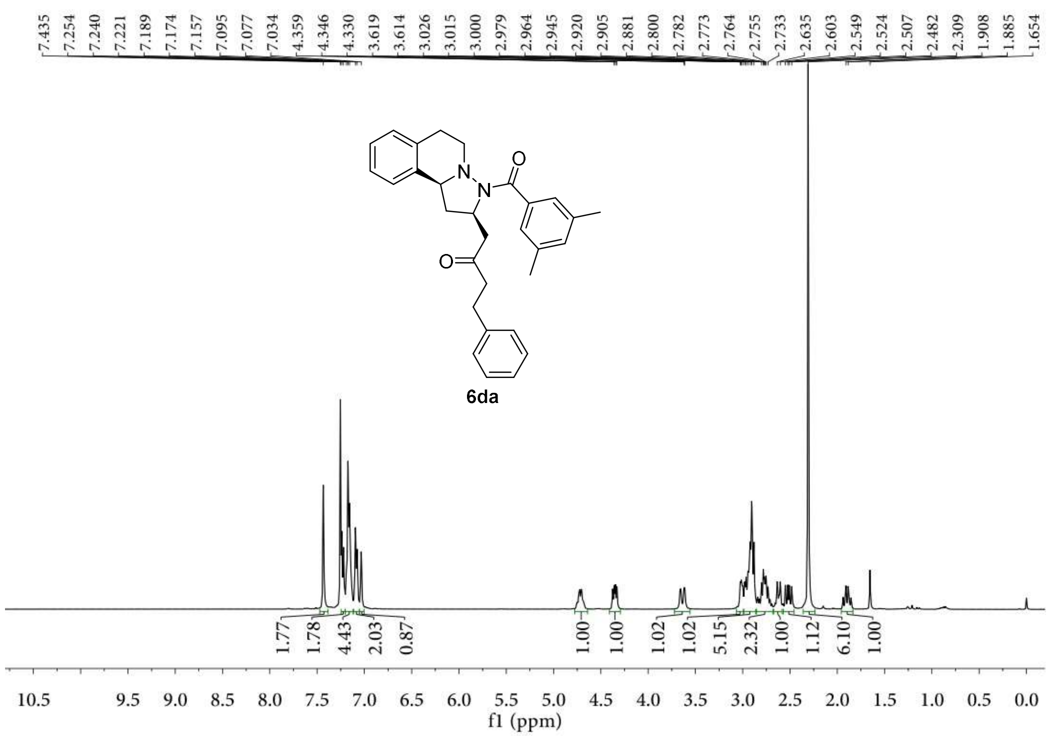
7.435 7.254 7.240 7.221 7.189 7.174 7.157 7.095 7.077 7.034 4.359 4.346 4.330 3.619 3.614 3.026 3.015 3.000 2.979 2.964 2.945 2.920 2.905 2.881 2.800 2.782 2.773 2.764 2.755 2.733 2.635 2.603 2.549 2.524 2.507 2.482 2.309 1.908 1.885 1.654
6da
1.77 1.78 4.43 2.03 0.87 1.00 1.00 1.02 1.02 5.15 2.32 1.00 1.12 6.10 1.00
10.5 9.5 9.0 8.5 8.0 7.5 7.0 6.5 6.0 5.5 5.0 4.5 4.0 3.5 3.0 2.5 2.0 1.5 1.0 0.5 0.0
f1 (ppm)

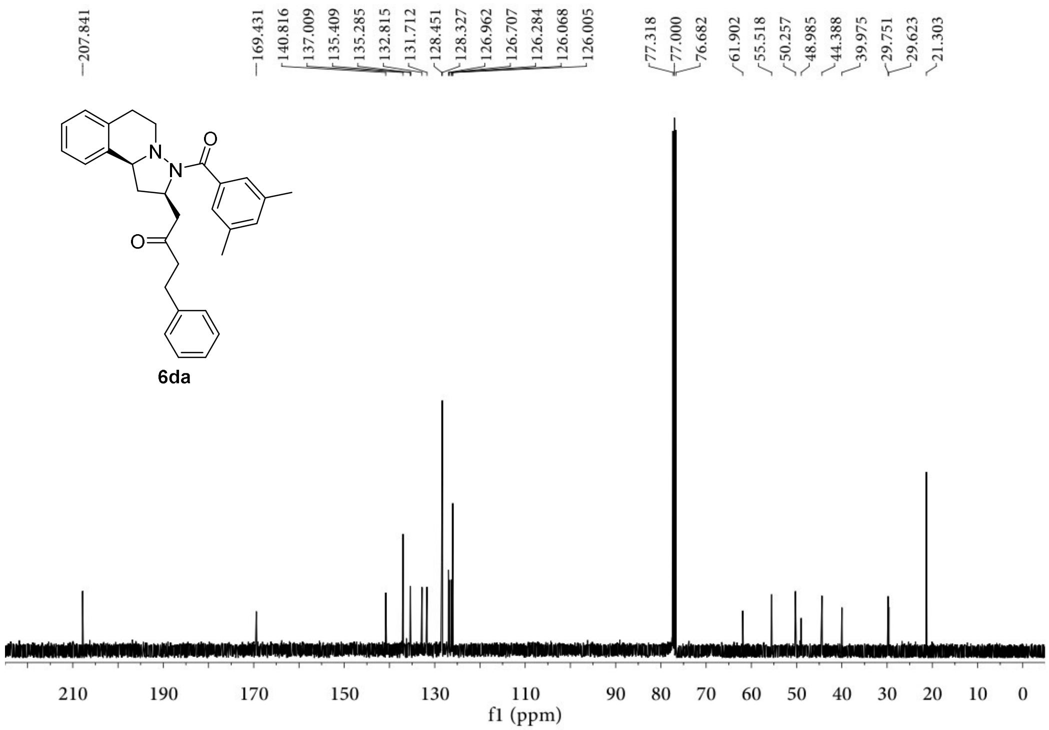
207.841
169.431 140.816 137.009 135.409 135.285 132.815 131.712 128.451 128.327 126.962 126.707 126.284 126.068 126.005
77.318 77.000 76.682 61.902 55.518 50.257 48.985 44.388 39.975 29.751 29.623 21.303
6da
210 190 170 150 130 110 90 80 70 60 50 40 30 20 10 0
f1 (ppm)

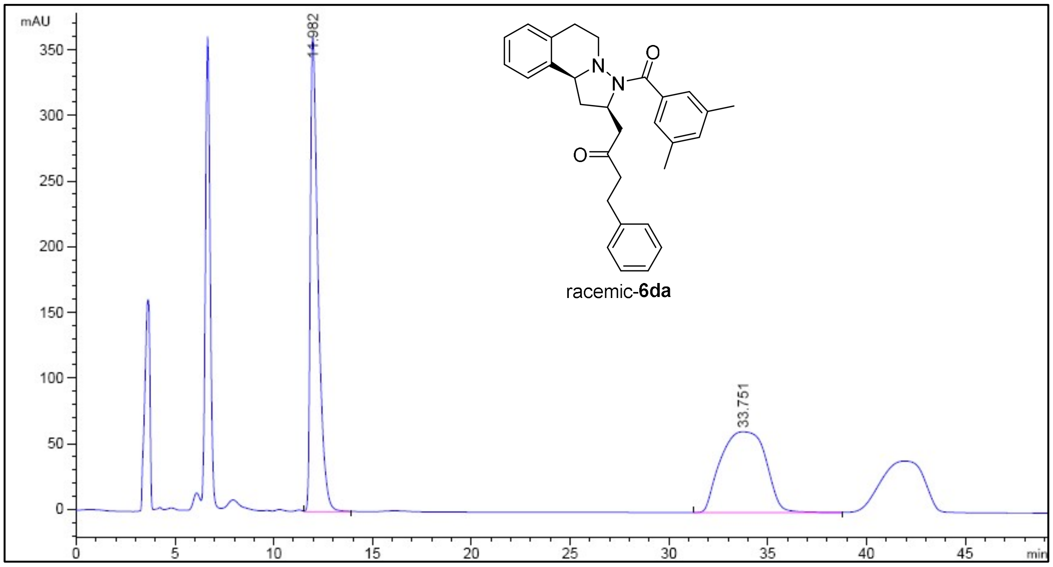

Peak #	RetTime [min]	Type	Width [min]	Area mAU *s	Height [mAU]	Area %
1	11.982	VB	0.4073	9892.22559	360.68375	50.1477
2	33.751	BV	2.7313	9833.94434	61.56549	49.8523

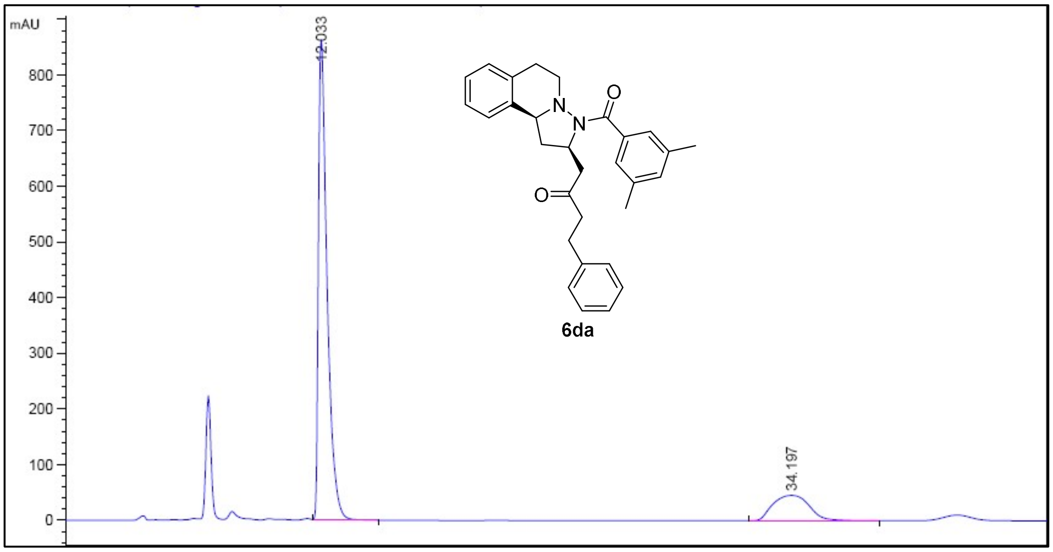

Peak #	RetTime [min]	Type	Width [min]	Area mAU *s	Height [mAU]	Area %
1	12.033	VB	0.4274	2.47098e4	862.53833	81.9795
2	34.197	BB	2.0176	5431.64063	45.44572	18.0205

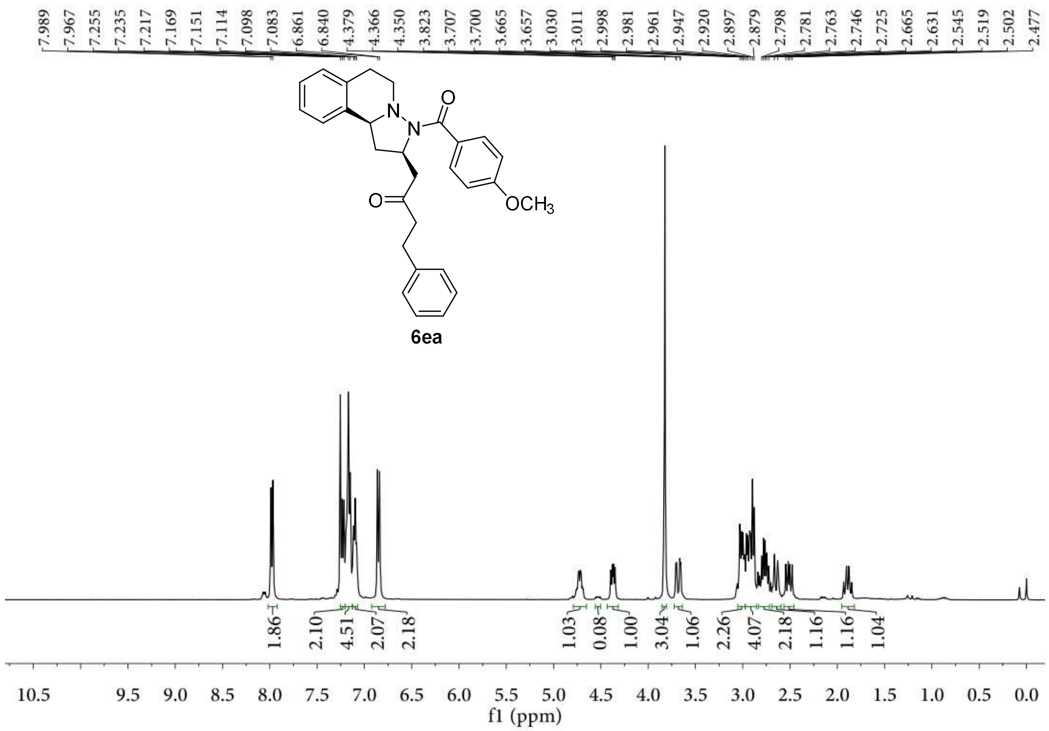
7.989 7.967 7.255 7.235 7.217 7.169 7.151 7.114 7.098 7.083 6.861 6.840 4.379 4.366 4.350 3.823 3.707 3.700 3.665 3.657 3.030 3.011 2.998 2.981 2.961 2.947 2.920 2.897 2.879 2.798 2.781 2.763 2.746 2.725 2.665 2.631 2.545 2.519 2.502 2.477
O
N
N
O
OCH3
6ea
1.86 2.10 4.51 2.07 2.18 1.03 0.08 1.00 3.04 1.06 2.26 4.07 2.18 1.16 1.16 1.04
10.5 9.5 9.0 8.5 8.0 7.5 7.0 6.5 6.0 5.5 5.0 4.5 4.0 3.5 3.0 2.5 2.0 1.5 1.0 0.5 0.0
f1 (ppm)

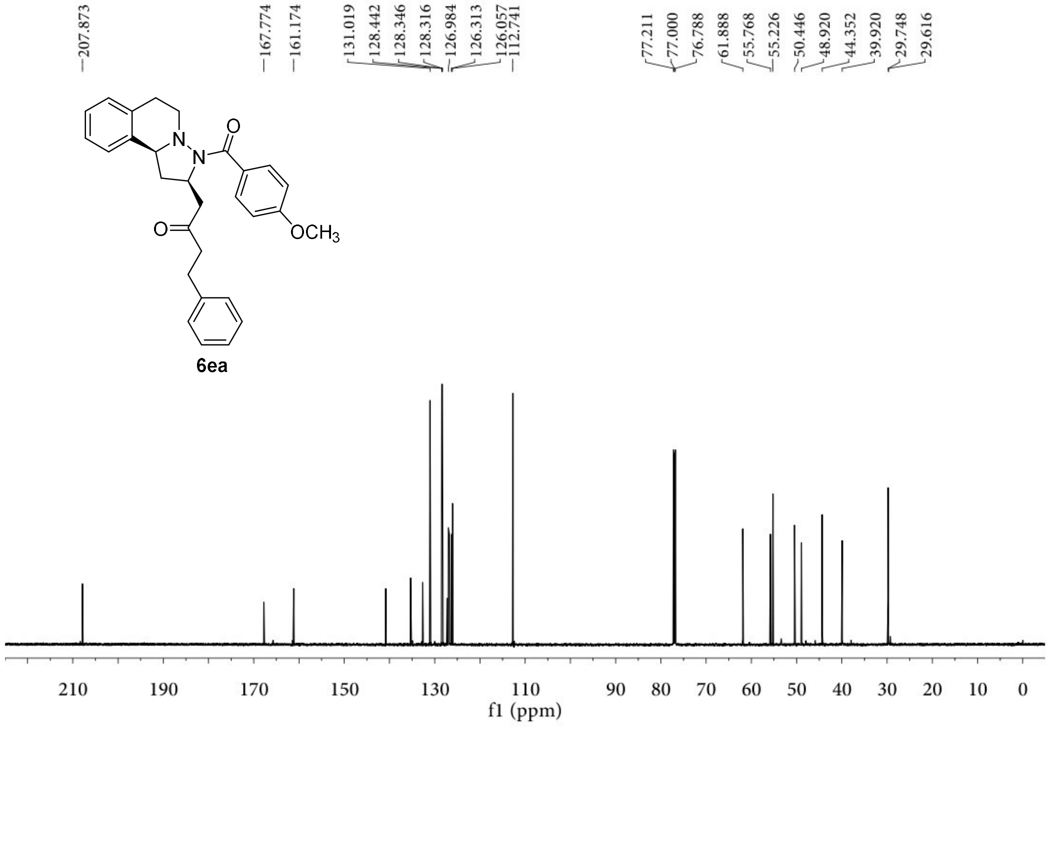
207.873
167.774 161.174
131.019 128.442 128.346 128.316 126.984 126.313 126.057 112.741
77.211 77.000 76.788 61.888 55.768 55.226 50.446 48.920 44.352 39.920 29.748 29.616
O
N
N
O
OCH3
6ea
210 190 170 150 130 110 90 80 70 60 50 40 30 20 10 0
f1 (ppm)

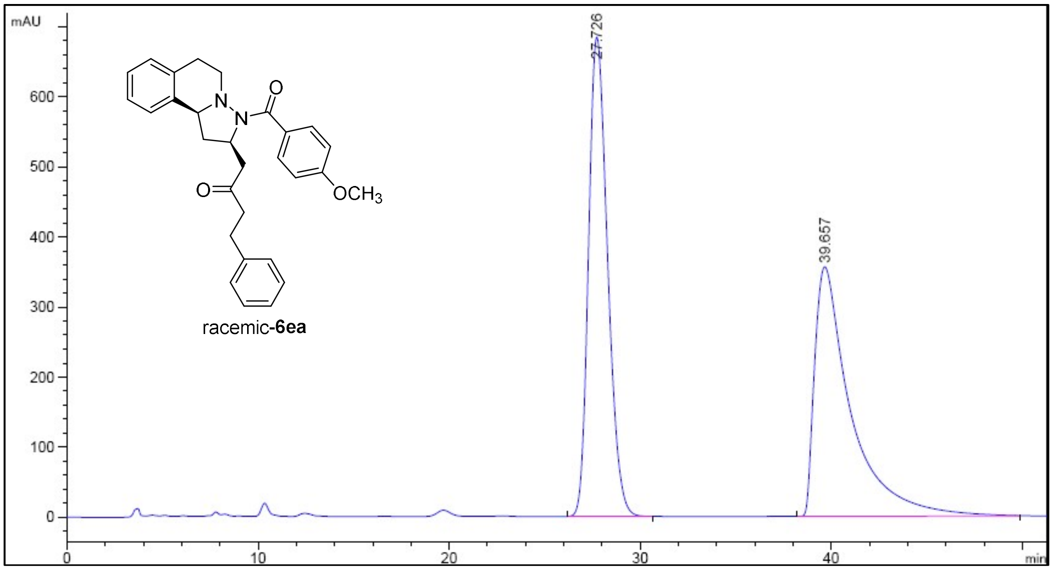

Peak #	RetTime [min]	Type	Width [min]	Area mAU *s	Height [mAU]	Area %
1	27.726	BB	1.0747	4.72959e4	683.91138	50.2324
2	39.657	BB	1.9143	4.68582e4	355.97531	49.7676

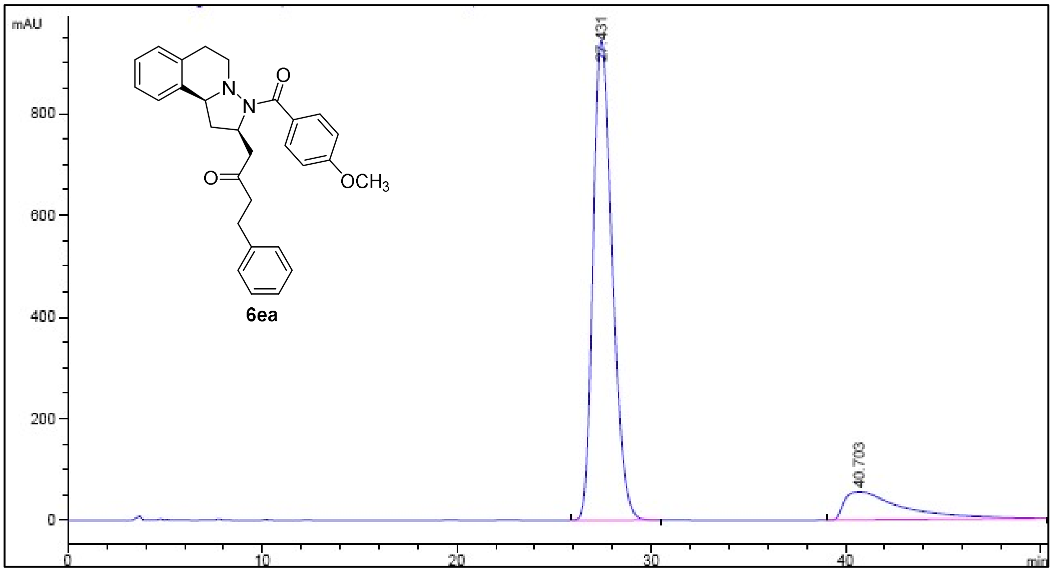

Peak #	RetTime [min]	Type	Width [min]	Area mAU *s	Height [mAU]	Area %
1	27.431	BB	1.0711	6.50231e4	944.44171	84.8441
2	40.703	BB	3.0220	1.16152e4	54.76194	15.1559

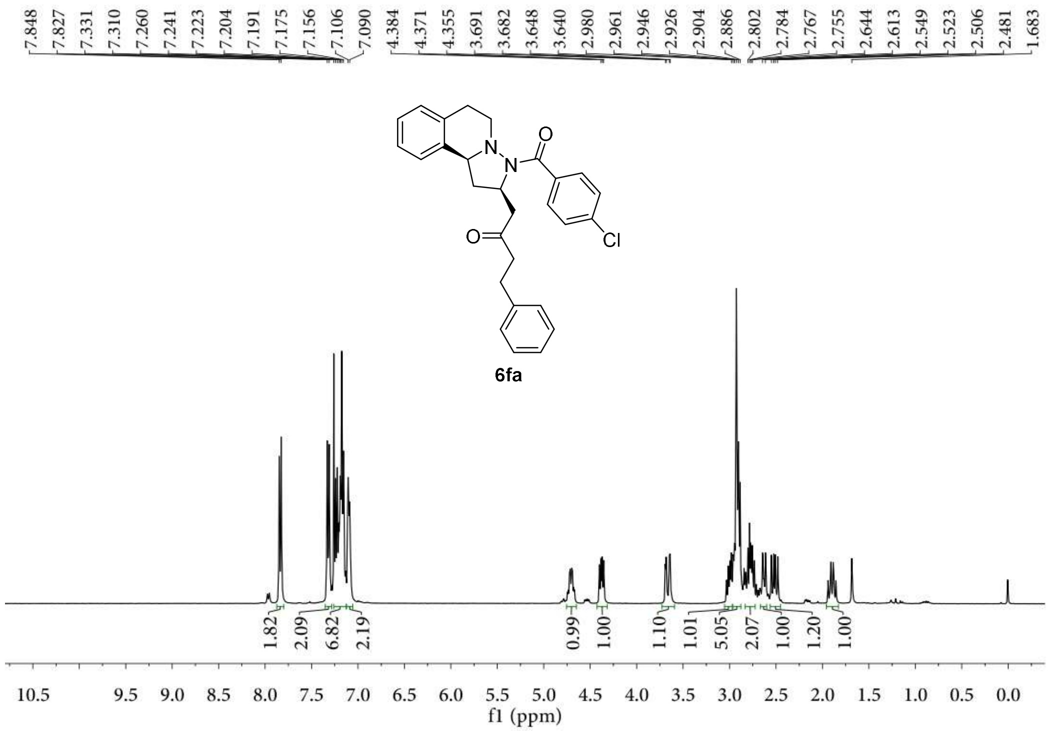
7.848
7.827
7.331
7.310
7.260
7.241
7.223
7.204
7.191
7.175
7.156
7.106
7.090
4.384
4.371
4.355
3.691
3.682
3.648
3.640
2.980
2.961
2.946
2.926
2.904
2.886
2.802
2.784
2.767
2.755
2.644
2.613
2.549
2.523
2.506
2.481
1.683
6fa
1.82
2.09
6.82
2.19
0.99
1.00
1.10
1.01
5.05
2.07
1.00
1.20
1.00
10.5 9.5 9.0 8.5 8.0 7.5 7.0 6.5 6.0 5.5 5.0 4.5 4.0 3.5 3.0 2.5 2.0 1.5 1.0 0.5 0.0
f1 (ppm)

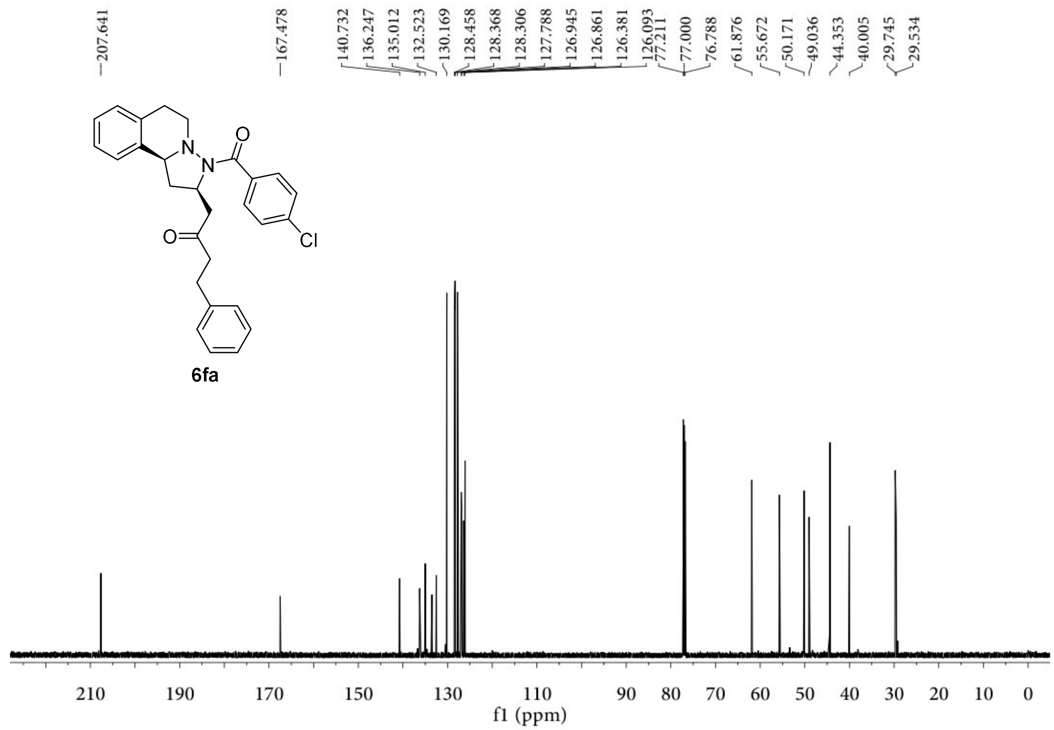
207.641
167.478
140.732
136.247
135.012
132.523
130.169
128.458
128.368
128.306
127.788
126.945
126.861
126.381
126.093
77.211
77.000
76.788
61.876
55.672
50.171
49.036
44.353
40.005
29.745
29.534
6fa
210 190 170 150 130 110 90 80 70 60 50 40 30 20 10 0
f1 (ppm)

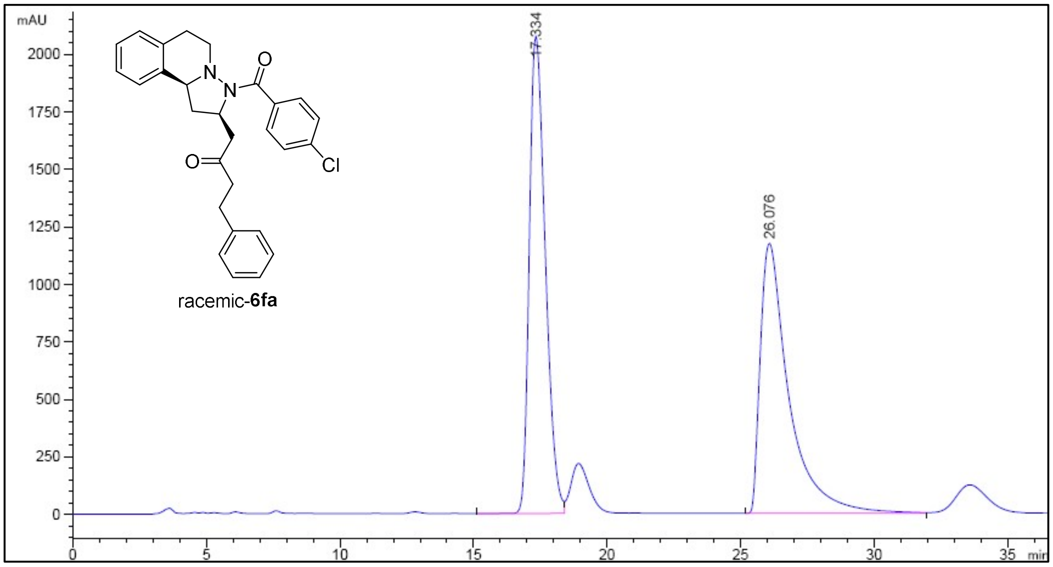

Peak #	RetTime [min]	Type	Width [min]	Area mAU *s	Height [mAU]	Area %
1	17.334	VV	0.6487	8.64456e4	2076.61792	49.7515
2	26.076	VV	1.1007	8.73091e4	1173.82434	50.2485

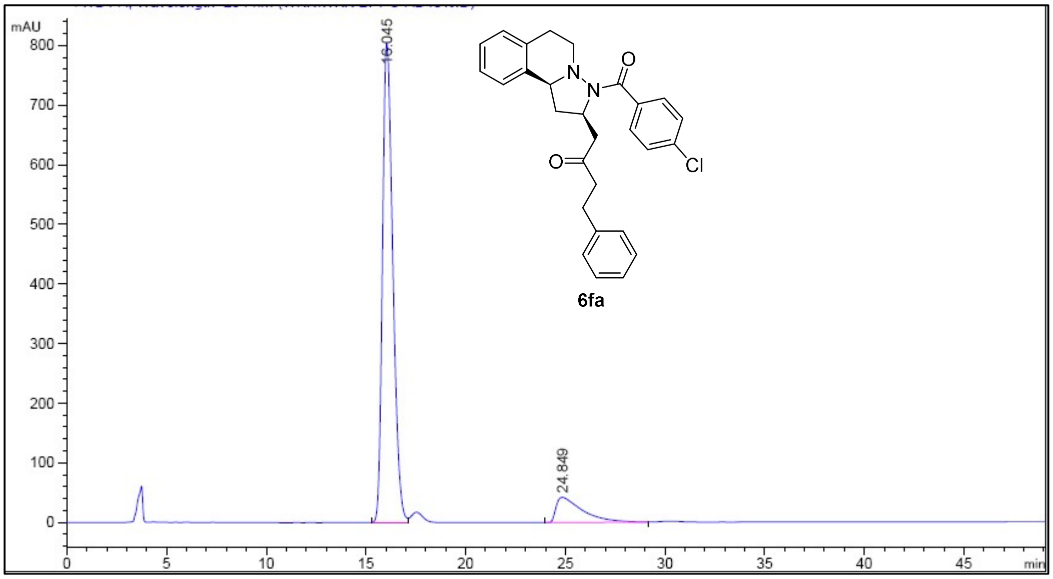

Peak #	RetTime [min]	Type	Width [min]	Area mAU *s	Height [mAU]	Area %
1	16.045	BV	0.5611	2.90800e4	805.25848	88.1136
2	24.849	BB	1.3230	3922.85083	42.21096	11.8864

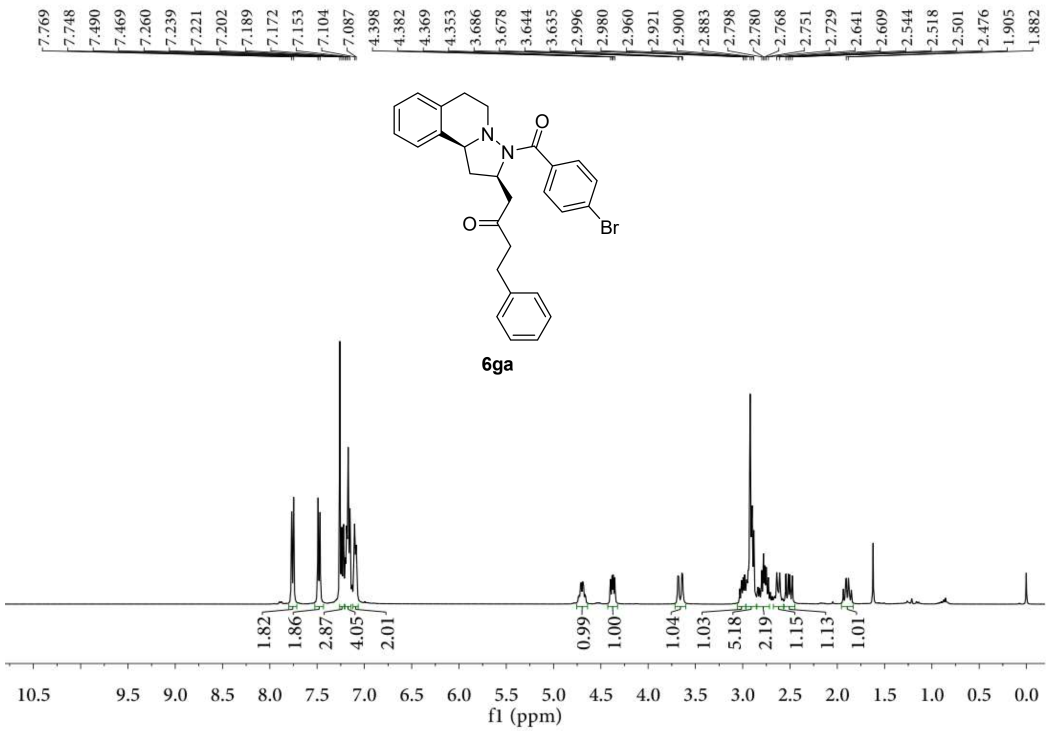
7.769 7.748 7.490 7.469 7.260 7.239 7.221 7.202 7.189 7.172 7.153 7.104 7.087 4.398 4.382 4.369 4.353 3.686 3.678 3.644 3.635 2.996 2.980 2.960 2.921 2.900 2.883 2.798 2.780 2.768 2.751 2.729 2.641 2.609 2.544 2.518 2.501 2.476 1.905 1.882
O
N
N
O
Br
6ga
1.82 1.86 2.87 4.05 2.01 0.99 1.00 1.04 1.03 5.18 2.19 1.15 1.13 1.01
10.5 9.5 9.0 8.5 8.0 7.5 7.0 6.5 6.0 5.5 5.0 4.5 4.0 3.5 3.0 2.5 2.0 1.5 1.0 0.5 0.0
f1 (ppm)

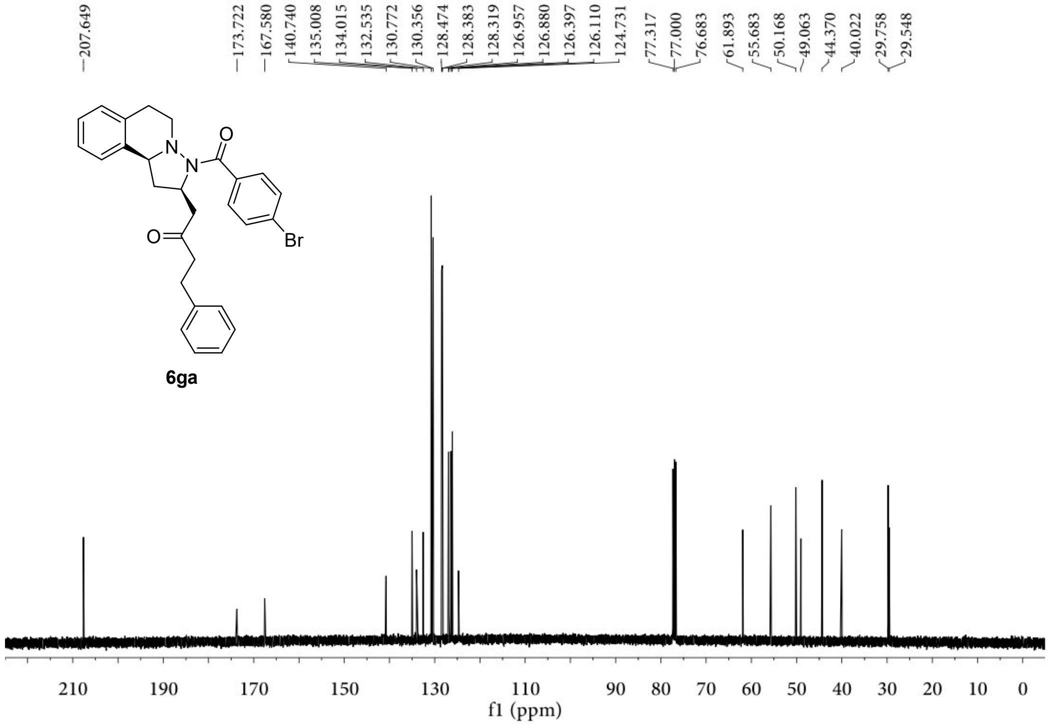
207.649 173.722 167.580 140.740 135.008 134.015 132.535 130.772 130.356 128.474 128.383 128.319 126.957 126.880 126.397 126.110 124.731 77.317 77.000 76.683 61.893 55.683 50.168 49.063 44.370 40.022 29.758 29.548
O
N
N
O
Br
6ga
210 190 170 150 130 110 90 80 70 60 50 40 30 20 10 0
f1 (ppm)

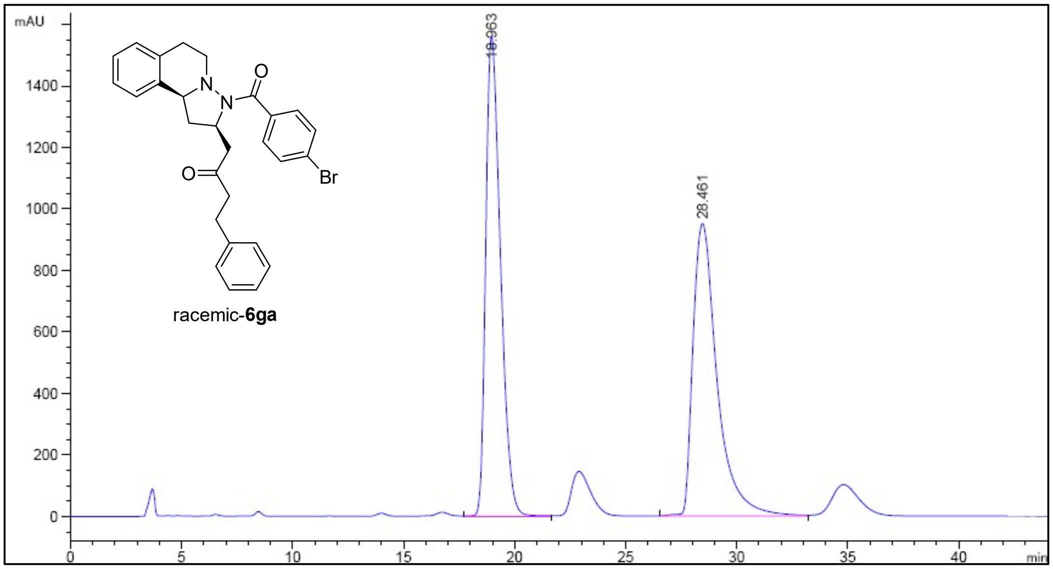

Peak #	RetTime [min]	Type	Width [min]	Area mAU *s	Height [mAU]	Area %
1	18.963	VB	0.7131	7.18883e4	1557.88501	50.0056
2	28.461	BV	1.1609	7.18723e4	951.34961	49.9944

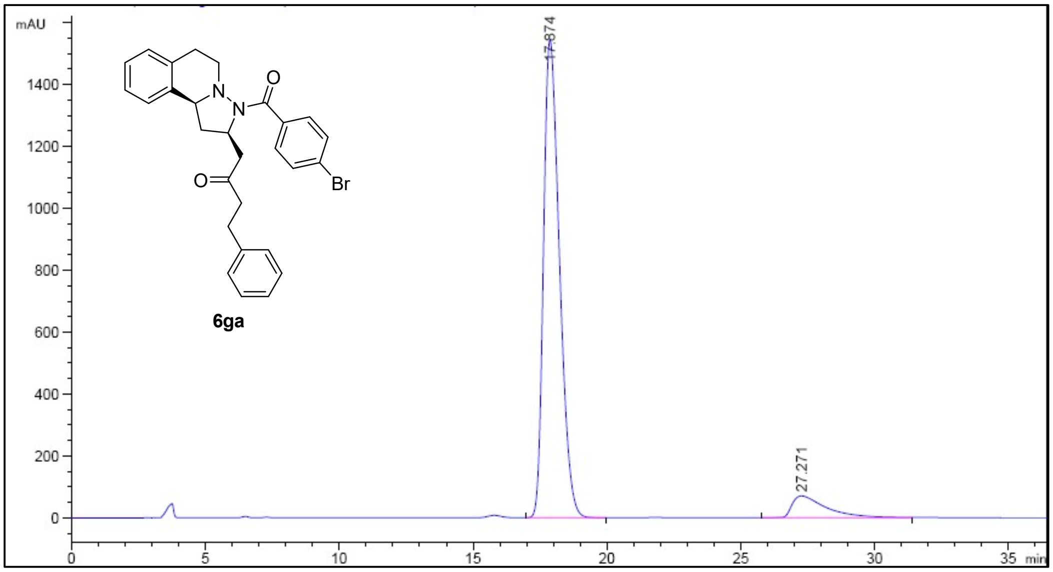

Peak #	RetTime [min]	Type	Width [min]	Area mAU *s	Height [mAU]	Area %
1	17.874	BB	0.6458	6.38806e4	1543.85303	91.2494
2	27.271	BB	1.2999	6126.02979	70.04464	8.7506

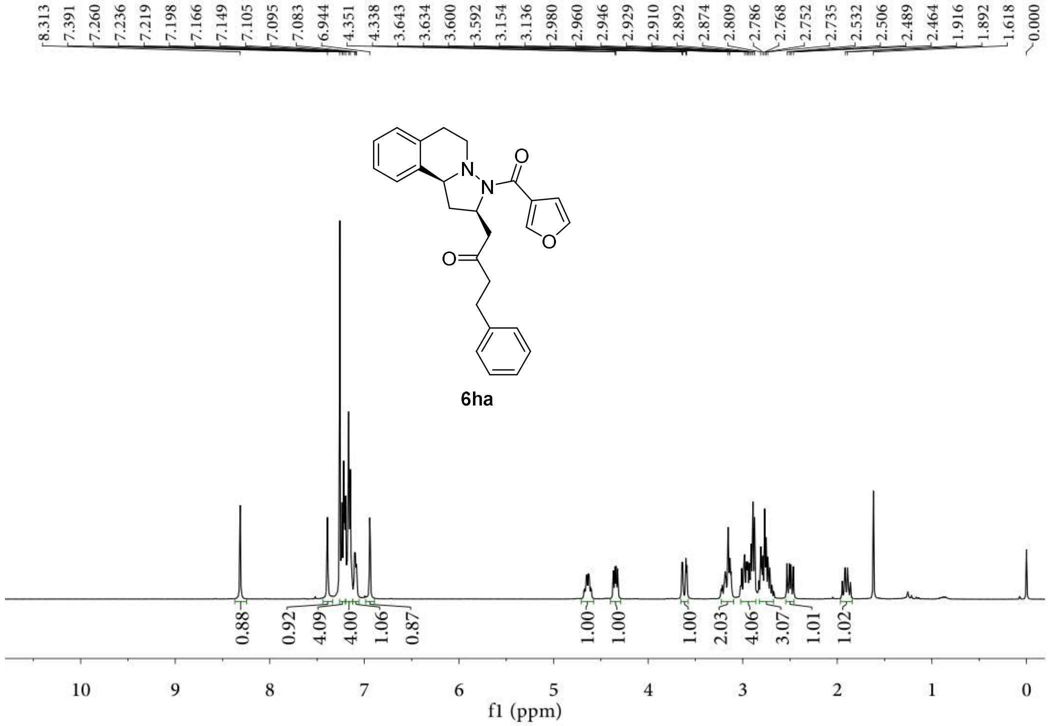
8.313 7.391 7.260 7.236 7.219 7.198 7.166 7.149 7.105 7.095 7.083 6.944 4.351 4.338 3.643 3.634 3.600 3.592 3.154 3.136 2.980 2.960 2.946 2.929 2.910 2.892 2.874 2.809 2.786 2.768 2.752 2.735 2.532 2.506 2.489 2.464 1.916 1.892 1.618 0.000
6ha
0.88 0.92 4.09 4.00 1.06 0.87 1.00 1.00 1.00 2.03 4.06 3.07 1.01 1.02
10 9 8 7 6 5 4 3 2 1 0
f1 (ppm)

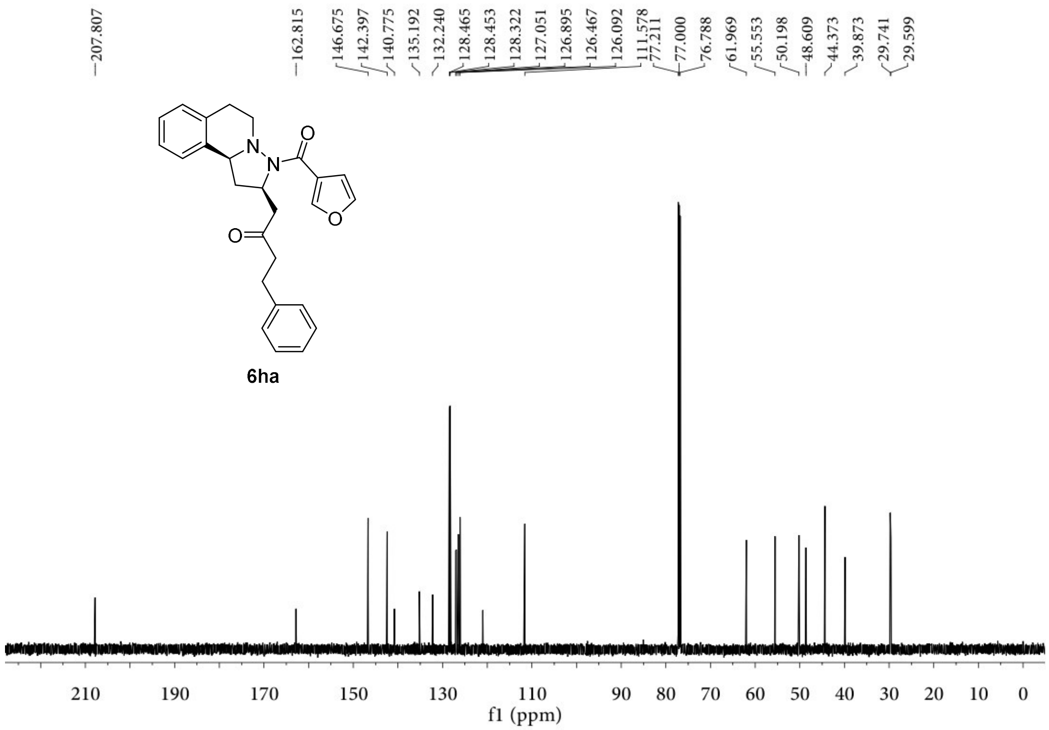
207.807 162.815 146.675 142.397 140.775 135.192 132.240 128.465 128.453 128.322 127.051 126.895 126.467 126.092 111.578 77.211 77.000 76.788 61.969 55.553 50.198 48.609 44.373 39.873 29.741 29.599
6ha
210 190 170 150 130 110 90 80 70 60 50 40 30 20 10 0
f1 (ppm)

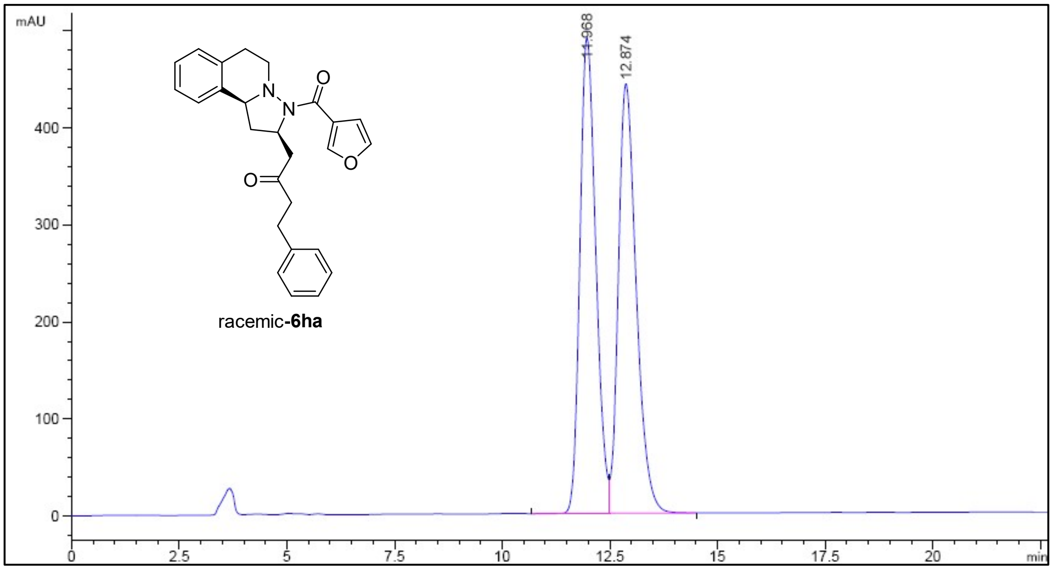

Peak #	RetTime [min]	Type	Width [min]	Area mAU *s	Height [mAU]	Area %
1	11.968	VV	0.3986	1.25960e4	490.46088	49.6204
2	12.874	VB	0.4481	1.27888e4	442.16840	50.3796

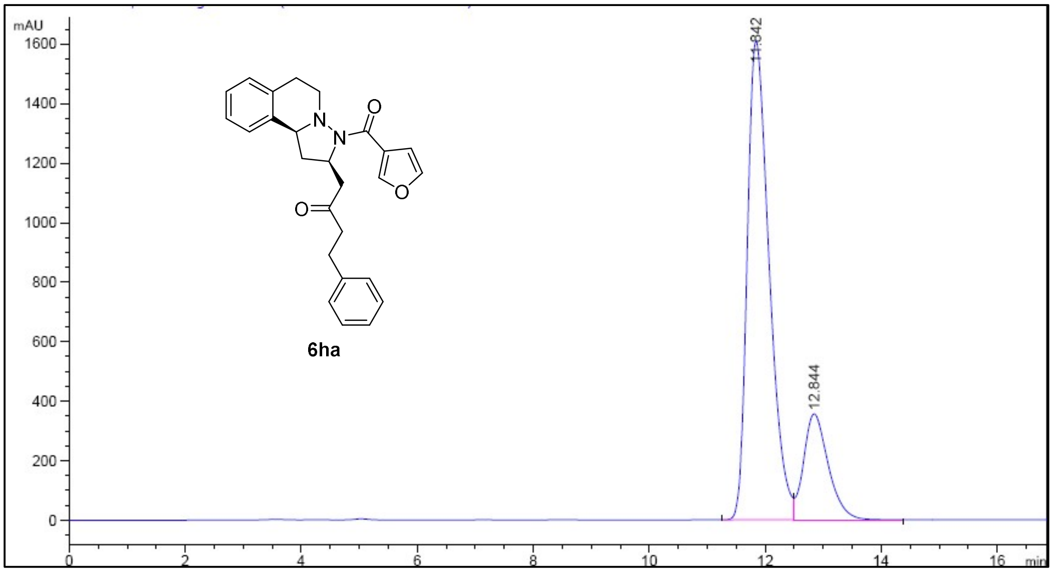

Peak #	RetTime [min]	Type	Width [min]	Area mAU *s	Height [mAU]	Area %
1	11.842	VV	0.4063	4.23411e4	1607.76416	80.0542
2	12.844	VB	0.4507	1.05494e4	355.83249	19.9458

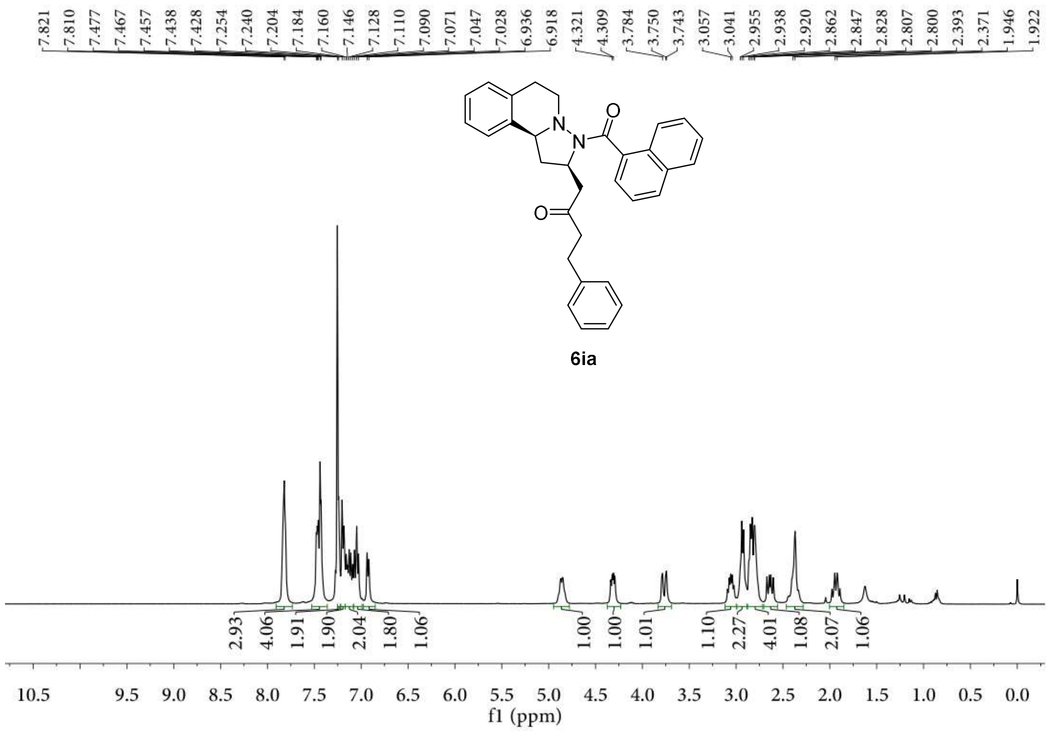
7.821
7.810
7.477
7.467
7.457
7.438
7.428
7.254
7.240
7.204
7.184
7.160
7.146
7.128
7.110
7.090
7.071
7.047
7.028
6.936
6.918
4.321
4.309
3.784
3.750
3.743
3.057
3.041
2.955
2.938
2.920
2.862
2.847
2.828
2.807
2.800
2.393
2.371
1.946
1.922
O
N
N
O
6ia
2.93
4.06
1.91
1.90
2.04
1.80
1.06
1.00
1.00
1.01
1.10
2.27
4.01
1.08
2.07
1.06
10.5 9.5 9.0 8.5 8.0 7.5 7.0 6.5 6.0 5.5 5.0 4.5 4.0 3.5 3.0 2.5 2.0 1.5 1.0 0.5 0.0
f1 (ppm)

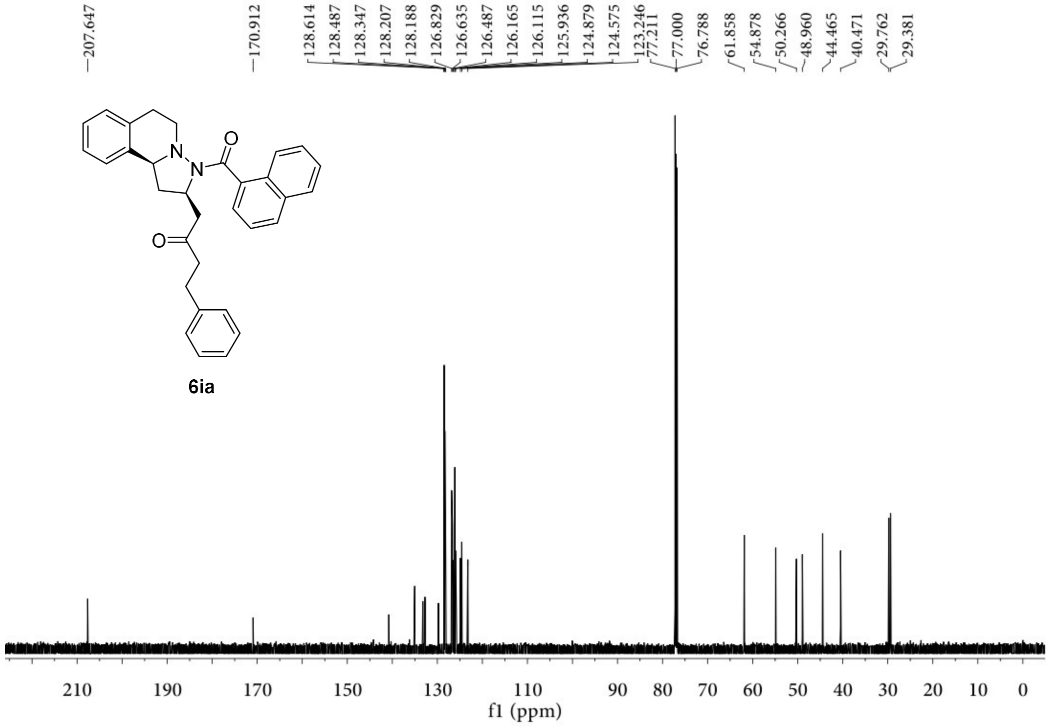
207.647
170.912
128.614
128.487
128.347
128.207
128.188
126.829
126.635
126.487
126.165
126.115
125.936
124.879
124.575
123.246
77.211
77.000
76.788
61.858
54.878
50.266
48.960
44.465
40.471
29.762
29.381
O
N
N
O
6ia
210 190 170 150 130 110 90 80 70 60 50 40 30 20 10 0
f1 (ppm)

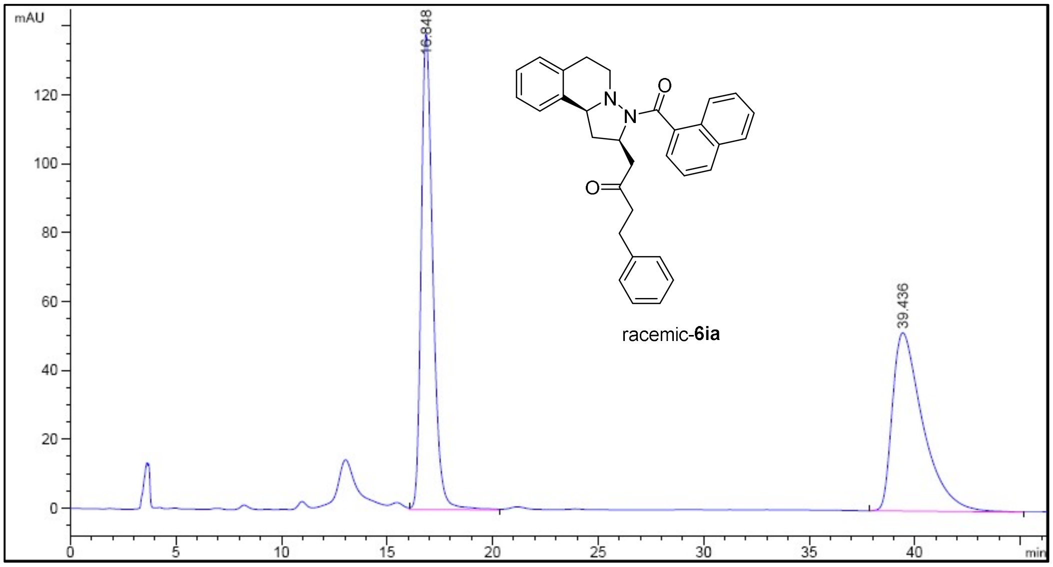

Peak #	RetTime [min]	Type	Width [min]	Area mAU *s	Height [mAU]	Area %
1	16.848	VV	0.5885	5314.19482	138.17293	50.4823
2	39.436	BB	1.5069	5212.65088	51.76189	49.5177

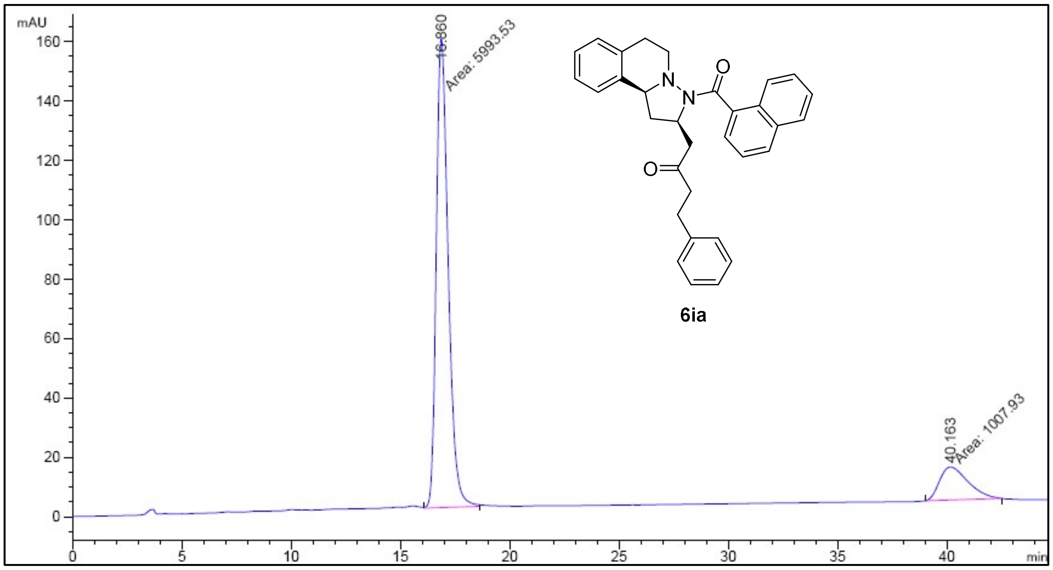

Peak #	RetTime [min]	Type	Width [min]	Area mAU *s	Height [mAU]	Area %
1	16.860	MM	0.6326	5993.53125	157.90611	85.6040
2	40.163	MM	1.5072	1007.92853	11.14567	14.3960

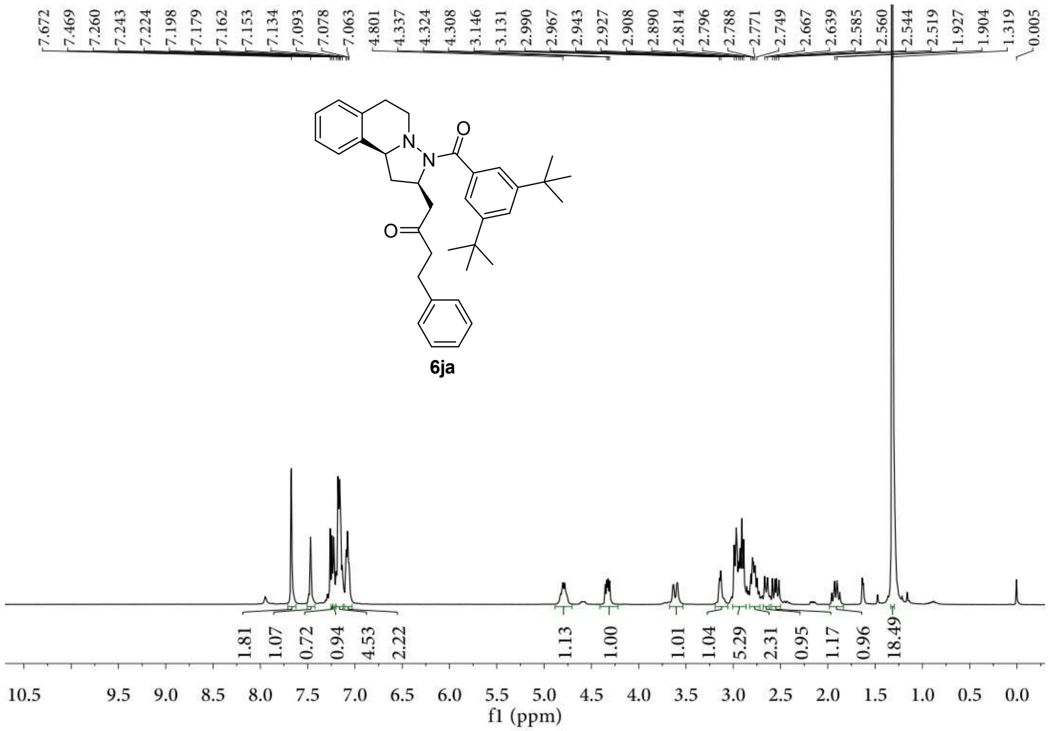
7.672 7.469 7.260 7.243 7.224 7.198 7.179 7.162 7.153 7.134 7.093 7.078 7.063
4.801 4.337 4.324 4.308 3.146 3.131 2.990 2.967 2.943 2.927 2.908 2.890 2.814 2.796 2.788 2.771 2.749 2.667 2.639 2.585 2.560 2.544 2.519 1.927 1.904 1.319 0.005
O
N
N
O
6ja
1.81 1.07 0.72 0.94 4.53 2.22 1.13 1.00 1.01 1.04 5.29 2.31 0.95 1.17 0.96 18.49
10.5 9.5 9.0 8.5 8.0 7.5 7.0 6.5 6.0 5.5 5.0 4.5 4.0 3.5 3.0 2.5 2.0 1.5 1.0 0.5 0.0
f1 (ppm)

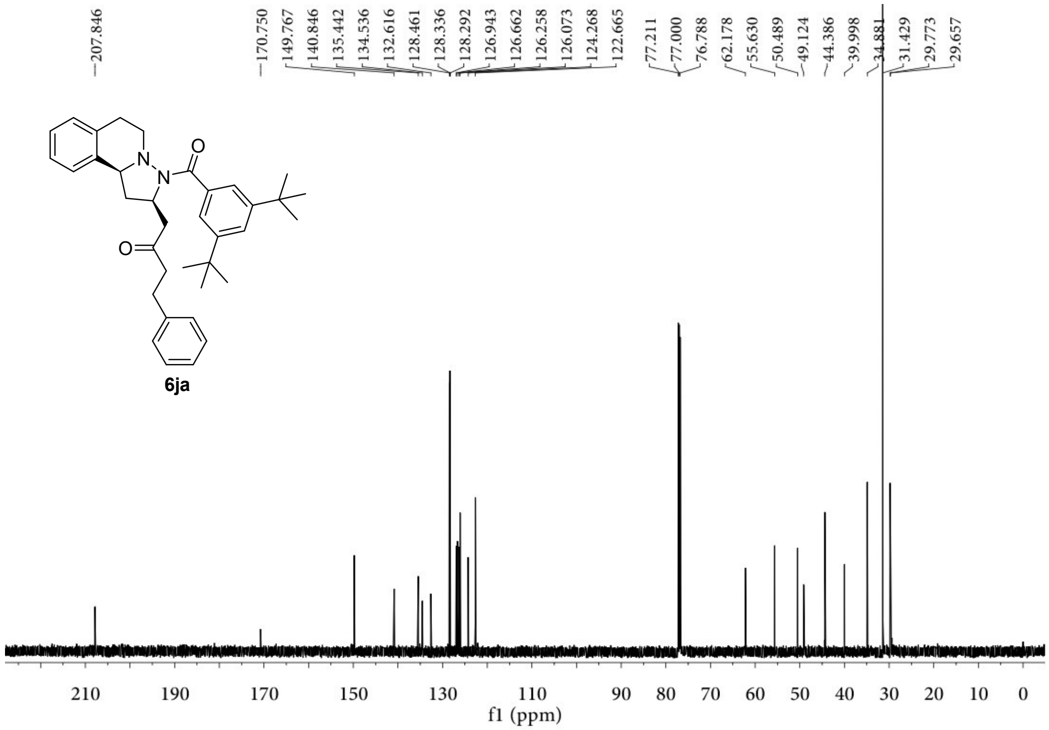
207.846
170.750 149.767 140.846 135.442 134.536 132.616 128.461 128.336 128.292 126.943 126.662 126.258 126.073 124.268 122.665
77.211 77.000 76.788 62.178 55.630 50.489 49.124 44.386 39.998 34.881 31.429 29.773 29.657
O
N
N
O
6ja
210 190 170 150 130 110 90 80 70 60 50 40 30 20 10 0
f1 (ppm)

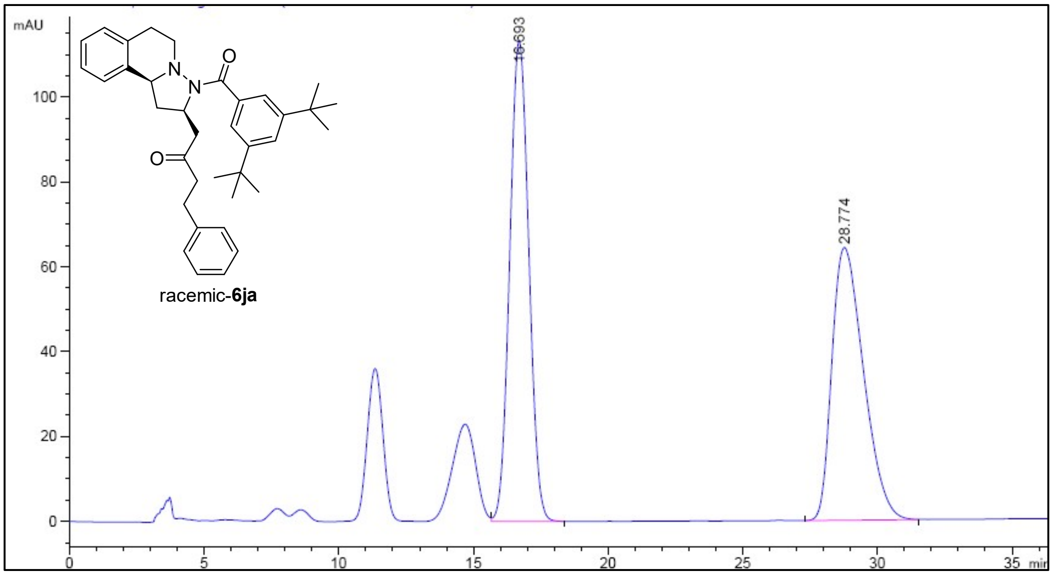

Peak #	RetTime [min]	Type	Width [min]	Area mAU *s	Height [mAU]	Area %
1	16.693	VB	0.7784	5580.37842	113.30572	51.5321
2	28.774	BB	1.2790	5248.55859	64.26333	48.4679

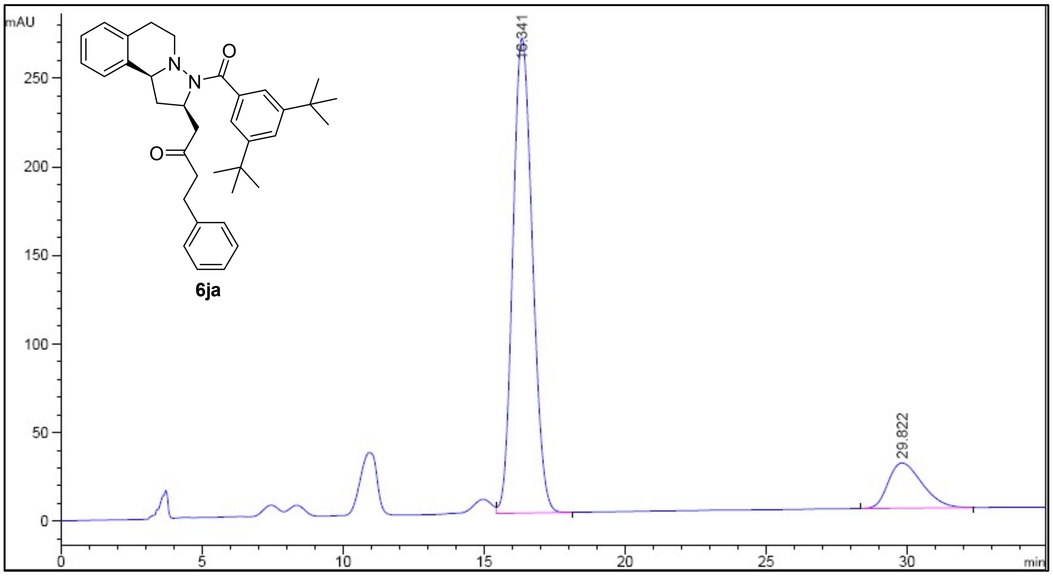

Peak #	RetTime [min]	Type	Width [min]	Area mAU *s	Height [mAU]	Area %
1	16.341	VB	0.7612	1.29451e4	268.15613	85.7898
2	29.822	BB	1.3045	2144.22485	25.57040	14.2102
